# Studying Cation Exchange in {Cr_7_Co} Pseudorotaxanes: Preparatory Studies for Making Hybrid Molecular Machines

**DOI:** 10.1002/chem.202400432

**Published:** 2024-06-11

**Authors:** Tom S. Bennett, Niklas Geue, Grigore A. Timco, George F. S. Whitehead, Inigo J. Vitorica‐Yrezabal, Perdita E. Barran, Eric J. L. McInnes, Richard E. P. Winpenny

**Affiliations:** ^1^ Department of Chemistry The University of Manchester The University of Manchester Oxford Road Manchester M13 9PL UK; ^2^ Department of Chemistry Michael Barber Centre for Collaborative Mass Spectrometry Manchester Institute of Biotechnology The University of Manchester 131 Princess Street Manchester M1 7DN UK

**Keywords:** host-guest, rotaxane, metallosupramolecular, paramagnetic NMR spectroscopy, collision induced mass spectrometry

## Abstract

In the design of dynamic supramolecular systems used in molecular machines, it is important to understand the binding preferences between the macrocycle and stations along the thread. Here, we apply ^1^H NMR spectroscopy to investigate the relative stabilities of a series of linear alkylammonium templated pseudorotaxanes with the general formula [H_2_NRR’][Cr_7_CoF_8_(O_2_CCH_2_^t^Bu)_16_] by exchanging the cation in solution. Our results show that the pseudorotaxanes are able to exchange threads via a dissociative mechanism. The position of equilibrium is dependent upon the ammonium cation and solvent used. Short chain primary ammonium cations are shown to be far less favourable macrocycle stations than secondary ammonium cations. Collision‐induced dissociation mass spectrometry (CID‐MS) has been used to look at disassembly of the pseudorotaxanes in a solvent‐free environment and stability trends compared to those in acetone‐d6. The energy needed to induce 50 % of the precursor ion loss (*E*_50_) is used and shows a similar trend to the equilibria measured by NMR. The relative stabilities of these hybrid inorganic‐organic pseudo‐rotaxanes are different to those of host‐guest compounds involving crown ethers and this may be valuable for the design of molecular machines.

## Graphical abstract

^1^H NMR spectroscopy is applied to study the relative binding affinities between a {Cr_7_Co} macrocycle (Cr_7_Co=Cr_7_CoF_8_(O_2_CCH_2_^t^Bu)_16_) and a series of linear alkylammonium threads. The ammonium cation is observed to exchange in solution using ^1^H NMR spectroscopy and kinetic studies show that the exchange takes place *via* a dissociative mechanism. The position of the equilibrium is dependent upon the solvent and structure of the ammonium. Collision‐induced dissociation mass spectrometry (CID‐MS) is used to measure the energy needed to induce 50 % of the precursor ion loss (*E*_50_). The trend in pseudorotaxane stabilities is similar when compared to the NMR trends in acetone‐*d*_6_. These preliminary studies yield information which are valuable in the future design of hybrid inorganic‐organic molecular machines.

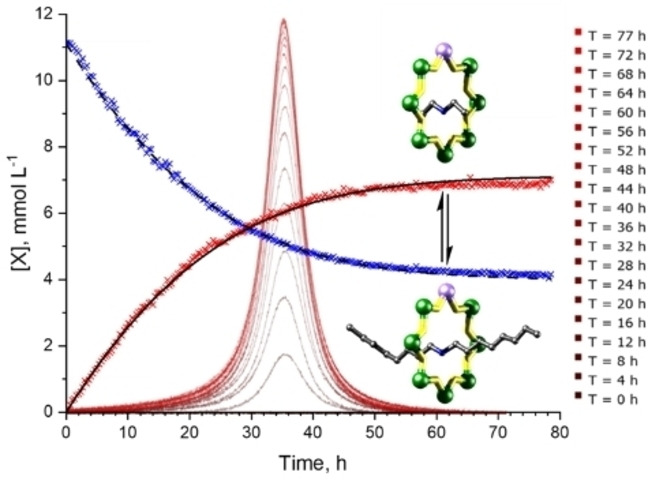

## Introduction

Synthetic strategies such as slippage[^1^, ^2^] have been used to construct supramolecular assemblies such as rotaxanes and pseudorotaxanes. Slippage is where size complementarity between a macrocycle and stoppers on a thread can allow rotaxane formation with stabilising non‐covalent bonding interactions between functional groups on the two components providing a thermodynamic driving force. The stability of rotaxanes and pseudorotaxanes to disassembly can be increased by optimising these non‐covalent interactions between the macrocycle and thread.^[3]^ Taking this further, an understanding of the relative binding affinities of different sites or stations on the thread can be applied to develop dynamic interlocked systems. For example, non‐covalent interactions between macrocycles and stations have been altered chemically,[^4^, ^5^, ^6^, ^7^] electrochemically[^8^, ^9^, ^10^] and photochemically^[11]^ to construct molecular shuttles. Understanding which structural motifs stabilise interactions between thread and macrocycle is crucial in designing alternative pathways to synthesis or even developing molecular frameworks capable of protecting functional groups.[^12^, ^13^]

The vast majority of rotaxanes involve organic components as both thread and ring. We have previously reported hybrid inorganic‐organic rotaxanes,[^14^, ^15^] and used them as building blocks for larger supramolecules.[^16^, ^17^, ^18^] Understanding how the components interact is essential in using these supramolecules as molecular machines. Hybrid rotaxanes have not been studied in such depth, and here we report studies intended to establish how these hybrid rotaxanes could be used.

**Figure 1:**
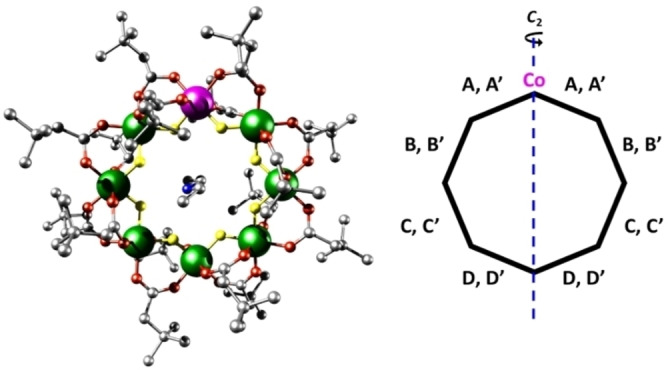
Left: Single Crystal XRD structure of **Et_2_NH_2_** **⋅ 1** where: C–grey, O–red, N–blue, F–yellow, Cr–green, Co–purple. H atoms have been omitted for clarity. Right: A schematic of **1** used to label equatorial (X) and axial (X’) carboxylates with respect to the Co^II^ ion.

Unlike a rotaxane, a pseudorotaxane contains no mechanical bond interlocking the ‘thread’ and ‘wheel’ components of the molecule, and therefore the energy barrier to disassembly may be small enough to overcome at ambient temperature. Previous studies have observed thread loss *via* a slipping mechanism when collisionally activating a series of hybrid rotaxanes and pseudo‐rotaxanes by tandem mass spectrometry.^[19]^ Here, we investigate the thermodynamic stability of a series of linear alkyl‐amines with the general formula [H_2_NRR’][Cr^III^_7_Co^II^F_8_(O_2_CCH_2_^t^Bu)_16_] (Figure 1) where R and R’ are linear alkyl‐chains (or R’=H where the cation is a primary alklyammonium).^[20]^

In this molecule the paramagnetic anionic ring is formed through a templated synthesis about an alkylammonium cation. The {Cr_7_Co} rings were chosen as ^1^H NMR studies are possible due to the very fast electron spin relaxation of Co^II^ in an octahedral coordination geometry dominates over the relatively slow electronic relaxation of Cr^III^ ions which would otherwise cause the NMR spectra to be broadened beyond detection.[^21^, ^22^] We have used 3,3‐dimethylbutanoate ligands on the {Cr_7_Co} rings, in preference to pivalate, as this gives increased solubility in organic solvents, enabling more detailed studies.

## Results and Discussion

The pseudo‐rotaxanes are synthesised *via* a template synthesis involving a secondary amine (see Supplementary Information for details). The structure of [H_2_NEt_2_][_Cr7_Co^II^_F8_(O_2_CCH_2_^t^Bu)_16_] **Et_2_NH_2_** **⋅ 1** is shown in Figure 1. The structures of **Et_2_NH_2_** **⋅ 1**, **Pr_2_NH_2_** **⋅ 1**, **Oc_2_NH_2_** **⋅ 1**, **Allyl_2_NH_2_** **⋅ 1** and **PrNH_3_** **⋅ 1** where Pr=*n*‐propyl, Oc=*n*‐octyl) were solved and are analogous, differing only in the central cation (Table 1).

**Table 1:**
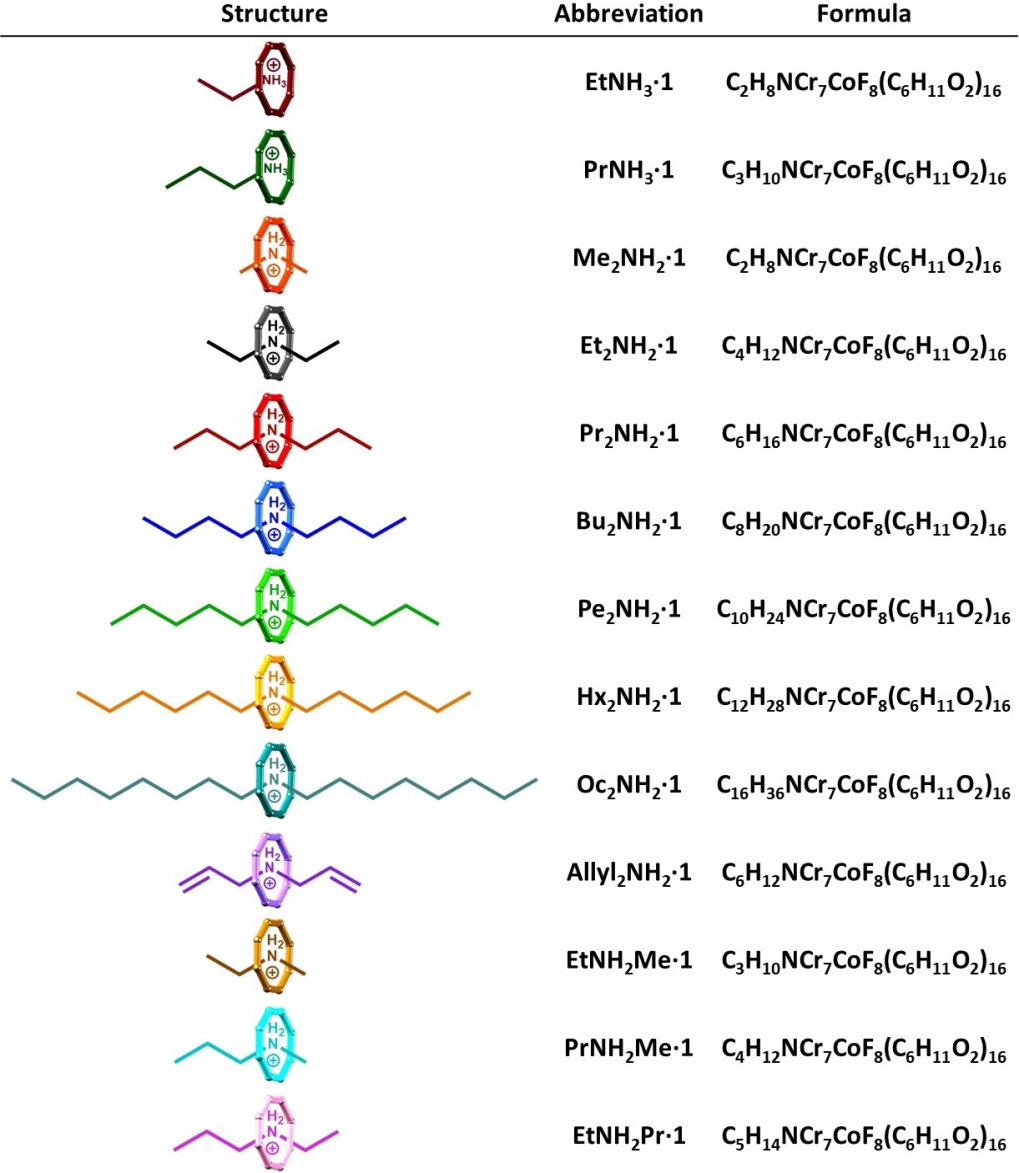
Simplified structures and associated abbreviations for formulas for the symmetric and asymmetric pseudorotaxanes studied.

The {Cr_7_Co} macrocycle is an octagon with each edge bridged by two carboxylates and a single fluoride. On each edge one carboxylate is in an equatorial position, and one axial, with respect to the {Cr_7_Co} ring with the axial carboxylate alternating “up” and “down” around the ring. The Co^II^ is disordered about multiple positions on the ring in the crystal structure.^[23]^

In solution the pseudo‐rotaxanes will have two‐fold symmetry, with the *C*_2_ axis passing through the Co^II^, the N‐atom of the central cation (if the ammonium has two equivalent alkyl chains) and the Cr^III^ in the 5‐position of the octagon. Therefore there are four unique edges to the metal ring, with two distinct carboxylates on each edge. Overall this leads to eight unique carboxylates by NMR spectroscopy. We label these edges as A, A’, B, B’ etc., as shown in Figure 1.

**Figure 2:**
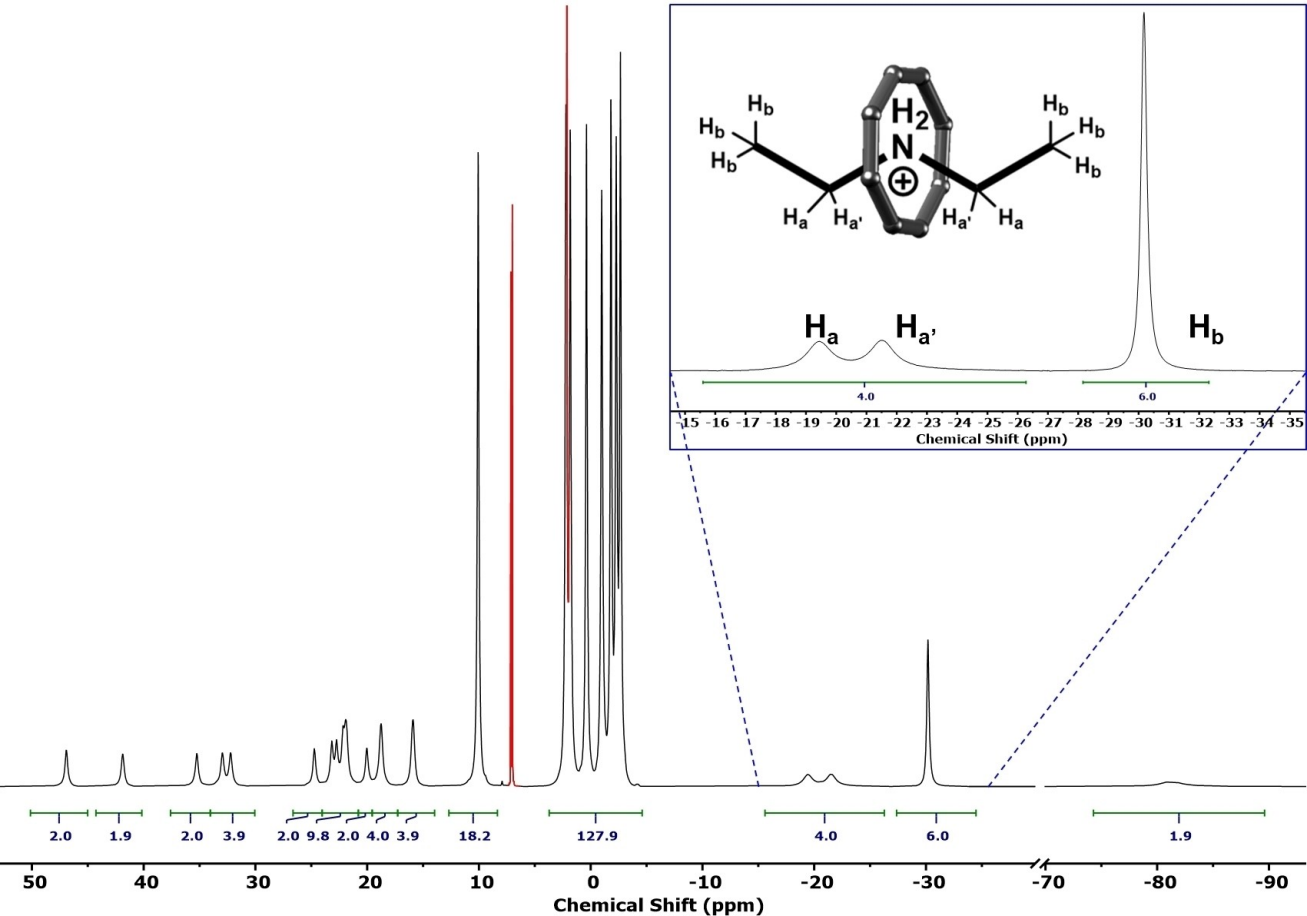
^1^H NMR (500 MHz, 298 K, 30 mM) baseline corrected spectrum of **Et_2_NH_2_** **⋅ 1** in toluene‐*d*_8_. Insert: expanding thread alkyl‐chain signals. The protio‐impurity from the solvent is highlighted in red.

Quantification in paramagnetic NMR is complicated. Chemical shift ranges can be contact shifted beyond the typical spectral window observed in diamagnetic ^1^H NMR experiments (0–10 ppm). Paramagnetic signals are also broader than those produced by diamagnetic compounds, and proton‐proton couplings are often not observed, leading to challenges in spectral assignment. The ^1^H NMR spectrum of **Et_2_NH_2_** **⋅ 1** demonstrates some of the characteristic effects seen within paramagnetic NMR (Figure 2).

The spectrum is comprised of signals in three main regions because of interactions with the paramagnetic centres. Proton signals from the central alkylammonium are shifted upfield to the negative region (−3 to −90 ppm) and appear as singlets within the resolution of the experiment. The most upfield shifted signal is assigned to the ammonium NH protons which from the crystal structure are found at the centre of the cavity. Unexpectedly, the protons on the α‐carbon are less shifted than those on the β‐carbon; this can be explained by the anisotropy of the magnetic susceptibility and because both contact and pseudo‐contact terms are relevant for these protons whereas for protons further from the metal sites only pseudo‐contact shifts are important.

Resonances due to methylene protons on the 3,3‐dimethylbutanoate ligands of the {Cr_7_Co} ring are shifted downfield to 13 to 50 ppm; the methyl resonances from these ligands are shifted to a lesser extent and give signals from −3 to 13 ppm. For both the methylene and methyl 3,3‐dimethylbutanoate protons the *C*_2_ symmetry of the molecule gives rise to eight sets of signals corresponding to the equatorial (A, B, C, D) and axial (A’, B’, C’, D’) carboxylates. Hence, the eight methyl resonances each have a relative integral of 18, analogous to spectra reported previously for a similar {Cr_7_Co} species containing pivalate bridging ligands,^[24]^ while the methylene protons are diastereotopic giving rise to 16 overlapping signals each with a relative integral of 2. The ^19^F NMR spectrum of the complex was attempted, however the signal(s) were broadened beyond detection likely due to short contacts to the paramagnetic transition metal ions.

**Figure 3:**
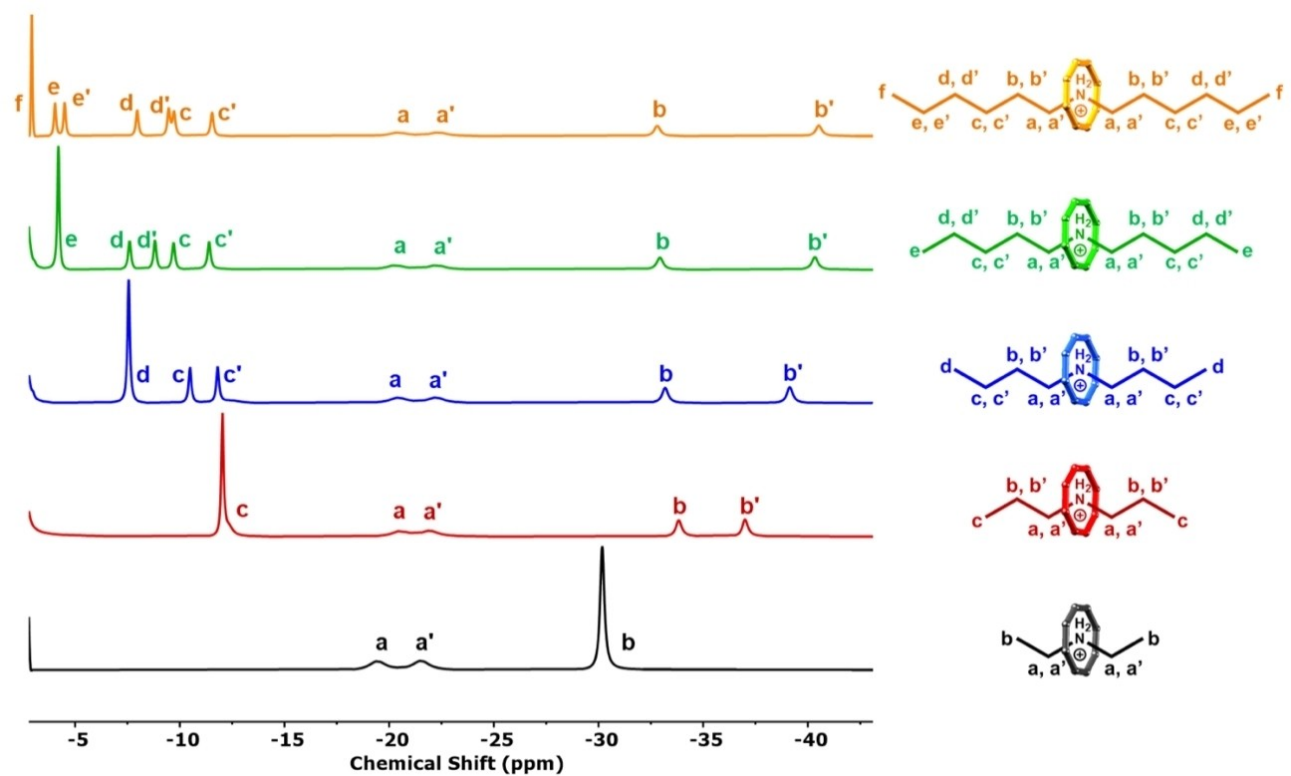
^1^H NMR (500 MHz, 298 K, 30 mM) baseline corrected spectrum (−2.5 to −45 ppm) of **Et_2_NH_2_** **⋅ 1** (black), **Pr_2_NH_2_** **⋅ 1** (red), **Bu_2_NH_2_** **⋅ 1** (blue), **Pe_2_NH_2_** **⋅ 1** (green) and **Hx_2_NH_2_** **⋅ 1** (orange) in toluene‐*d*_8_. Assigned structures of each molecule have been included for clarity.

Pseudo‐rotaxanes analogous to **Et_2_NH_2_** **⋅ 1** were synthesised with dipropyl‐, dibutyl‐, dipentyl‐ and dihexyl‐ammonium threads, giving **Pr_2_NH_2_** **⋅ 1**, **Bu_2_NH_2_** **⋅ 1, Pe_2_NH_2_** **⋅ 1** and **Hx_2_NH_2_** **⋅ 1** respectively. The upfield region (−2.5 to −45 ppm) of each spectrum is shown in Figure 3. Upon extension of each secondary ammonium carbon chain by a single methylene unit, assignment of each set of proton signals can be easily deduced from the appearance of new signals and the corresponding relative integrals. It is noticeable that the splitting of the signals from the protons on the β‐carbons is greater for the longer chains. This is probably due to hindered rotation of the secondary ammonium cations in the cavity.

### Thread Exchange

The pseudorotaxanes studied here do not contain a mechanical bond and therefore may disassemble in solution. It has been shown that the pseudorotaxanes can exchange templating ammonium ions when exposed to another potential guest.^[24]^ Deuteration studies have shown that the pivalate analogues of {Cr_7_Co} rings do not exchange carboxylates until heated to 140 °C suggesting that any macrocycle disassembly is likely to occur *via* a slipping mechanism as opposed to opening of the ring.^[15]^ Hence, at room temperature in solution, the {Cr_7_Co} ring must be able to slip off the thread in the presence of another amine in order to form a new pseudorotaxane after a proton transfer step.

### Thermodynamics Studied by NMR Spectroscopy

**Figure 4:**
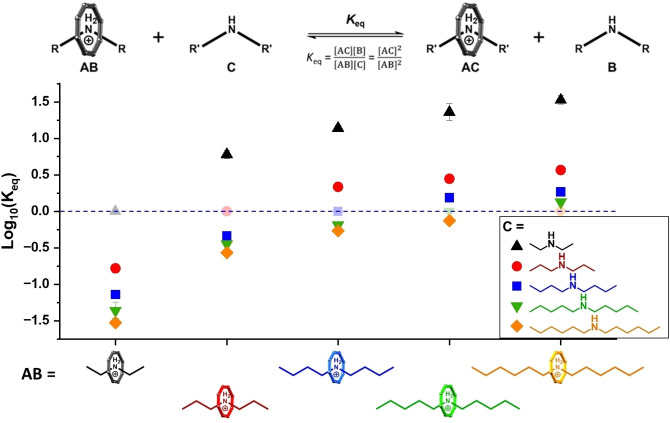
Thermodynamic plot: log $K_{eq}$ *vs*. initial pseudorotaxane species (AB) in toluene‐*d*_8_ with corresponding error bars. Log $K_{eq}$ for the equilibrium with the different product pseudorotaxanes (AC) is indicated by: black triangles (**Et_2_NH_2_** **⋅ 1**), red circles (**Pr_2_NH_2_** **⋅ 1**), blue squares (**Bu_2_NH_2_** **⋅ 1**), green triangles (**Pe_2_NH_2_** **⋅ 1**) and orange diamonds (**Hx_2_NH_2_** **⋅ 1**). Error bars are shown and are smaller than the symbol size. NMR experiments conducted at 298 K with [AB]_0_ and [C]_0_=30 mM.

^1^H NMR spectroscopy was used to investigate the position of equilibrium in such exchange interactions across the series of linear secondary ammonium ions displayed in Figure 3. As the pseudorotaxane thread protons are well separated from the rest of the spectrum, the integrals of these well‐defined signals can easily be compared. By mixing a pseudorotaxane species with an equal quantity of a competing amine, the thermodynamic constant *K*_eq_ can be estimated by monitoring the reaction until equilibrium has been reached. We never observe any intermediate species in the NMR and assume the concentration of intermediate(s) is close to zero. Therefore, the concentrations of each pseudorotaxane in the mixture can be measured from the corresponding thread integrals. A single equilibrium constant is given by the ratio of the squares of [AC]_eq_ and [AB]_eq_ (Figure 4). The reverse reactions were also completed in each case to ensure the position of equilibrium was independent of reaction direction. The equilibria were measured in toluene‐*d*_8_ and in acetone‐*d*_6_.

Integration of signals in paramagnetic NMR is not as reliable as in diamagnetic samples, but here the relative integrals of the thread proton signals are of the same order. To ensure the integrals being used are reproducible, each reaction was repeated twice in each direction and three ^1^H NMR spectra were recorded for each reaction at three time points to ensure that equilibrium was achieved. As we are implementing a user‐defined manual baseline correction, each spectrum was corrected three times with different baseline functions. This gives thirty‐six ^1^H NMR spectra to determine a standard deviation for each data point. An alternative approach to error analysis gives similar results (see SI).

The comparison of the two solvents is immediately instructive. The rate at which equilibrium was achieved in acetone‐*d*_6_ was much faster (<1 h) than in toluene‐*d*_8_ (≈400 h) at room temperature. The faster reaction in a polar solvent implies a lower activation energy in this solvent, and suggests a polar transition state.

**Figure 5:**
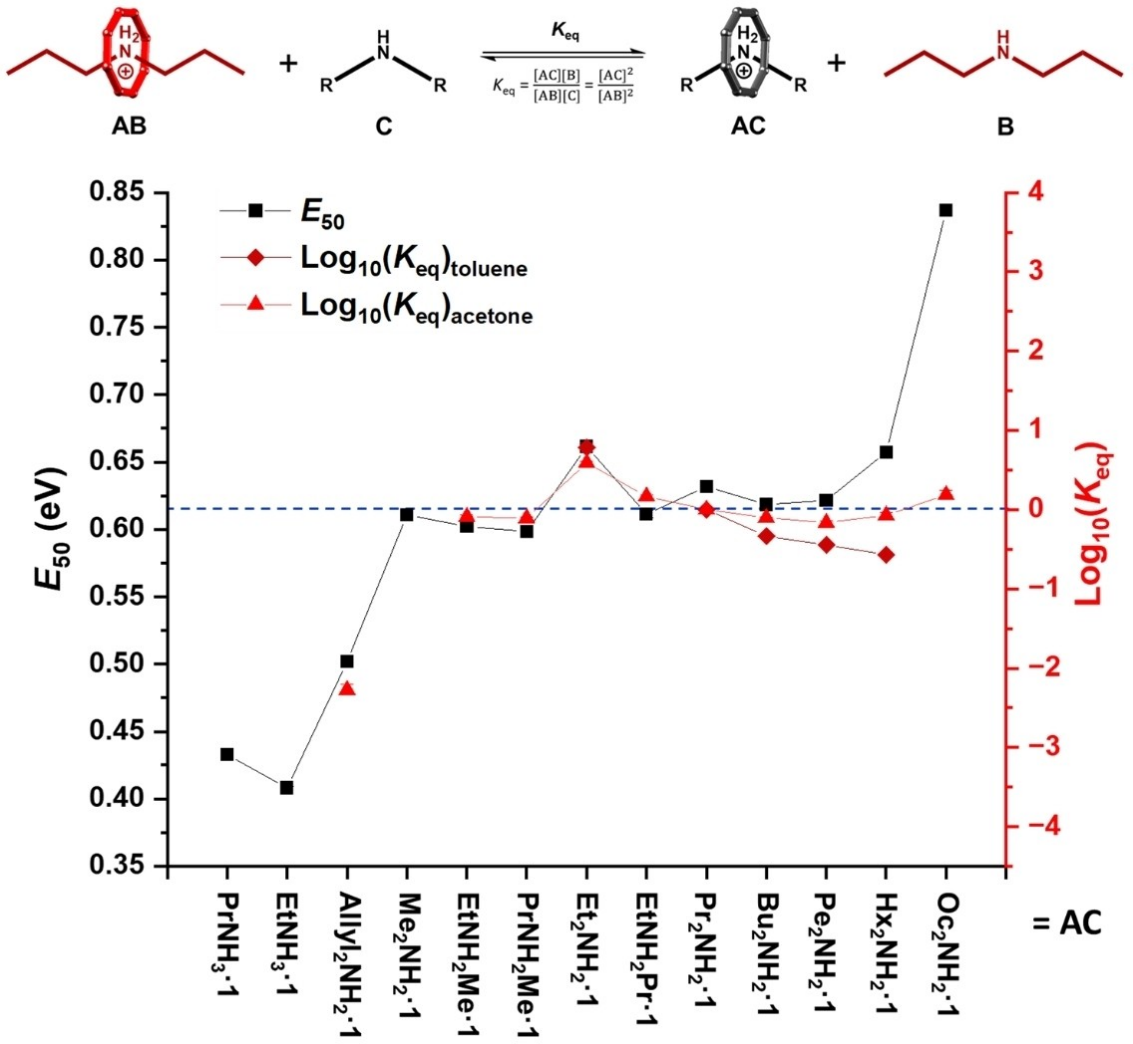
Dual axis plot showing: *E_50_* values for the dissociation of the ammonium cations from {Cr_7_Co} rings (black squares) measured using CID‐MS; and log $K_{eq}$ vs final pseudorotaxane species (AC) measured using NMR for exchange reactions in acetone‐*d*_6_ (red triangles) and toluene‐*d*_8_ (red diamonds), where AB=**Pr_2_NH_2_** **⋅ 1**. Error bars are shown and are smaller than the symbol size. NMR experiments conducted at 298 K with [AB]_0_ and [C]_0_=30 mM.

In toluene‐*d*_8_ the stability of the pseudorotaxane decreases across the series, **Et_2_NH_2_** **⋅ 1**>**Pr_2_NH_2_** **⋅ 1**>**Bu_2_NH_2_** **⋅ 1**>**Pe_2_NH_2_** **⋅ 1>Hx_2_NH_2_** **⋅ 1** (Figure 4). In acetone‐*d*_6_ there is less variation in the stability, with the order **Et_2_NH_2_** **⋅ 1** > **Pr_2_NH_2_** **⋅ 1** ≈ **Bu_2_NH_2_** **⋅ 1** ≈ **Pe_2_NH_2_** **⋅ 1<Hx_2_NH_2_** **⋅ 1** (Figure 5). The trend observed in toluene‐*d*_8_ may reflect a difference in solvation of the ‘free amine’ which is not observed to the same extent in acetone‐*d*_6_. Propane has been reported to have a higher solubility in toluene than ethane, which weakly supports this interpretation.^[25]^ In both toluene‐*d*_8_ and acetone‐*d*_6_, pseudorotaxane **Et_2_NH_2_** **⋅ 1** has a relatively high stability compared to those with longer chain secondary ammonium threads. It is not obvious why the diethylammonium should bind most strongly as the guest in the heterometallic host.

As equilibria were more quickly reached in acetone‐*d*_6_ and amine solvation was an important factor in toluene‐*d*_8_ further experiments were performed in acetone only. We began with **1** templated with a primary ammonium cation, propylammonium giving **PrNH_3_** **⋅ 1**. Reactions between **PrNH_3_** **⋅ 1** and Pr_2_NH rapidly proceeded to completion to give **Pr_2_NH_2_** **⋅ 1**. A similar result was seen beginning with ethylammonium as the guest cation. Therefore, secondary ammonium cations appear to be more favourable stations for the {Cr_7_Co} ring **1** than primary ammoniums. This is the opposite trend to that observed with crown ethers; with crown ethers larger association constants are seen for primary alkylammonium than secondary or tertiary ammonium cations (RNH_3_^+^ > R_2_NH_2_^+^ > R_3_NH^+^) due to the larger number of hydrogen bond donors.^[26]^ From the predicted proton positions in the crystal structures of **PrNH_3_** **⋅ 1** and **Pr_2_NH_2_** **⋅ 1**, increasing the number of NH protons from two to three does not introduce any further N−H ⋅ ⋅ ⋅ F hydrogen short contacts (<3 Å, Figure S39). The size and rigidity of the {Cr_7_Co} cavity is therefore likely changing the stability trend for **1** when compared to crown ethers.

**Figure 6:**
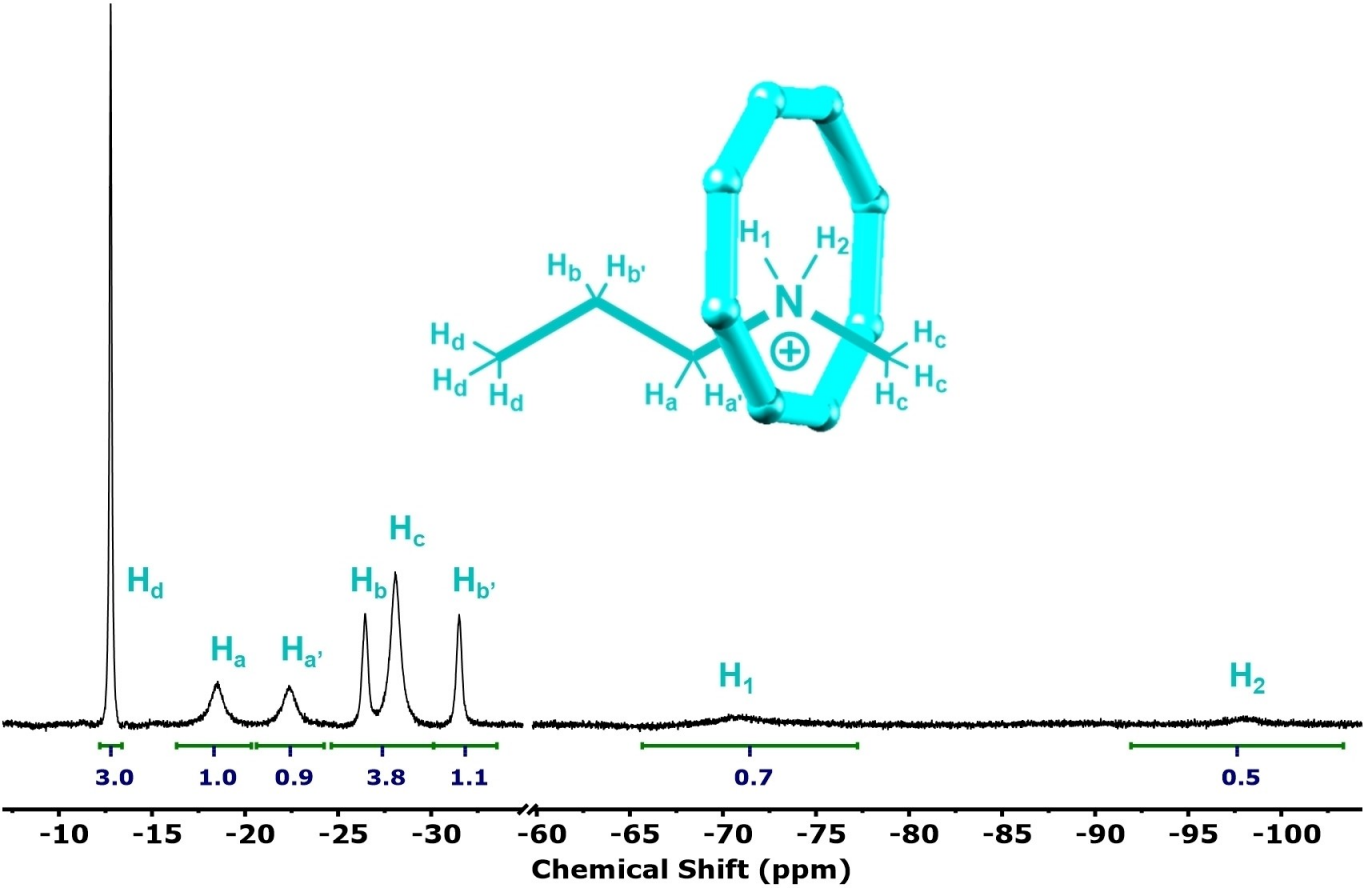
^1^H NMR (500 MHz, 298 K, 30 mM) baseline corrected spectrum of **PrNH_2_Me ⋅ 1** in acetone‐d_6_.

The low stability of PrNH_3_^+^ as a guest provides an indirect synthesis pathway for other pseudorotaxanes in higher yields than would be expected from a template synthesis. To demonstrate this potential we performed exchange reactions with asymmetric linear threads. Exchange of ethylpropylamine, methylpropylamine and ethylmethylamine with **PrNH_3_** **⋅ 1** gave pseudorotaxanes **EtNH_2_Pr ⋅ 1**, **MeNH_2_Pr ⋅ 1** and **EtNH_2_Me ⋅ 1** in high yields (80 ‐ 90 %, see SI). The introduction of an non‐symmetric thread into the {Cr_7_Co} ring results in a lowering of symmetry which is reflected in the increased number of signals in the ^1^H NMR spectrum (S12 ‐ 14). Figure 6 displays the ^1^H NMR spectrum of **MeNH_2_Pr ⋅ 1** highlighting the effect of a reduction in symmetry. In each of the three ^1^H NMR spectra, NH_2_ protons become diastereotopic leading to two broad, signals at −70 and −97 ppm. The carboxylate methyl groups are split in to 16 signals each with a relative integral of 9, whereas the downfield methylene protons give 32 overlapping singlets each with a relative integral of 1.

Diallylamine [(CH_2_=CH−CH_2_)_2_NH] was also used to prepare pseudorotaxane **Allyl_2_NH_2_** **⋅ 1** in order to understand the involvement of potentially stabilising hydrocarbon interactions in the resultant pseudorotaxane. Figure 5 shows a log plot for each of the pseudorotaxane species in acetone‐*d*_6_. The log $K_{eq}$ values have been calculated against **Pr_2_NH_2_** **⋅ 1** as the starting pseudorotaxane (the inverse *K*_eq_ from the reverse reaction also contributes to the data in Figure 5). The general trend shows how in acetone‐*d*_6_ most of the pseudorotaxanes with saturated dialkylammonium threads have a similar stability in solution to **Pr_2_NH_2_** **⋅ 1** and therefore give an equilibrium constant close to 1 (log $K_{eq}$ ≈0). The exception is **Et_2_NH_2_** **⋅ 1** which has a larger log $K_{eq}$ (0.589±0.0260).

**Allyl_2_NH_2_** **⋅ 1** is shown to undergo almost complete thread exchange when in the presence of dipropylamine in acetone‐*d*_6_ ($K_{eq}$ =188, i. e. log $K_{eq}$ =−2.27 for the reverse reaction in Figure 5). An attempt to make a pseudorotaxane using dipropargylamine [(CH≡C−CH_2_)_2_NH] as template failed, both *via* a direct route or by exchange. The results with diallylamine and dipropargylamine indicates a preference for saturated alkyl chains within the amines. The log $K_{eq}$ values associated with the exchange of primary alkylammoniums with **Pr_2_NH_2_** **⋅ 1** have not been included as the reactions proceeded almost to completion meaning that quantification of the relative concentrations of **EtNH_2_** **⋅ 1** and **PrNH_2_** **⋅ 1** could not be accurately determined using ^1^H NMR. Exchange of **Pr_2_NH_2_** **⋅ 1** with Me_2_NH was not carried out as Me_2_NH is a gas at 298 K.

### Collision‐Induced Dissociation Mass Spectrometry (CID‐MS)

A complementary technique to assess the stability of the pseudorotaxanes is CID‐MS, which, being a gas‐phase measurement, offers the unique perspective of solvent molecules being absent. This allows the deconvolution of the non‐covalent interactions within the pseudorotaxane from those occurring with the solvent.^[27]^ In CID‐MS, target ions are *m/z*‐isolated and subsequently activated *via* collisions with an inert gas (here nitrogen), which leads to the dissociation of the precursor ion. The number and types of fragments depend on the applied, user‐defined kinetic energy with which the ion is accelerated into the collision cell. For different centre‐of‐mass collision energies *E_com_* (see Supporting Information), the relative intensity of the precursor ion (“survival yield”) can be determined, and the *E_com_* at which the precursor ion decay reaches 50 % (“*E_50_*”) is a relative measure for ion stability.[^28^, ^29^, ^30^] We have previously used *E*_50_ values to assess the relative stability of similar homometallic rings and rotaxanes of the type {Cr_7_M} (M−Cr^III^, Mn^II^, Fe^II^, Co^II^, Ni^II^, Cu^II^, Zn^II^ and Cd^II^), as well as {Cr_x_Cu_2_} (x=10, 12) hourglass structures and a bi‐cyclic {Cr_12_Gd_4_} complex.[^19^, ^31^, ^32^]

In CID‐MS studies of the {Cr_7_Co} pseudorotaxanes show a similar disassembly pathway to that previously observed,^[31]^ involving loss of the secondary ammonium cation and a carboxylate ligand as the main channel (see Figure S40). Hence, the *E*_50_ values are comparable to the exchange studied by NMR and are displayed in Figure 5. The *E*_50_ values are significantly lower for primary ammonium than for dialkylammonium threads. The *E*_50_ for **Allyl_2_NH_2_** **⋅ 1** is significantly lower than the saturated analogue **Pr_2_NH_2_** **⋅ 1**. Both these observations are consistent with the thread exchange reactions in acetone‐*d*_6_ solution (see above). Most of the pseudorotaxanes with dialkylammonium threads have an *E_50_* value close to 0.6 eV, the exception being **Et_2_NH_2_** **⋅ 1** (0.661±0.00140 eV) which is a little larger than for **Pr_2_NH_2_** **⋅ 1**, **Bu_2_NH_2_** **⋅ 1** or **Pn_2_NH_2_** **⋅ 1**. This suggests that the {Cr_7_Co} ring binds more strongly to diethylammonium. A direct comparison of the *E_50_* values with the log$\;K_{eq}$ measured in acetone‐*d*_6_ and toluene‐*d*_8_ is shown in Figure 5. This shows a good correlation for most amines studied with the exception of the longest chains. The *E_50_* values for **Hx_2_NH_2_** **⋅ 1** and **Oc_2_NH_2_** **⋅ 1** are noticeably higher which may arise due to the reduced number of direct gas phase collisions capable of removing the thread.

### Kinetics

**Figure 7:**
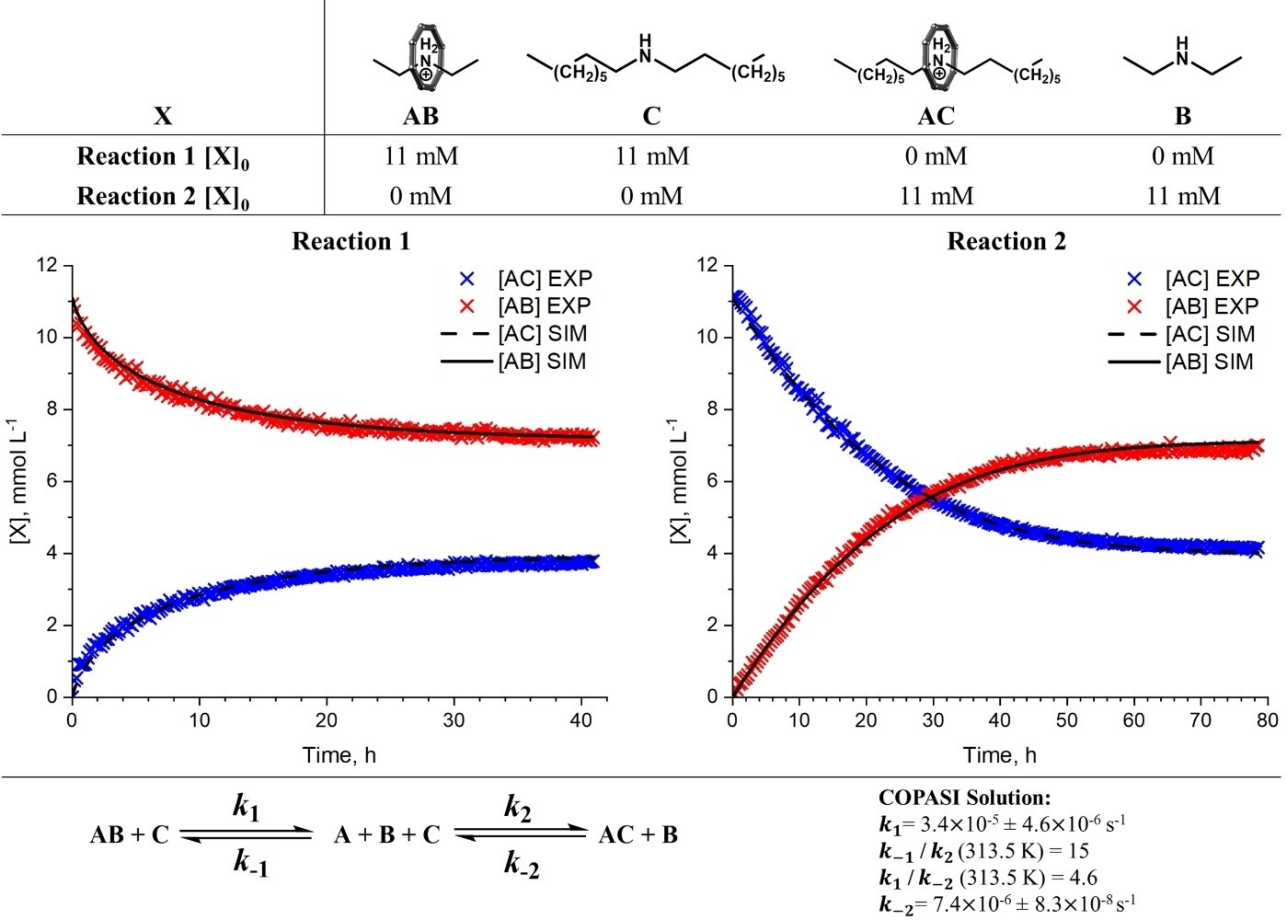
Kinetic studies of a thread exchange reaction. Initial concentrations ([X]_0_) used to set‐up Reactions 1 and 2 (top). Kinetic plots for thread exchange reactions between pseudorotaxanes **Et_2_NH_2_** **⋅ 1** and **Oc_2_NH_2_** **⋅ 1** in acetone‐*d*_6_ at 313.15 K (from 700 MHz ^1^H NMR), where: red crosses=[AB]^EXP^, blue crosses=[AC]^EXP^, black solid line=[AB]^SIM^, black dash line=[AC]^SIM^ (middle). Proposed dissociative mechanism and estimated rate constant parameters from COPASI simulations (bottom). Errors are displayed as 3 × standard deviation from the parameter estimation.

To establish a possible mechanism for the exchange reaction, the kinetics of a single exchange reaction was studied. The reaction involving exchange between the pseudorotaxanes **Et_2_NH_2_** **⋅ 1** and **Oc_2_NH_2_** **⋅ 1** in acetone‐*d*_6_ was slow enough to be investigated using ^1^H NMR (700 MHz, 313.15 K). The reaction was studied in both directions. Reaction 1 began with **Et_2_NH_2_** **⋅ 1** and Oc_2_NH, while reaction 2 began with **Oc_2_NH_2_** **⋅ 1** and Et_2_NH. The reactions were monitored by mixing 0.01 mmol of the starting pseudorotaxane with an equal quantity of competing amine (Figure 7). The concentrations of **Et_2_NH_2_** **⋅ 1** and **Oc_2_NH_2_** **⋅ 1** were calculated by comparing the spectral integrals of characteristic thread signals (*ca*. −25.8 and −10.1 ppm, respectively; see Supporting Information for assignments) to the integral of a dioxane singlet of a known concentration. The calculated values of [AB]_0_ and [AC]_0_ indicate that no dissociation occurs in the absence of a competing amine supporting the assumption that no intermediate species is observed.

Spectra were collected continuously over the course of 40 h (Reaction 1; spectrum acquisition time 10 m) or 80 h (Reaction 2; acquisition time 20 minutes) (Figure 7). The data from the two reactions were fit simultaneously using COPASI^[33]^ for both associative and dissociative mechanisms, to determine the rate constants *k*_1_, *k*_‐1_, *k*_2_ and *k*_‐2_ (at 313.15 K) as defined in Figure 7.

**Figure 8:**
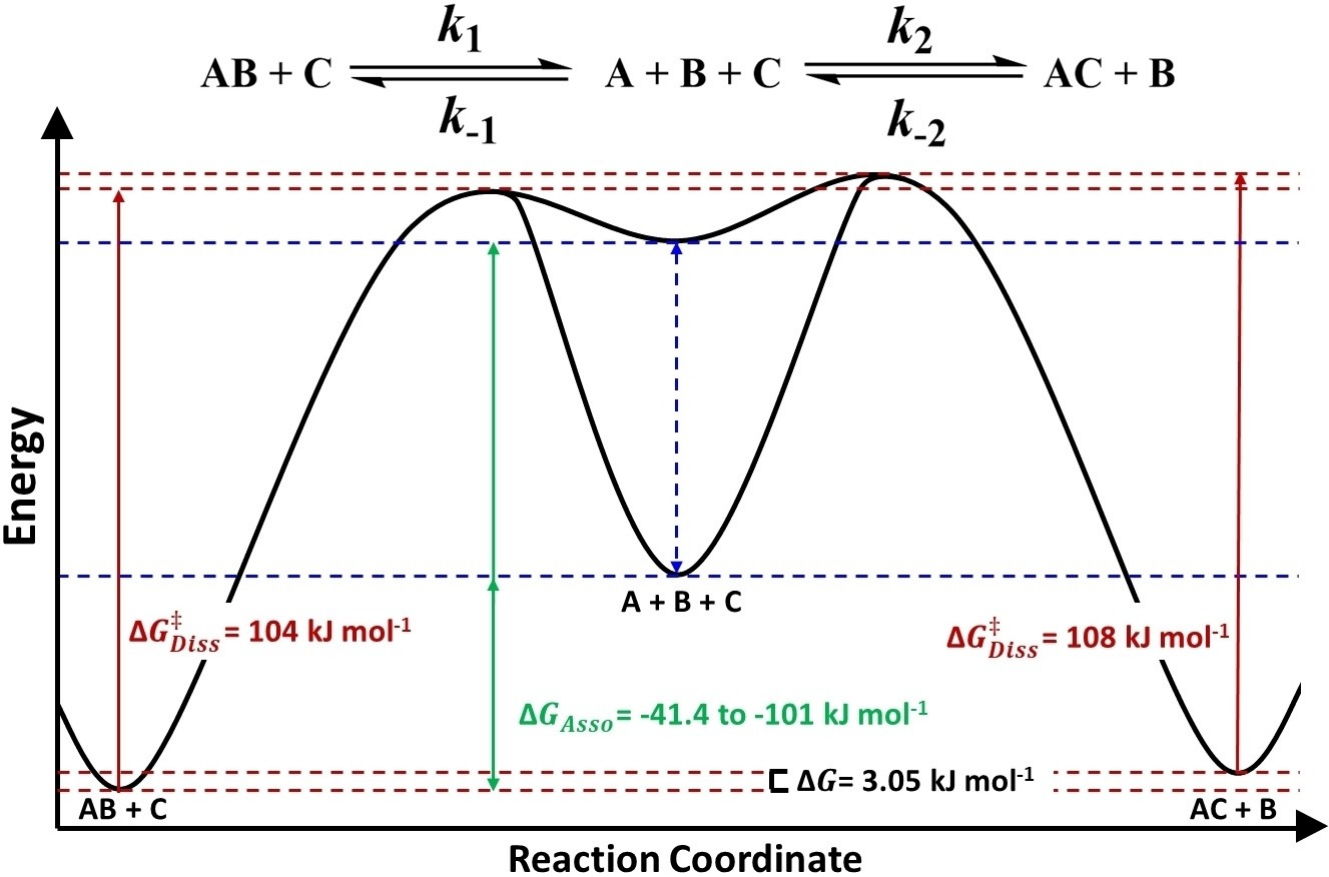
Crude potential energy diagram for the amine exchange in {Cr_7_Co} pseudorotaxanes. Dissociation barriers for **Et_2_NH_2_** **⋅ 1** (AB) and **Oc_2_NH_2_** **⋅ 1** (AC) are shown in red. The association rate constants are less well‐defined (see text) and we show the estimated range for Δ$G_{Asso}$ in green. Calculated values of Δ$G_{Diss}^{\neq }$ and Δ$G_{Asso}^{\neq }$ are obtained using the Eyring equation Δ$G_{Diss/Asso}^{\neq }$ =−RT ln[*k*_x_ *h/kb T*] where *k*_x_=*k*_1_, *k*_‐1_, *k*_2_ or *k*_‐2_ (313.5 K). Δ$G_{Asso}$ was calculated using the equation: ΔG=-RT ln(*K*_eq_), Where: *K*_eq_=*k*_‐1_/*k*_1_ or *k*_2_/*k*_‐2_ (313.5 K). ΔG was calculated using the same equation where $K_{eq}$ =(*k*_1_/*k*_‐1_ × *k*_2_/*k*_‐2_)=${\left[AC\right]}_{eq}^{2}$ /${\left[AB\right]}_{eq}^{2}$ .

The simulation for a dissociative mechanism gave a good fit (Figure 7, objective function value=7.56) whereas the associative mechanism gave a poor fit (objective function value=191) to the experimental data (see Supporting information). Parameter estimations for a dissociative mechanism were repeated ten times by randomising the start values of each parameter and alternating the optimisation method (see Supporting information). In each case, the values of $k_{1}$ and $k_{-2}$ remained constant (*ca*. 3.4× 10^−5^ s^−1^ and 7.4× 10^−6^ s^−1^, respectively). Whilst the ratio of $k_{-1}$ /$k_{2}$ optimises to the same values (*ca*. 14.8) between repeated optimisations, multiple possible solutions for association constants $k_{-1}\;$ and $k_{2}$ are found. The magnitude of the fitted parameters of $k_{-1}\;$ and $k_{2}$ vary considerably (2.71× 10^2^–2.17× 10^12^ and 182–1.48× 10^11^ L mol^−1^ s^−1^, respectively), but in each case are many orders of magnitude larger than dissociation constants $k_{1}\;$ and $k_{-2}$ . This crude calculation is consistent with the total concentration of pseudorotaxane remaining constant and close to [AB]_0_ throughout the course of the exchange reaction (see Supporting information), suggesting a high energy intermediate (A) (Figure 8). It has been proposed that the proton being exchanged between pseudorotaxanes remains within the cavity of the {Cr_7_Co} ring to give the neutral intermediate species A.^[24]^ The wide range of possible values for the association constants result in a range of Gibbs free energies of amine association, Δ$G_{Asso}$ between −38.3 and −101 kJ mol^−1^ (Figure 8).

Comparing $k_{1}\;$ and $k_{-2}$ , dissociation of **Et_2_NH_2_** **⋅ 1** was found to be *ca*. 4.6 times faster than that of **Oc_2_NH_2_** **⋅ 1**; this can be attributed to the shorter thread length increasing the probability of a de‐threading event. This is also consistent with the CID‐MS studies, where a larger *E*_50_ is found for **Oc_2_NH_2_** **⋅ 1**. Multiplication of the sequential equilibrium constants give a net equilibrium constant $K_{eq}$ =0.32 (ΔG =+3.05 kJ mol^−1^). Through use of the Eyring equation, rate constants $k_{1}$ and $k_{-2}$ give the Gibbs free enthalpy of dissociation Δ$G_{Diss}^{\neq }$ =103.6±0.7 kJ mol^−1^ and 107.57±0.06 kJ mol^−1^, respectively.

### Understanding Relative Stabilities

**Figure 9:**
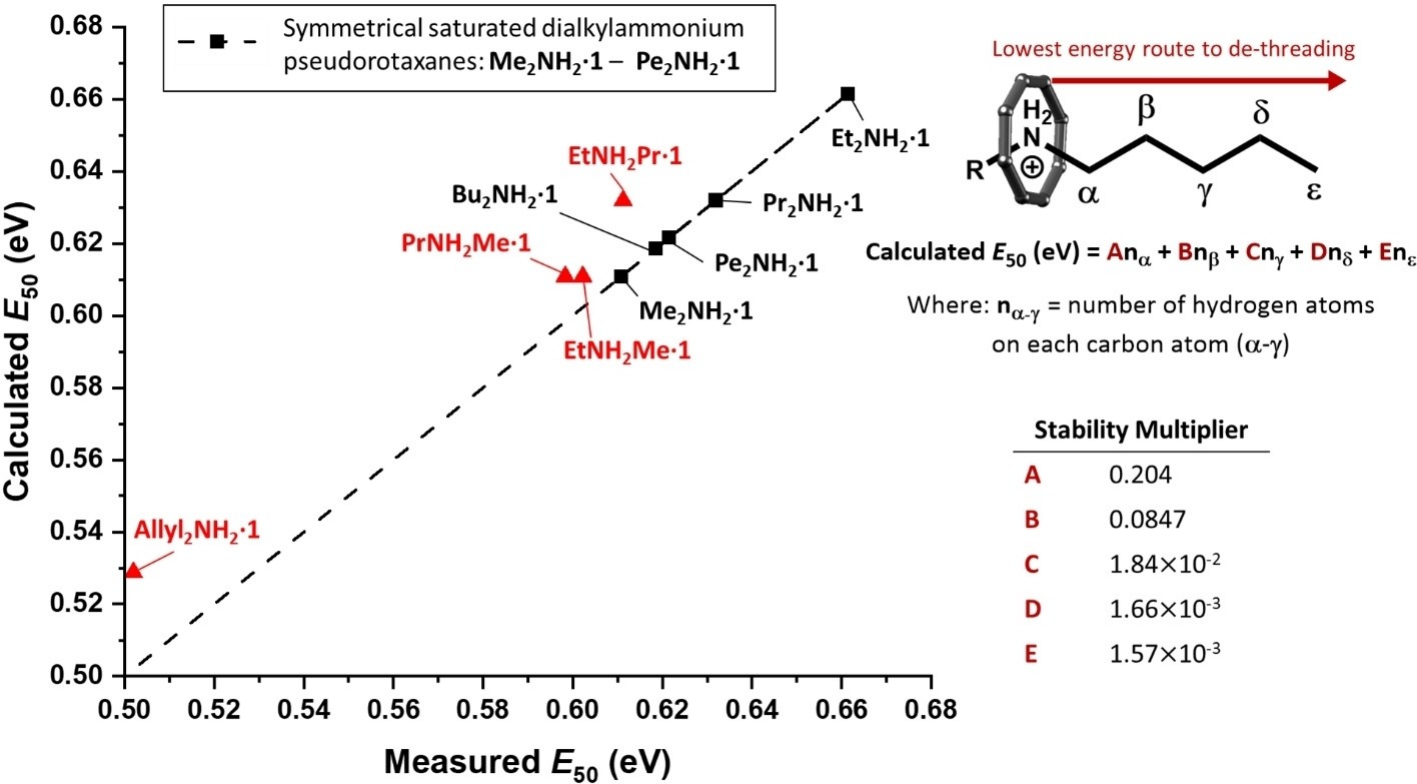
Fit of experimental *E*_50_ values for saturated symmetrical dialkylammonium pseudorotaxanes (black squares) against calculated values simultaneously fitted to equation [1] with best fit parameters **A**–**E** shown in the inset. Data points in red are for the asymmetric pseudorotaxanes **Allyl_2_NH_2_** **⋅ 1**, **PrNH_2_Me ⋅ 1**, **EtNH_2_Me ⋅ 1** and **EtNH_2_Pr ⋅ 1** using the fixed values of **A**–**E** from the inset. This gives us a dethreading order: Me<Pr<Et.

In an attempt to investigate the contribution of alkyl‐chain hydrogen atoms to the stabilities of each pseudorotaxane, an equation containing five unknown stability multipliers (**A**–**E**) was solved using the measured *E*_50_ values derived from CID‐MS for the symmetric ammonium cations **Me_2_NH_2_** **⋅ 1**, **Et_2_NH_2_** **⋅ 1**, **Pr_2_NH_2_** **⋅ 1**, **Bu_2_NH_2_** **⋅ 1** and **Pe_2_NH_2_** **⋅ 1**. The number of hydrogen atoms ($n_{x}$ ) found at carbon atoms α-γ are multiplied by **A**–**E**, respectively, and the results summed (Figure 9) (equation [1]). Thus for **Pr_2_NH_2_**.**1**, $n_{\alpha }$ =2, $n_{\beta }$ =2, $n_{\gamma }$ =3.

(1)

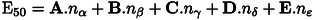




An excellent fit to all five compounds is found with this model. Stability multiplier **A** is found to be largest, suggesting that α hydrogens contribute most significantly to the stability of the pseudorotaxanes. **B** is the second largest multiplier and roughly half the magnitude of **A**. Multipliers **C**, **D** and **E** are much smaller in magnitude. This observation could suggest that the further the alkyl‐chain hydrogen atoms are from the central nitrogen, the fewer stabilising interactions are available with the {Cr_7_Co} ring. **Allyl_2_NH_2_** **⋅ 1** and asymmetric pseudorotaxanes (**PrNH_2_Me ⋅ 1**, **EtNH_2_Me ⋅ 1** and **EtNH_2_Pr ⋅ 1**) are also shown in Figure 9.

The calculated *E*_50_ values for pseudorotaxanes containing asymmetric threads assumed a lowest energy route to de‐threading and used the best fit **A**–**E** parameters derived from the symmetric threads. Hence, the calculated *E_50_* for **EtNH_2_Pr ⋅ 1** matches that of **Pr_2_NH_2_** **⋅ 1** while both **PrNH_2_Me ⋅ 1** and **EtNH_2_Me ⋅ 1** match **Me_2_NH_2_** **⋅ 1**.

**Allyl_2_NH_2_** **⋅ 1** has a much smaller calculated *E*_50_ value compared to the saturated equivalent **Pr_2_NH_2_** **⋅ 1** due to the reduced number of β hydrogens. The absence of any β hydrogens in dipropargylamine would give the resulting pseudorotaxane a calculated *E*_50_ value close to that of pseudorotaxanes containing primary ammonium threads (0.426 eV). This may offer a reason as to why an amine exchange synthesis of this compound was unsuccessful.

The observation that protons on the α‐ and β‐carbon contribute to the stability of the pseudorotaxane is clear; the explanation is less obvious. The crystal structures are all disordered, and this must be borne in mind when short contacts are considered as evidence for weak interactions. Taking **Pr_2_NH_2_** **⋅ 1** as a representative case: we considered whether the H‐atoms on the α‐, β‐ and γ‐carbons could make short contacts to electronegative atoms, producing weak C−H ⋅ ⋅ ⋅ X hydrogen bonds.^[34]^ We discuss the C….X distances below, without making assumptions of the positions of the H‐atoms.

For the major component of the structure of **Pr_2_NH_2_** **⋅ 1**, the eight α‐C ⋅ ⋅ ⋅ F contacts fall in the range 3.32–3.86 Å, with the shortest α‐C ⋅ ⋅ ⋅ O contact around 4.15 Å. The eight β‐C ⋅ ⋅ ⋅ F contacts fall in the range 3.54–4.91 Å, with two contacts of 3.54 and 3.65 Å; there are three short β‐C ⋅ ⋅ ⋅ O contacts around 4 Å: 3.91, 4.05 and 4.07 Å. For the γ‐C, the shortest γ‐C ⋅ ⋅ ⋅ F contact is 4.52 Å and γ‐C ⋅ ⋅ ⋅ O contact is 4.48 Å. There is clearly no C−H ⋅ ⋅ ⋅ X hydrogen bond for the γ‐C carbons, but for the α‐C and β‐C weak C−H ⋅ ⋅ ⋅ X bonds are possible. The larger number of C−H ⋅ ⋅ ⋅ F for the α‐C could explain why the α‐protons make the largest contribution to stability. The lack of any potential C−H ⋅ ⋅ ⋅ X hydrogen bonds for the γ‐, δ‐ or ϵ‐protons explains why they have almost no contribution to the stability.

### Comparison with Crown Ethers

Association of a dibenzo[24]crown‐8 (DB24C8) macrocyle to a series of disubstituted dibenzylammonium cations has prevously been studied using UV‐vis and ^1^H NMR.[^3^, ^4^, ^5^, ^6^, ^7^, ^8^, ^9^, ^10^, ^11^, ^12^, ^13^, ^14^, ^15^, ^16^, ^17^, ^18^, ^19^, ^20^, ^21^, ^22^, ^23^, ^24^, ^25^, ^26^, ^27^, ^28^, ^29^, ^30^, ^31^, ^32^, ^33^, ^34^, ^35^, ^36^, ^37^, ^38^] The calculated Gibbs free energy of association for the organic [2]‐rotaxanes vary between ca. −12 and −34 kJ mol^−1^ (Table 2) and is smaller than the range of Δ$G_{Asso}$ values predicted here. As the {Cr_7_Co} rings have a sizable preference to associate with ammonium ions, release of an amine thread is avoided. The Δ$G_{Diss}^{\neq }$ calculated for **Et_2_NH_2_** **⋅ 1** and **Oc_2_NH_2_** **⋅ 1** is of a similar order to those measured for rotaxanes comprising the DB24C8 macrocycle (Table 2). Unlike **Et_2_NH_2_** **⋅ 1** and **Oc_2_NH_2_** **⋅ 1**, threads displayed in Table 2 contain large stopper groups to increase the stability of the resultant rotaxane molecules. Therefore, comparison suggests that the {Cr_7_Co} pseudorotaxanes have ‘rotaxane like’ character in solution due to the relatively large Gibbs free enthalpy of dissociation (due to electrostatic stabilisation). Importantly, a {Cr_7_Co} intermediate species, A, provides a pathway to amine exchange and therefore potential shuttling between stations along a single thread. These {Cr_7_Co} rings present different host behaviour to more conventional crown ethers.

**Table 2:**
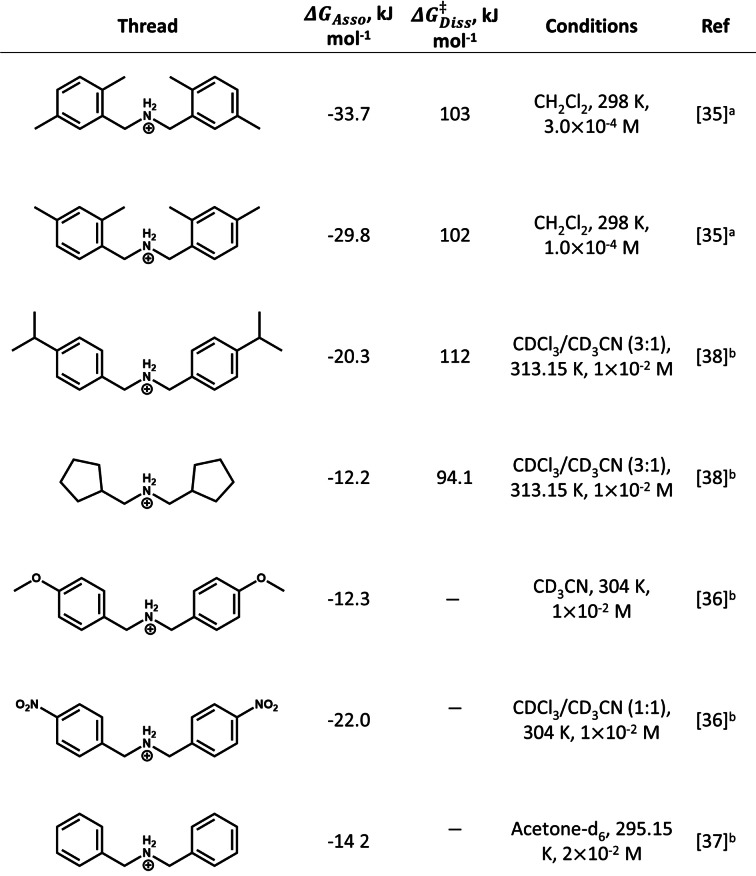
Experimental kinetic constants for the dissociation of cationic threads from DB24C8.[^35^, ^36^, ^37^, ^38^]

[a] Kinetic data measured using UV‐Vis experiments. [b] Kinetic data measured using ^1^H NMR experiments.

## Conclusions

We have shown that paramagnetic ^1^H NMR spectroscopy can be used to investigate the mechanism of pseudorotaxane thread exchange. Likewise, the relative stability of {Cr_7_Co} pseudorotaxanes based on the design of the thread and solvent can be probed using ^1^H NMR spectroscopy. The equilibration occurs more quickly in acetone‐*d*_6_ than toluene‐*d*_8_. Pseudorotaxanes containing primary amines have been shown to exchange to completion when exposed to an equal quantity of dipropylamine in solution and are similarly unstable when studied with CID‐MS. Knowledge of this alternate pathway to synthesis has been used to synthesise a series of pseudorotaxanes containing asymmetric ammonium threads. The results here are the first step towards using these inorganic rings in molecular shuttles and ratchets, similar to the work on molecular machines by Stoddart, Leigh and others. We now understand which ammonium cations can be used as “leaving groups” and which solvent systems will allow these reactions to proceed. The very different binding properties compared with crown ethers should also allow us to make rotaxanes containing two distinct rings.

## Supporting Information

The authors have cited additional references within the Supporting Information.[^39^, ^40^, ^41^, ^42^] (Methods (page 2). Preparation and physical characterization data (pages 4–8). ^1^H NMR assignments (S1–S15). Thread exchange experiments (S16–S42). Crystallography (pages 55–58). Examples of CID‐MS (S49).

CCDC  2312754–231758 contains the supplementary crystallographic data for this paper. These data can be obtained free of charge *via* www.ccdc.cam.ac.uk/conts/retrieving.html (or from the Cambridge Crystallographic Data Centre, 12 Union Road, Cambridge CB21EZ, UK; fax: (+44)1223‐336‐033; or deposit@ccdc.cam.ac.uk).

Supplementary data sets containing the measured NMR data and the COPASI parameter estimation results are available on Figshare as data sets https://doi.org/10.48420/24771711; https://doi.org/10.48420/24771705; https://doi.org/10.48420/24771678; https://doi.org/10.48420/24771570; https://doi.org/10.48420/24771522; https://doi.org/10.48420/24771504.

## Conflict of interests

The authors declare no conflict of interest.

1

## Supporting information

As a service to our authors and readers, this journal provides supporting information supplied by the authors. Such materials are peer reviewed and may be re‐organized for online delivery, but are not copy‐edited or typeset. Technical support issues arising from supporting information (other than missing files) should be addressed to the authors.

Supporting Information

## Data Availability

The data that support the findings of this study are openly available in FIGSHARE at https://figshare.manchester.ac.uk/articles/dataset/Toluene NMR data/24771711, reference number 24771711.
