# Supplementary material for: Studying Cation Exchange in {Cr7Co} Pseudorotaxanes: Preparatory Studies for Making Hybrid Molecular Machines
Source: Chemistry. 2024 Jun 11;30(37):e202400432. doi: 10.1002/chem.202400432 (PMC11497308; doi:10.1002/chem.202400432)
Supplement: Supplementary file 1 — Supporting Information [file CHEM-30-e202400432-s001.pdf]

Chemistry–A European Journal

Supporting Information

Studying Cation Exchange in $\{Cr_7Co\}$ Pseudorotaxanes: Preparatory Studies for Making Hybrid Molecular Machines

Tom S. Bennett, Niklas Geue, Grigore A. Timco, George F. S. Whitehead, Inigo J. Vitorica-Yrezabal, Perdita E. Barran, Eric J. L. McInnes, and Richard E. P. Winpenny*

Supporting Information

Studying Cation Exchange in {Cr_7Co} Pseudorotaxanes Using ^1H NMR Spectroscopy: Preparatory Studies for Making Hybrid Molecular Machines

Tom S. Bennett[1], Niklas Geue[2], Grigore A. Timco[1], George F. S. Whitehead[1], Inigo J. Vitorica-Yrezabal[1], Perdita E. Barran[2], Eric J. L. McInnes[1] and Richard E. P. Winpenny[1]

[1]Department of Chemistry, The University of Manchester, Oxford Road, Manchester, M13 9PL, UK. [2]Michael Barber Centre for Collaborative Mass Spectrometry, Manchester Institute of Biotechnology, Department of Chemistry, The University of Manchester, 131 Princess Street, Manchester, M1 7DN, UK.

Table of Contents

1. Methods **2**

1.1 ^{1}H NMR Spectroscopy Experiments 2

1.2 Mass Spectrometry 3

2. Experimental Procedures **4**

3. ^{1}H NMR Assigned Spectra **9**

4. Thread Exchange Experiments **24**

5. Crystallography **55**

6. Examples of CID-MS **59**

7. References **60**

1. Methods

1.1 ^{1}H NMR Spectroscopy Experiments

All ^{1}H NMR experiments were conducted in the NMR facility in the department of chemistry within the University of Manchester.

All ^{1}H NMR experiments kinetic experiments were conducted using a Bruker NEO 700 spectrometer equipped with a 5mm broadband cryoprobe. Experiments were carried out using a D1 time > 5$\times T_1$ (dioxane and thread signals) to allow for quantitative spectra (128 – 256 scans).

A Bruker AVIII HD 500 – Gold –B500 spectrometer equipped with a nitrogen-cooled broadband Prodigy cryoprobe was used to carry out all thermodynamic ^{1}H NMR experiments. Experiments were carried out using a D1 time > 5$\times T_1$ (thread signals or dioxane signal) to allow for quantitative comparison of signals (128 – 256 scans).

Spin–lattice (T_1) relaxation experiments were conducted at 500 MHz, 298 K for CH_3 thread signals of **Pr_2NH_2·1** (centred at -11.7 ppm). Inversion recovery experiments gave a relaxation time of 8.13 for **Pr_2NH_2·1**. T_1 relaxation experiments were conducted at 700 MHz, 313.15 K for CH_3 or CH_2 thread signals of **Et_2NH_2·1**, **Oc_2NH_2·1** and dioxane (centred at -25.9, -10.1 and 3.61 ppm respectively). Inversion recovery experiments gave relaxation times of: **Et_2NH_2·1** = 3.92 ms, **Oc_2NH_2·1** 11.1 ms and dioxane = 673 ms.

Exchange experiments were conducted in NMR tubes containing a total volume of 0.9 mL. 5 mL stock solutions for each pseudorotaxane (20 mM), amine (50 mM) and dioxane (42.5 mM) were made using fresh bottles of either toluene-d_8 or acetone-d_6. For kinetic studies, 0.01 mmol an amine (0.2 mL from stock solution) is added to an equal quantity of pseudorotaxane (0.5 mL from stock solution) and 0.85 equivalents of dioxane (0.2 mL of the stock solution). Dioxane is used as a reference in the kinetic studies to quantify the concentration of each pseudorotaxane in solution through the course of the reaction. For thermodynamic studies, the same concentrations of stock solutions for each amine and pseudorotaxane is used alongside the addition of an equivalent of dioxane to help phase the NMR spectra. For thermodynamic ^{1}H NMR studies, experiments were conducted as above but using 30 mM initial amine and pseudorotaxane concentrations at 298 K.

Phase and baseline corrections for each ^{1}H NMR spectra were carried out using MestreNova Version 14.1.1-24571. The MestreNova manual phase correction centred on a dioxane solvent signal (≈ 3.4 ppm) has been used. This technique allows for both a zero order phase correction (centred on the reference signal) followed by a first order phase correction to correct the phase of the remaining signals. The MestreNova multipoint Baseline correction has been used on each ^{1}H NMR spectra as this method offers the most sensible baseline approximation for the paramagnetic samples. The method allows the user to select a set of points along the spectra to define the baseline. A cubic splines algorithm is used to generate smooth curves between the linked points.

In a few cases a line fitting (deconvolution) has been used for signals which overlap slightly. This method works best when two signals are only slightly overlapping. Using this method, a reasonable estimation of the contribution of each signal to the total integral of the two signals can be made. MestreNova uses a mixed function of Gaussian and Lorentzian contributions to fit the sum of the two signals. This function allows the user to match the position, peak height, width and Lorentzian/Gaussian ratio of the signal.

For the thermodynamic ^{1}H NMR study, to ensure the integrals being used are reliable and reproducible, each reaction was repeated twice in each direction where three ^{1}H NMR spectra were recorded for each reaction to ensure that equilibrium was achieved. Each spectrum was baseline corrected three times permitting 36 ^{1}H NMR spectra to contribute to a standard deviation for each data point.

COPASI[33] parameter estimations were used to fit associative and dissociate mechanisms to experimental data (Reaction 1 and Reaction 2 simultaneously) by altering rate constants k_1, k_{-1}, k_2 and k_{-2}.

Within each parameter optimisation, rate constants were allowed to vary independently between 0 and 3.6×10^{16} s^{-1} (or 0 to 2.78×10^{16} L s^{-1} mol^{-1}). The start values for each rate constant were varied and result analysed when an objective function value minima was achieved. Two global optimisation methods for parameter estimation were used as these gave the best fit when randomising the start values of each rate constant. The first was an evolutionary programming (EP) algorithm which is a computational technique which mimics evolution where populations evolve by the action of variation (mutations) and selection.[39] The size of the population is chosen by the user and refers to the number of individuals that survive after each generation. The number of generations is also selected by the user and determines the number of generations that the population is evolved by the algorithm.

The second parameter optimisation method is differential evolution (DE) which also searches large areas of candidate solutions.[40] The algorithm follows an evolutionary process and also requires user defined population sizes and generation numbers. Using each method, the goodness of fit between calculated and experimental data was scored using an objective function value (which is minimised using the algorithm).

For parameter estimations involving a dissociative mechanism, optimisations were repeated ten times to search for multiple possible solutions for the rate constants. The method, number of generations and population sizes were varied to assess the variation in possible solutions for each rate constant (see Table S1). Errors are displayed as 3 × standard deviation of the parameter estimation.

Calculated values of $\Delta G^{\ddagger}_{Diss}$ and $\Delta G^{\ddagger}_{Asso}$ are obtained using the Eyring equation $\Delta G^{\ddagger}_{Diss/Asso} = -RT\ln[k_x h/kb\ T]$ where $k_x = k_1$, k_{-1}, k_2 or k_{-2}. ΔG_{Asso} was calculated using the equation: $\Delta G = -RT\ln(K_{eq})$, Where: $K_{eq} = k_{-1}/k_1$ or k_2/k_{-2}. ΔG was calculated using the same equation where $K_{eq} = [\mathrm{AC}]^2_{\mathrm{eq}}/[\mathrm{AB}]^2_{\mathrm{eq}}$ (where [AC] and [AB] are the concentrations of pseudorotaxane species) or ($k_1/k_{-1} \times k_2/k_{-2}$).

1.2 Mass Spectrometry

Collision-Induced Dissociation (CID) mass spectrometry samples were prepared in 4:1 toluene:methanol with 500 µM NaI, using final analyte concentrations of 200 µM. Ionisation and transfer to the gas phase were performed with a nano-electrospray ionization (nESI) source. Sample solutions were sprayed from borosilicate glass capillaries (World Precision Instruments, Stevenage, UK), which were pulled on the Flaming/Brown P-2000 laser puller (Sutter Instrument Company, Novato, CA, US). The capillary voltage (typically 1.0 - 1.8 kV) was applied through a platinum wire (Diameter 0.125 mm, Goodfellow, Huntingdon, UK) inserted into the nESI capillaries. The source temperature was set to 30 °C.

The Q Exactive Ultra-High-Mass-Range (UHMR) Hybrid Quadrupole-Orbitrap Mass Spectrometer (Thermo Fisher) was used for the derivation of the E_{50} values *via* CID. Target ions were isolated in a quadrupole filter, accelerated to a user-defined kinetic energy (E_{lab}: 0 - 150 eV) and injected into the

higher-energy C-trap dissociation (HCD) cell, which contained nitrogen gas (trapping gas pressure parameter: 2.0). Fragment ions and non-fragmented precursor ions were transferred to the Orbitrap mass analyser (resolution: 25000, AGC target: 3E6 ions, maximum inject time: 100 ms).

E_{50} values were obtained using a method described in our previous works.[31, 32] Mass spectra were recorded at different collision energies and the share of the precursor ion count, relative to the total ion count ("survival yield"), was plotted against the collisional energy in the centre-of-mass frame (E_{com}). Survival yield plots were fitted with a sigmoidal Hill function (Hill1 function in OriginPro 2020b), yielding the point (E_{50}) at which the survival yield reaches 0.5 or 50%. This E_{50} value is known as a relative measure of precursor ion stability.[28 – 30]

2. Experimental Procedures

Me_2NH_2·1

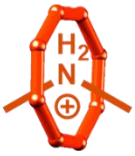

$CrF_3.4H_2O$ (6.0 g, 33 mmol), tert-butylacetic acid (48 g, 41 mmol), Dimethylammonium dimethylcarbamate (0.84 g, 6.3 mmol), and $CoCO_3$ (1.5 g, 7.6 mmol) were heated in a Teflon flask at 160 °C for 24 h with stirring. The flask was then cooled to room temperature and acetonitrile (100 mL) was added and stirred for 1 h. The green precipitate was filtered and washed with acetonitrile (250 mL). The solid was stirred for 1 h in acetone (350 mL) and filtered. The solvent was removed under a reduced pressure and residue washed with acetonitrile (150 mL) and dried under vacuum to yield a green powder, 11.2 g (96 % calc. from Cr used). Elemental analysis (%) calcd. for $C_{98}H_{184}CoCr_7F_8NO_{32}$: C 47.78, H 7.53, Co 2.39, Cr 14.78, N 0.57; found: C 47.90, H 7.57, Co 2.99, Cr 12.91, N 0.62. Positive ESI-MS (dissolved in THF, run in MeOH): m/z = 2486.6 $[M+Na]^+$.

Pr_2NH_2·1

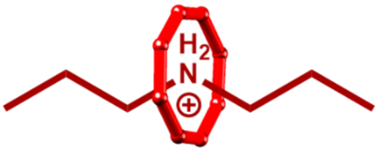

$CrF_3.4H_2O$ (5.0 g, 28 mmol), tert-butylacetic acid (20 g, 17 mmol), dipropylamine (1.5 g, 15 mmol), and $CoCO_3$ (1.5 g, 13 mmol) were heated in a Teflon flask at 160 °C for 6 hours with stirring. The flask was then cooled to room temperature and acetonitrile (50 mL) was added and stirred for 1 h. The green precipitate was filtered and washed with acetonitrile (200 mL). The solid was stirred for 1 h in acetone (100 mL) and filtered. The solvent was removed under a reduced pressure and residue washed with acetonitrile (100 mL) and dried under vacuum to yield a green powder, 7.0 g (70 % calc. from Cr used). Elemental analysis (%) calcd. for $C_{102}H_{192}CoCr_7F_8NO_{32}$: C 48.63, H 7.68, Co 2.34, Cr 14.45, N 0.56; found: C 48.61, H 7.77, Co 2.38, Cr 13.77, N 0.61. Positive ESI-MS (dissolved in THF, run in MeOH): m/z = 2541.8 $[M+Na]^+$.

Et_2NH_2·1

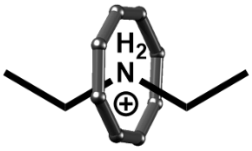

Was obtained in the same way as described for **Pr_2NH_2·1** but using diethylamine (1.1 g, 15 mmol) in place of dipropylamine to yield a green powder, 7.2 g (73 % calc. from Cr used). Elemental analysis (%) calcd. for $C_{100}H_{188}CoCr_7F_8NO_{32}$: C 48.21, H 7.61, Co 2.37, Cr 14.61, N 0.56; found: C 48.21, H 7.71, Co 2.34, Cr 13.92, N 0.55. Positive ESI-MS (dissolved in THF, run in MeOH): m/z = 2514.8 $[M+Na]^+$.

Bu_2NH_2·1

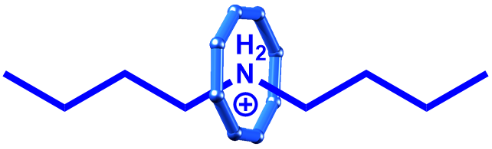

$CrF_3.4H_2O$ (5.0 g, 28 mmol), tert-butylacetic acid (18 g, 160 mmol), dibutylamine (1.9 g, 15 mmol), and $CoCO_3$ (1.5 g, 13 mmol) were heated in a Teflon flask at 160 °C for 6 hours with stirring. The flask was then cooled to room temperature and methanol (100 mL) was added and stirred for 10 minutes before filtering. Small portions (50 mL) of deionised water was added to the filtrate and stirred for 3 h. The solution was decanted from the precipitate and stirred in methanol (100 mL) overnight. The solid was filtered and washed with methanol (50 mL) and dried under vacuum to yield a green powder, 3.6 g (36 % calc. from Cr used). Elemental analysis (%) calcd. for $C_{104}H_{196}CoCr_7F_8NO_{32}$: C 49.03, H 7.76, Co 2.31, Cr 14.29, N 0.55; found: C 48.66, H 7.77, Co 2.17, Cr 13.68, N 0.53. Positive ESI-MS (dissolved in THF, run in MeOH): m/z = 2569.9 $[M+Na]^+$.

Pe_2NH_2·1

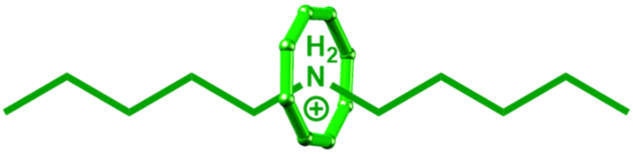

Was obtained in the same way as described for **Bu_2NH_2·1** but using dipentylamine (2.3 g, 15 mmol) in place of dibutylamine and after isolating the precipitate by decantation, was stirred in acetonitrile (100 mL) instead of methanol. A green powder was isolated, 2.8 g (28 % calc. from Cr used). Elemental analysis (%) calcd. for $C_{106}H_{200}CoCr_7F_8NO_{32}$: C 49.43, H 7.83, Co 2.29, Cr 14.13, N 0.54; found: C 49.14, H 7.87, Co 2.22, Cr 13.51, N 0.58. Positive ESI-MS (dissolved in THF, run in MeOH): m/z = 2598.9 $[M+Na]^+$.

Hx_2NH_2·1

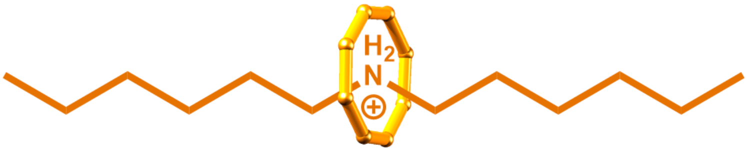

$CrF_3.4H_2O$ (5.0 g, 28 mmol), tert-butylacetic acid (18 g, 155 mmol), dihexylamine (2.8 g, 15 mmol), and $CoCO_3$ (1.5 g, 13 mmol) were heated in a Teflon flask at 160 °C for 6 hours with stirring. The flask was then cooled to room temperature and methanol (70 mL) was added and stirred for 4 h. The green precipitate was then filtered and washed with methanol (100 mL). The precipitate was stirred in acetone (100 mL) for 1 h before filtering. Solvent from the filtrate was removed under a reduced pressure before washing with acetonitrile (100 mL) and dried under vacuum to give a green powder, 3.2 g (31 % calc. from Cr used). Elemental analysis (%) calcd. for $C_{108}H_{204}CoCr_7F_8NO_{32}$: C 49.82, H 7.90, Co 2.26, Cr 13.98, N 0.54; found: C 49.93, H 8.03, Co 2.08, Cr 12.93, N 0.51. Positive ESI-MS (dissolved in THF, run in MeOH): m/z = 2625.9 $[M+Na]^+$.

Oc_2NH2·1

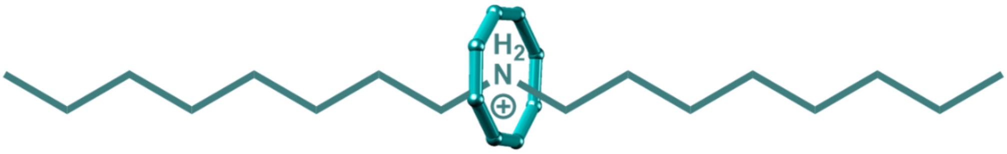

$CrF_3.4H_2O$ (5.0 g, 28 mmol), tert-butylacetic acid (15 g, 130 mmol), dioctylamine (2.7 g, 11 mmol), and $CoCO_3$ (1.5 g, 13 mmol) were heated in a Teflon flask at 160 °C for 5 hours with stirring. The flask was then cooled to room temperature and methanol (70 mL) was added and stirred for 4 h. The green precipitate was then filtered and washed with methanol (100 mL). The precipitate was stirred in acetone (150 mL) for 30 minutes before filtering. Solvent from the filtrate was removed under a reduced pressure before washing with acetonitrile (50 mL) and dried under vacuum to give a green powder, 2.5 g (23 % calc. from Cr used). Elemental analysis (%) calcd. for $C_{112}H_{212}CoCr_7F_8NO_{32}$: C 50.58, H 8.03, Co 2.22, Cr 13.68, N 0.53; found: C 50.73, H 8.13, Co 2.19, Cr 13.14, N 0.57. Positive ESI-MS (dissolved in THF, run in MeOH): m/z = 2683.0 $[M+Na]^+$.

$PrNH_3$·1

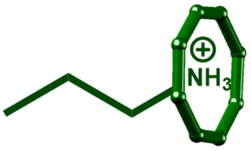

$CrF_3.4H_2O$ (6.0 g, 33 mmol), tert-butylacetic acid (34 g, 290 mmol), propylamine (0.7 g, 12 mmol), and $CoCO_3$ (0.96 g, 8.1 mmol) were heated in a Teflon flask at 160 °C for 26 hours with stirring. The flask was then cooled to room temperature and acetone (80 mL) was added and stirred for 15 minutes. The solution was filtered and solvent removed under a reduced pressure. Methanol (70 mL) was added to the resultant residue and stirred for 1 h before being left to stand at room temperature for 48 h. The green crystalline product was filtered and washed with methanol (50 mL) and dried under vacuum to

give a green powder, 9.0 g (77 % calc. from Cr used). Elemental analysis (%) calcd. for $C_{99}H_{186}CoCr_7F_8NO_{32}$: C 48.00, H 7.57, Co 2.38, Cr 14.69, N 0.57; found: C 47.63, H 7.60, Co 2.31, Cr 13.78, N 0.50. Positive ESI-MS (dissolved in THF, run in MeOH): m/z = 2499.8 $[M+Na]^+$.

$EtNH_3$·1

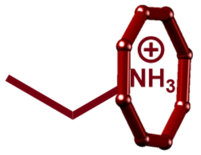

$CrF_3.4H_2O$ (6.0 g, 33 mmol), tert-butylacetic acid (34 g, 290 mmol), ethylamine in THF solution (6.0 mL, 12 mmol), and $CoCO_3$ (1.0 g, 8.4 mmol) were heated in a Teflon flask at 150 °C for 26 hours with stirring. The flask was then cooled to room temperature and acetone (80 mL) was added and stirred for 15 minutes. The solution was filtered and solvent removed under a reduced pressure. Methanol (70 mL) was added to the resultant residue and stirred for 1 h before being left to stand at room temperature for 48 h. The green crystalline product was filtered and washed with methanol (50 mL) and dried under vacuum to give a green powder, 4.5 g (39 % calc. from Cr used). Elemental analysis (%) calcd. for $C_{98}H_{184}CoCr_7F_8NO_{32}$: C 47.78, H 7.53, Co 2.39, Cr 14.78, N 0.57; found: C 47.76, H 7.58, Co 2.37, Cr 14.14, N 0.51. Positive ESI-MS (dissolved in THF, run in MeOH): m/z = 2485.8 $[M+Na]^+$.

$Allyl_2NH_2$·1

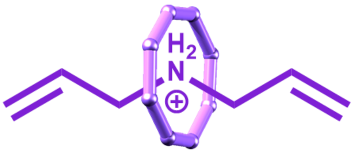

$CrF_3.4H_2O$ (10 g, 55 mmol), tert-butylacetic acid (40 g, 340 mmol), diallylamine (2.4 g, 25 mmol), and $CoCO_3$ (2.0 g, 17 mmol) were heated in a Teflon flask at 140 °C for 27 hours with stirring. The flask was then cooled to room temperature and acetonitrile (100 mL) was added and stirred for 4 h. The precipitate was filtered and washed with acetonitrile (200 mL) and dried under vacuum. The solid was dissolved in hexane and filtered. The solvent was removed from the filtrate under a reduced pressure before dissolving in tert-butyl methyl ether (200 mL) and filtered. The solvent was removed under a reduced pressure before washing with acetonitrile and drying under vacuum to give a green powder, 12 g (61 % calc. from Cr used). Elemental analysis (%) calcd. for $C_{102}H_{188}CoCr_7F_8NO_{32}$: C 48.70, H 7.53, Co 2.34, Cr 14.47, N 0.56; found: C 48.61, H 7.56, Co 2.26, Cr 14.11, N 0.50. Positive ESI-MS (dissolved in THF, run in MeOH): m/z = 2537.8 $[M+Na]^+$.

EtNH$_2$Me·1

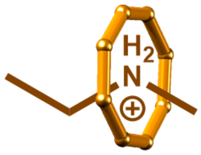

Methylethylamine (0.035 mL, 0.40 mmol) was dissolved in acetone (10 mL) alongside **PrNH$_3$·1** (0.50 g, 0.20 mmol) and stirred for 1 h. The solvent was removed under a reduced pressure and re-dissolved in hot acetonitrile (15 mL) and stirred for 15 minutes. The resultant solution was cooled to 4 °C for 6 h. The green crystalline product was filtered, washed with acetonitrile (50 mL) and dried under vacuum to give a green powder, 0.45 g (90 % calc. from **PrNH$_3$·1** used). Elemental analysis (%) calcd. for $C_{99}H_{186}CoCr_7F_8NO_{32}$: C 48.00, H 7.57, Co 2.38, Cr 14.69, N 0.57; found: C 48.14, H 7.48, Co 2.26, Cr 14.26, N 0.43. Positive ESI-MS (dissolved in THF, run in MeOH): m/z = 2499.8 $[M+Na]^+$.

MeNH$_2$Pr·1

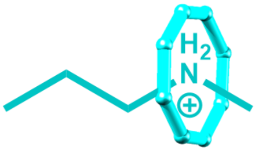

Was obtained in the same way as described for **EtNH$_2$Me·1** but using methylpropylamine (0.041 mL, 0.40 mmol) in place of methylethylamine to yield a green powder, 0.41 g (81 % calc. from **PrNH$_3$·1** used). Elemental analysis (%) calcd. for $C_{100}H_{188}CoCr_7F_8NO_{32}$: C 48.21, H 7.61, Co 2.37, Cr 14.61, N 0.56; found: C 48.20, H 7.66, Co 2.26, Cr 14.21, N 0.48. Positive ESI-MS (dissolved in THF, run in MeOH): m/z = 2513.8 $[M+Na]^+$.

EtNH$_2$Pr·1

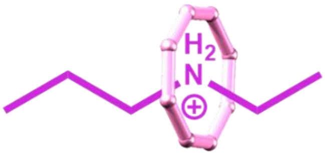

Was obtained in the same way as described for **EtNH$_2$Me·1** but using ethylpropylamine (0.049 mL, 0.40 mmol) in place of methylethylamine to yield a green powder, 0.46 g (91 % calc. from **PrNH$_3$·1** used). Elemental analysis (%) calcd. for $C_{101}H_{190}CoCr_7F_8NO_{32}$: C 48.42, H 7.64, Co 2.35, Cr 14.53, N 0.56; found: C 48.61, H 7.69, Co 2.24, Cr 14.11, N 0.56. Positive ESI-MS (dissolved in THF, run in MeOH): m/z = 2527.8 $[M+Na]^+$.

3. ^{1}H NMR Assigned Spectra

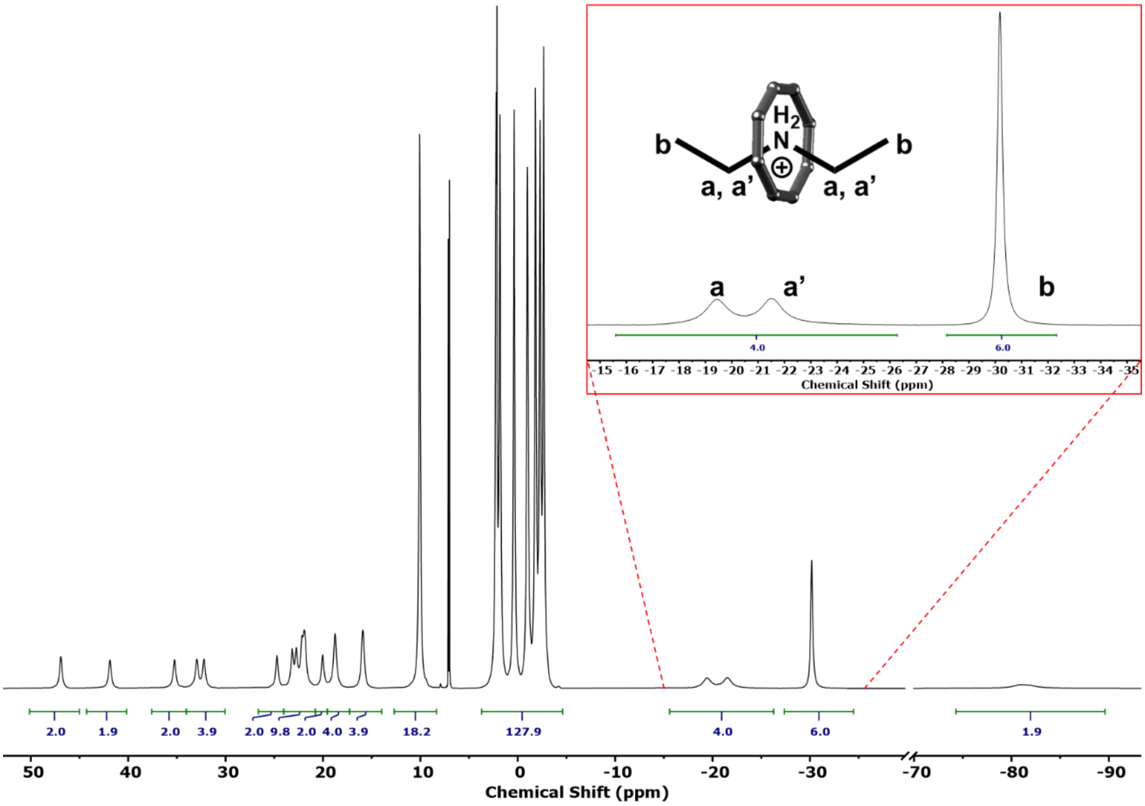

Figure S1. ^{1}H NMR (500 MHz, 298 K) baseline corrected spectra of **Et_2NH_2·1** in toluene-d_8 including an assigned insert of the pseudorotaxane thread. Integrals assigned to carboxylate methyl groups are larger than expected due to overlap with the toluene protio-impurity.

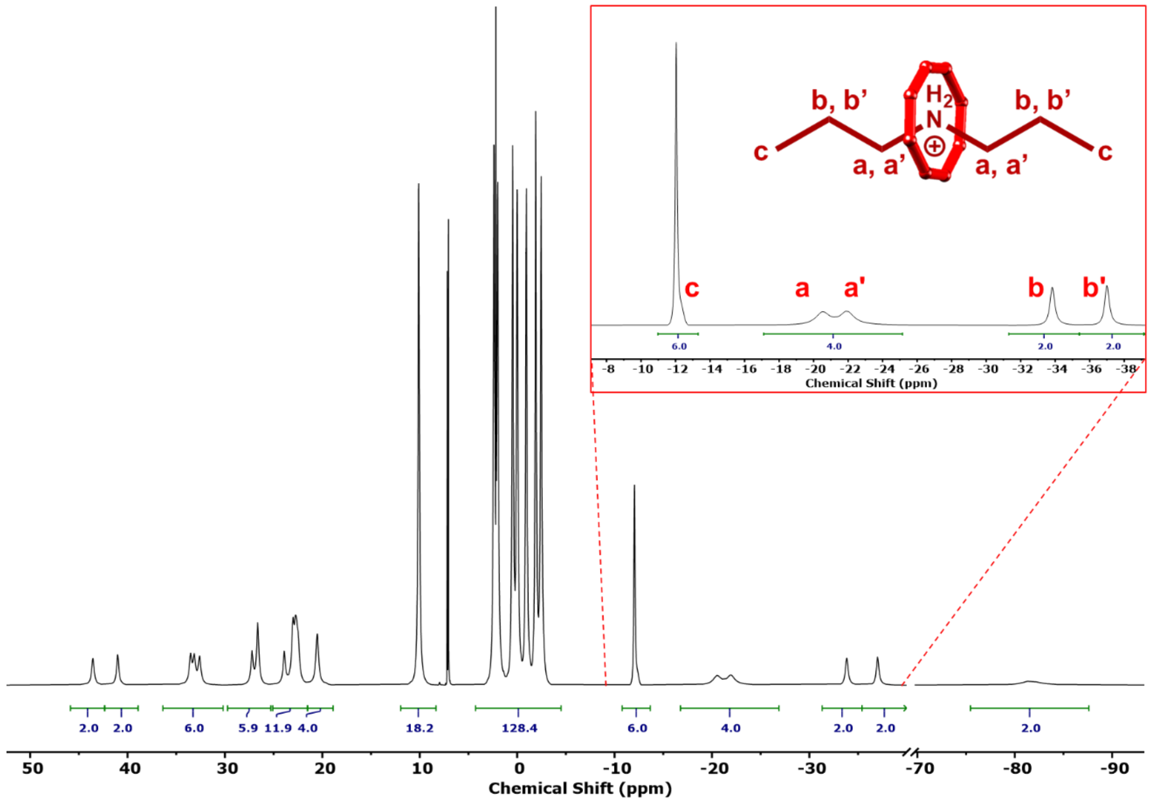

Figure S2. ^{1}H NMR (500 MHz, 298 K) baseline corrected spectra of **Pr_2NH_2·1** in toluene-d_8 including an assigned insert of the pseudorotaxane thread. Integrals assigned to carboxylate methyl groups are larger than expected due to overlap with the toluene protio-impurity.

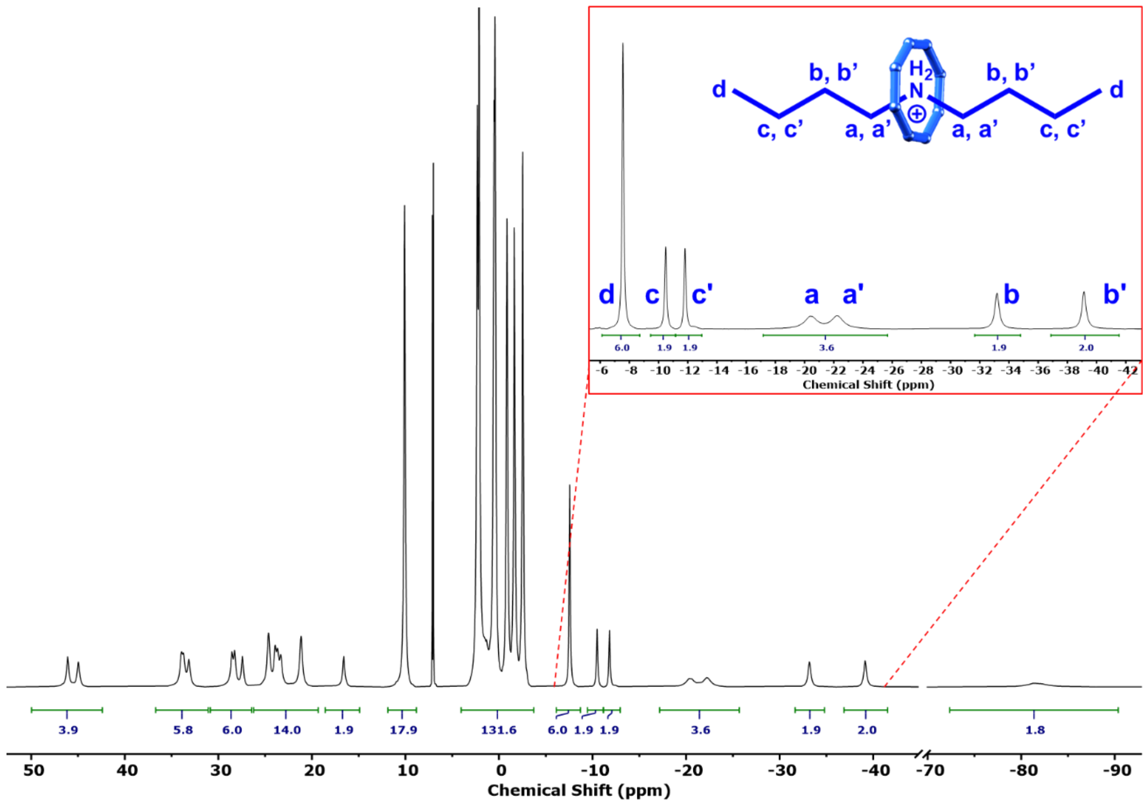

Figure S3. ^{1}H NMR (500 MHz, 298 K) baseline corrected spectra of **Bu_2NH_2·1** in toluene-d_8 including an assigned insert of the pseudorotaxane thread. Integrals assigned to carboxylate methyl groups are larger than expected due to overlap with the toluene protio-impurity.

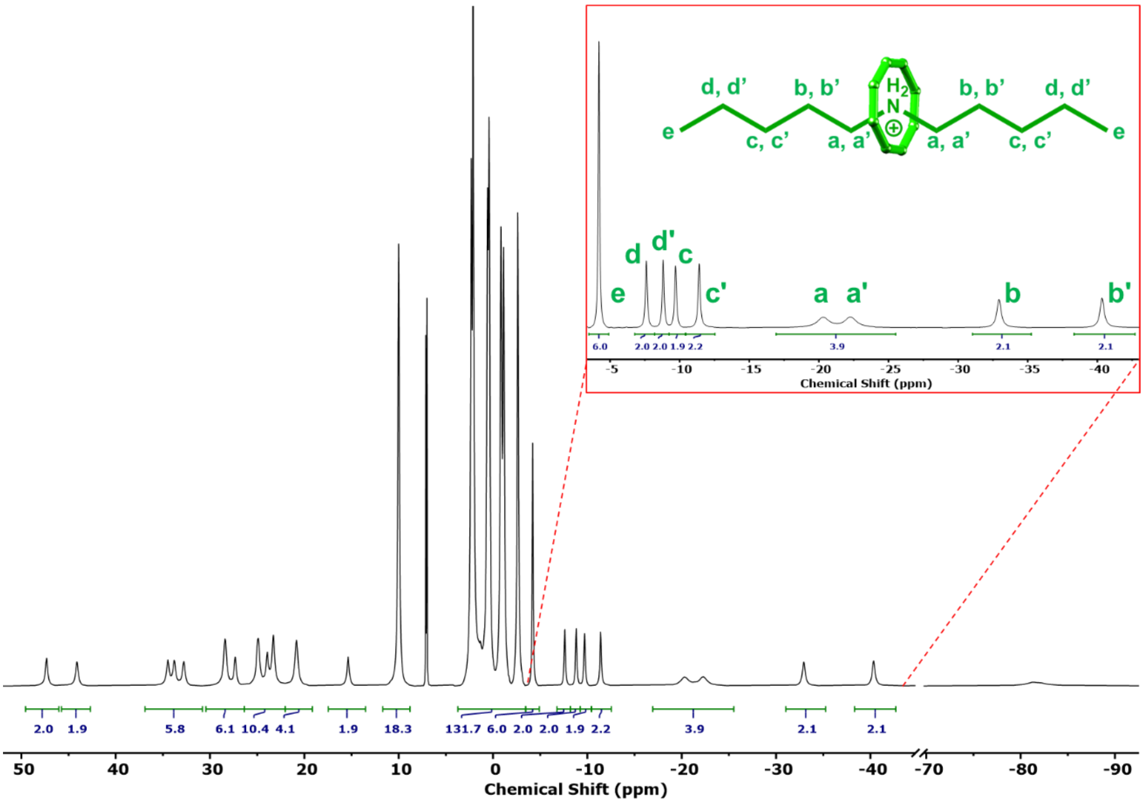

Figure S4. ^1H NMR (500 MHz, 298 K) baseline corrected spectra of **Pe_2NH_2·1** in toluene-d_8 including an assigned insert of the pseudorotaxane thread. Integrals assigned to carboxylate methyl groups are larger than expected due to overlap with the toluene protio-impurity.

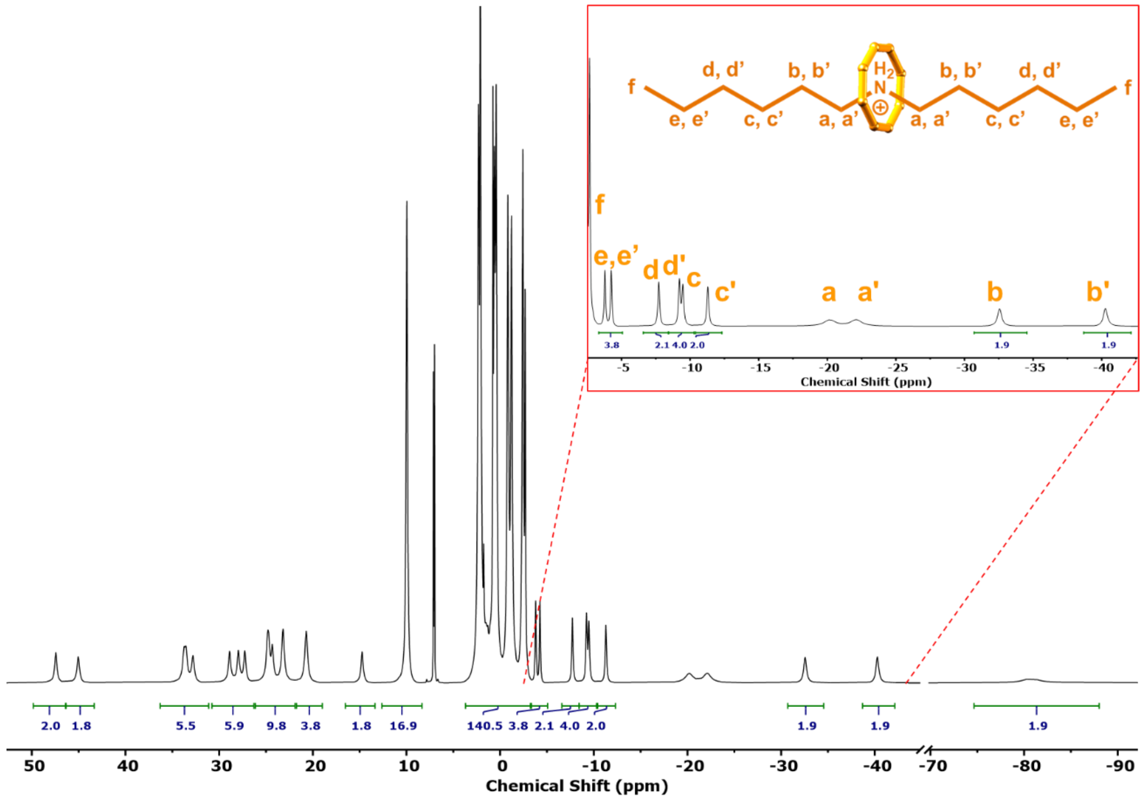

Figure S5. ^{1}H NMR (500 MHz, 298 K) baseline corrected spectra of **Hx_2NH_2·1** in toluene-d_8 including an assigned insert of the pseudorotaxane thread. Integrals assigned to carboxylate methyl groups are larger than expected due to overlap with the toluene protio-impurity.

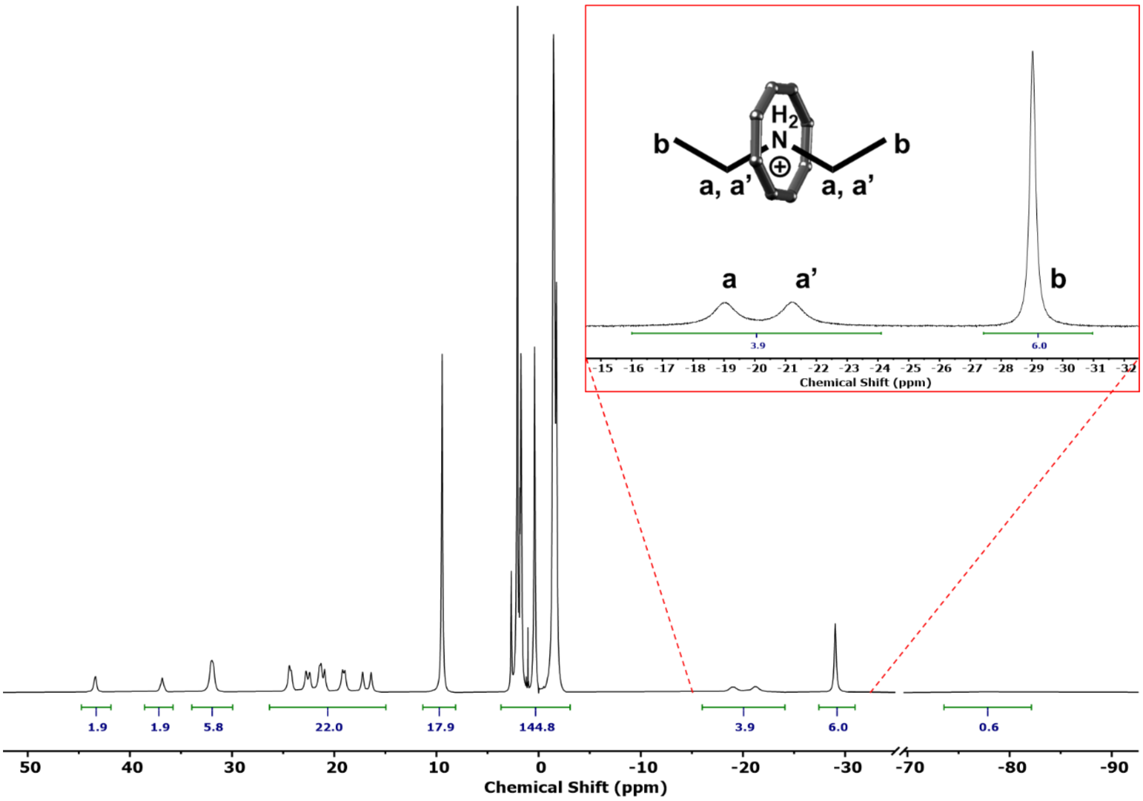

Figure S6. ^{1}H NMR (500 MHz, 298 K) baseline corrected spectra of $\mathbf{Et_2NH_2{\cdot}1}$ in acetone-d_6 including an assigned insert of the pseudorotaxane thread. Integrals assigned to carboxylate methyl groups are larger than expected due to overlap with the acetone protio-impurity. Broad N**H** signals often give integrals lower than expected.

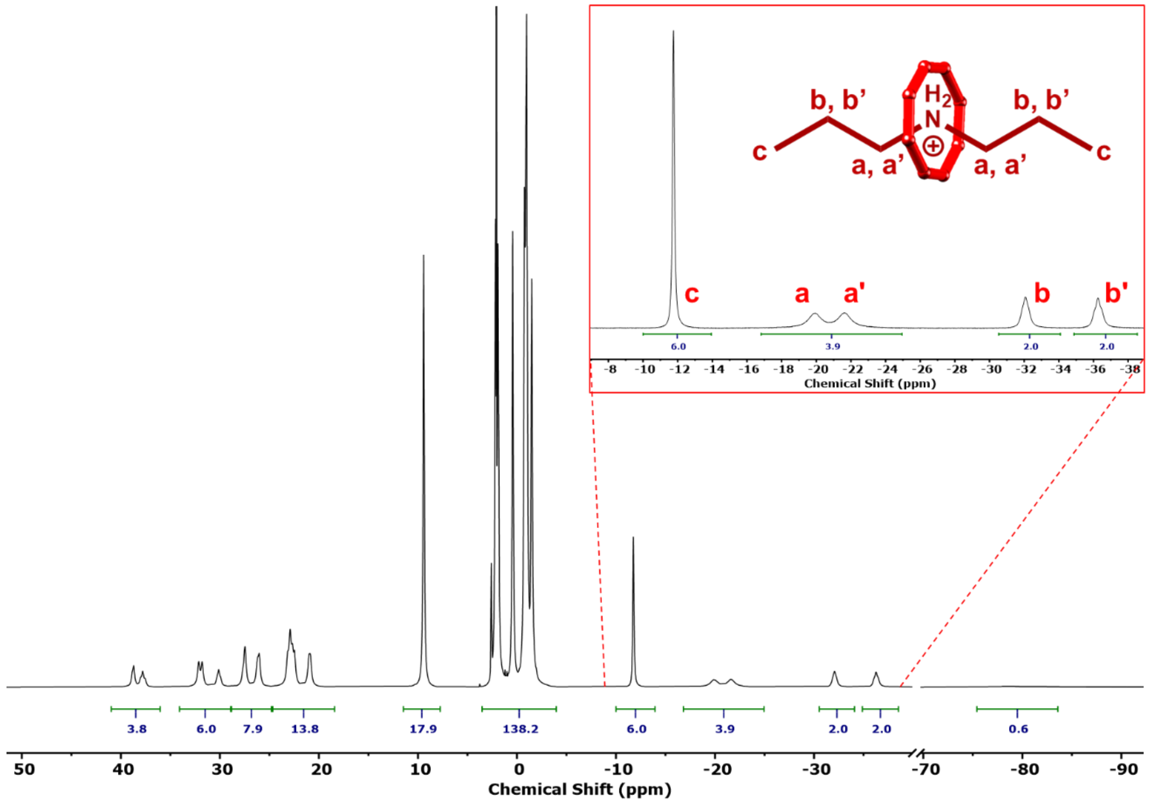

Figure S7. ^{1}H NMR (500 MHz, 298 K) baseline corrected spectra of **Pr_2NH_2·1** in acetone-d_6 including an assigned insert of the pseudorotaxane thread. Integrals assigned to carboxylate methyl groups are larger than expected due to overlap with the acetone protio-impurity. Broad N**H** signals often give integrals lower than expected.

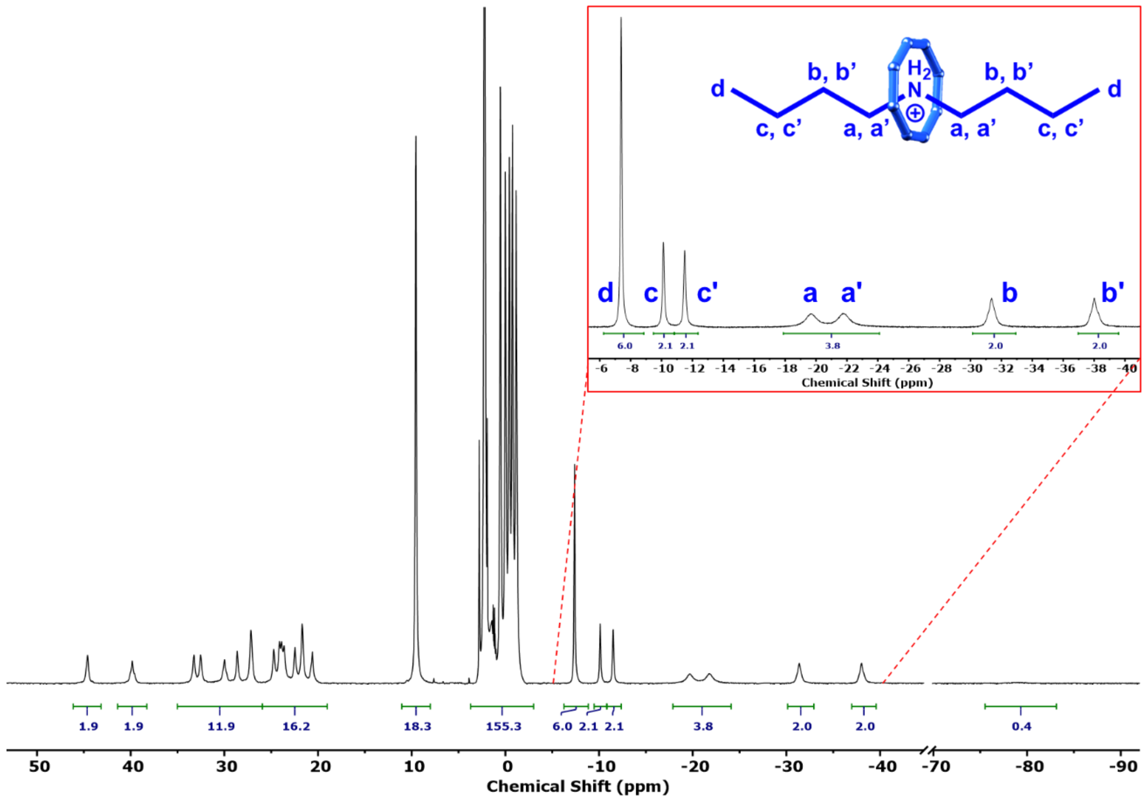

Figure S8. ^{1}H NMR (500 MHz, 298 K) baseline corrected spectra of **Bu_2NH_2·1** in acetone-d_6 including an assigned insert of the pseudorotaxane thread. Integrals assigned to carboxylate methyl groups are larger than expected due to overlap with the acetone protio-impurity. Broad N**H** signals often give integrals lower than expected.

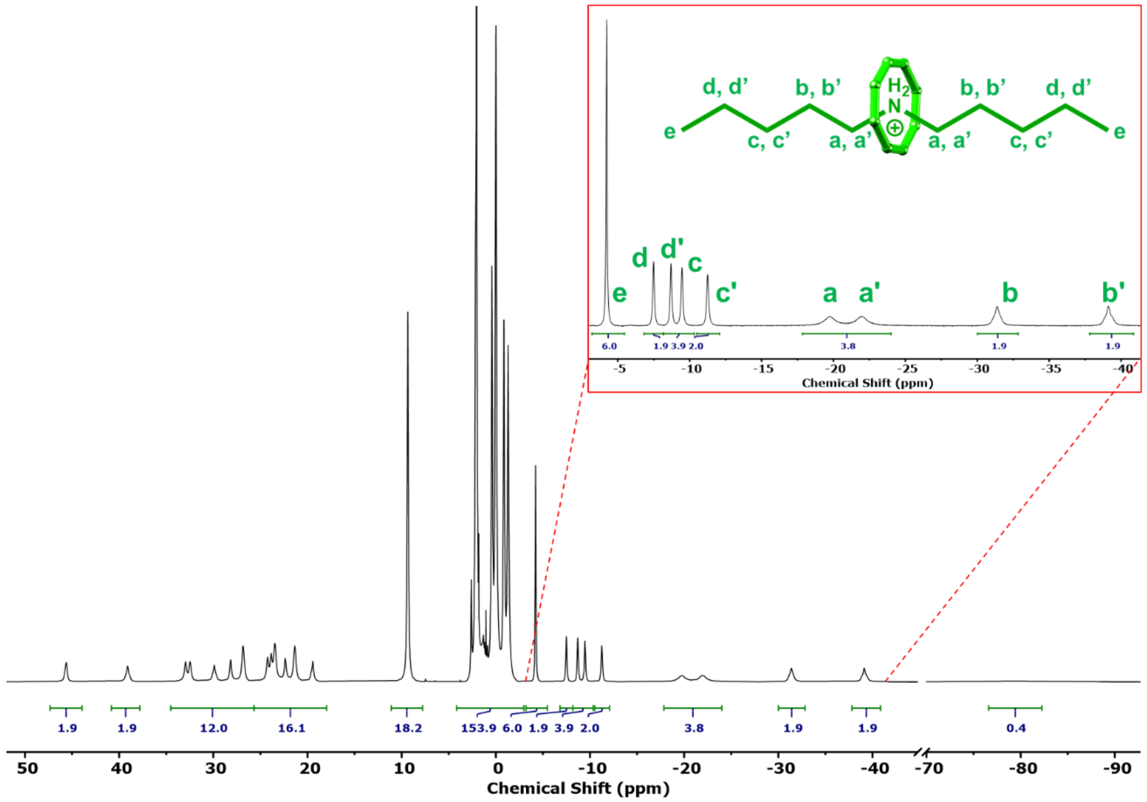

Figure S9. ^{1}H NMR (500 MHz, 298 K) baseline corrected spectra of **Pe_2NH_2·1** in acetone-d_6 including an assigned insert of the pseudorotaxane thread. Integrals assigned to carboxylate methyl groups are larger than expected due to overlap with the acetone protio-impurity. Broad N**H** signals often give integrals lower than expected.

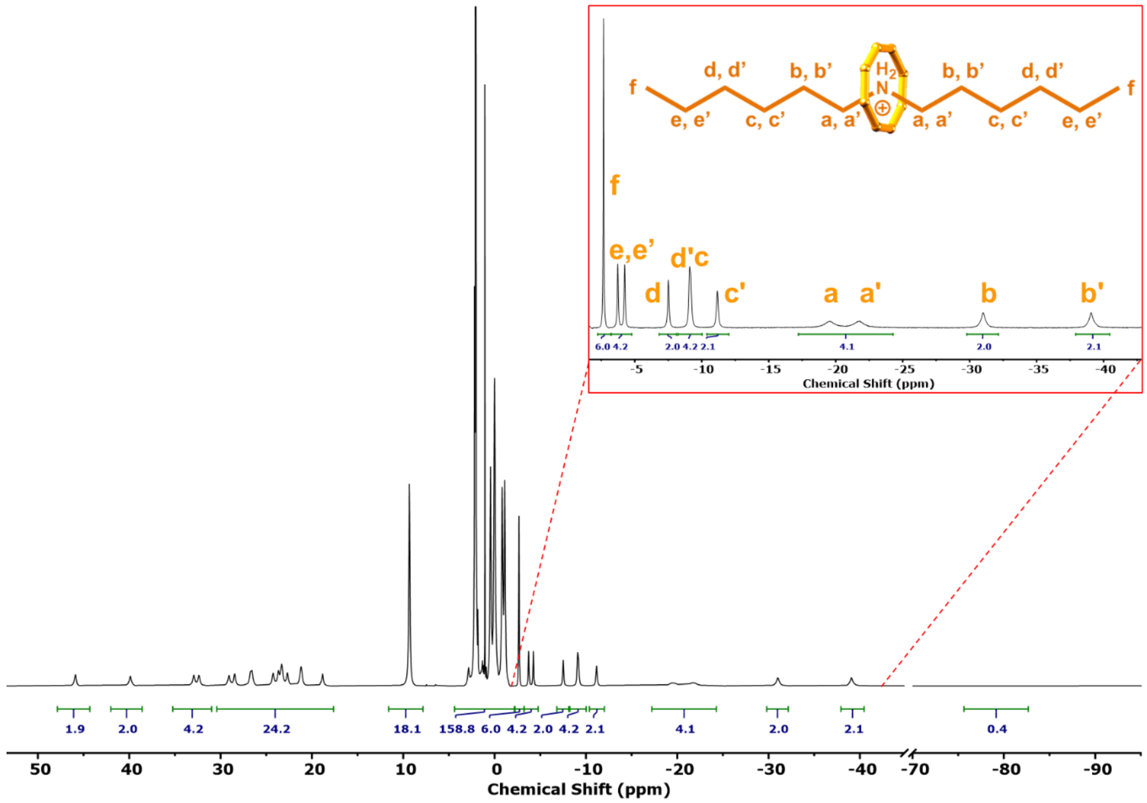

Figure S10. ^{1}H NMR (500 MHz, 298 K) baseline corrected spectra of **Hx$_2$NH$_2$·1** in acetone-*d*$_6$ including an assigned insert of the pseudorotaxane thread. Integrals assigned to carboxylate methyl groups are larger than expected due to overlap with the acetone protio-impurity. Broad N**H** signals often give integrals lower than expected.

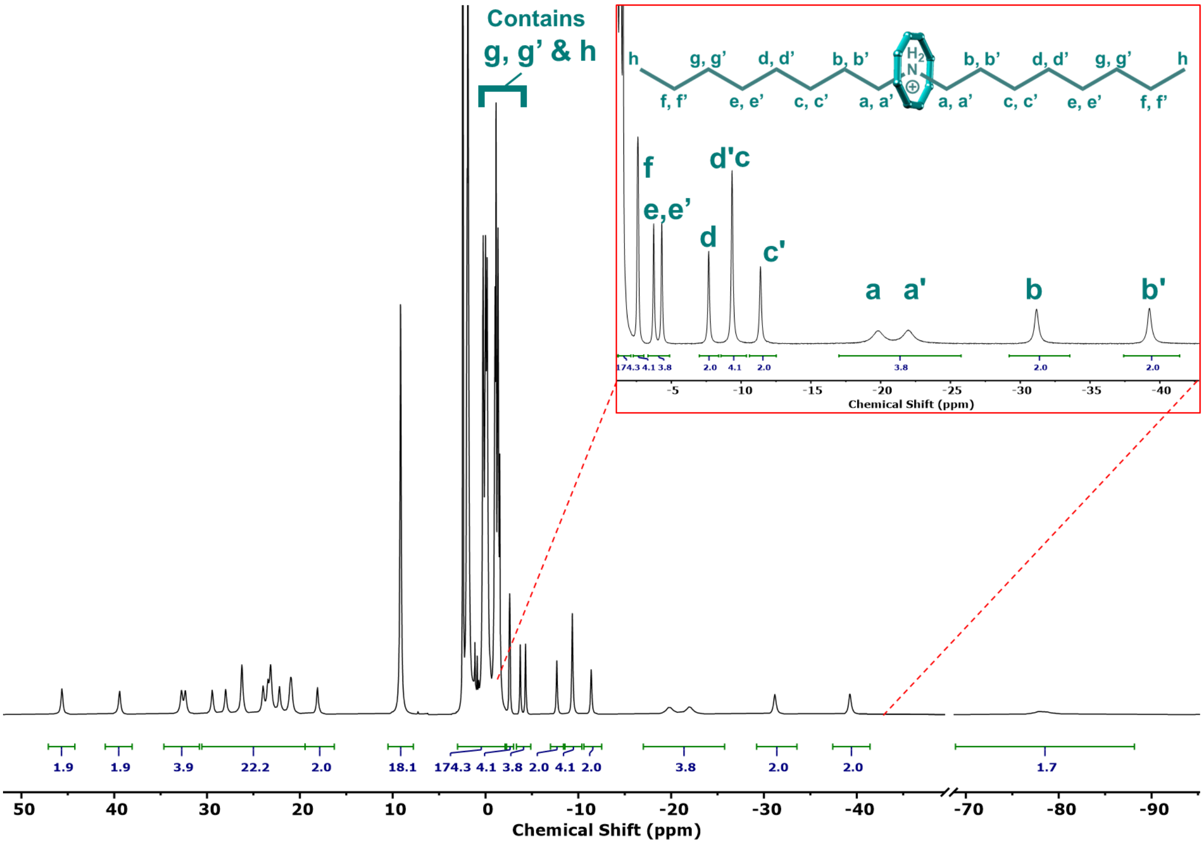

Figure S11. ^{1}H NMR (500 MHz, 298 K) baseline corrected spectra of **Oc_2NH_2·1** in acetone-d_6 including an assigned insert of the pseudorotaxane thread. Integrals assigned to carboxylate methyl groups are larger than expected due to overlap with the acetone protio-impurity. Broad N**H** signals often give integrals lower than expected.

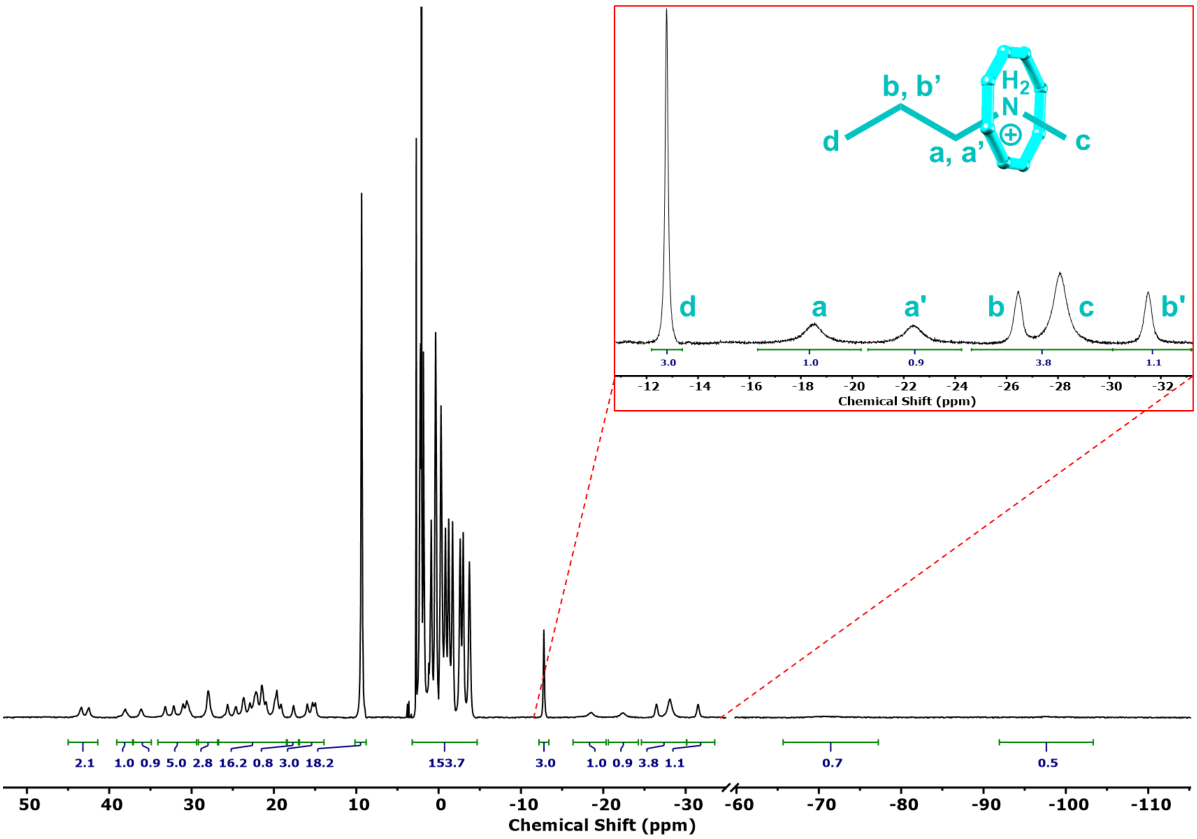

Figure S12. ^{1}H NMR (500 MHz, 298 K) baseline corrected spectra of **MeNH$_2$Pr·1** in acetone-d_6 including an assigned insert of the pseudorotaxane thread. Integrals assigned to carboxylate methyl groups are larger than expected due to overlap with the acetone protio-impurity. Broad N**H** signals often give integrals lower than expected.

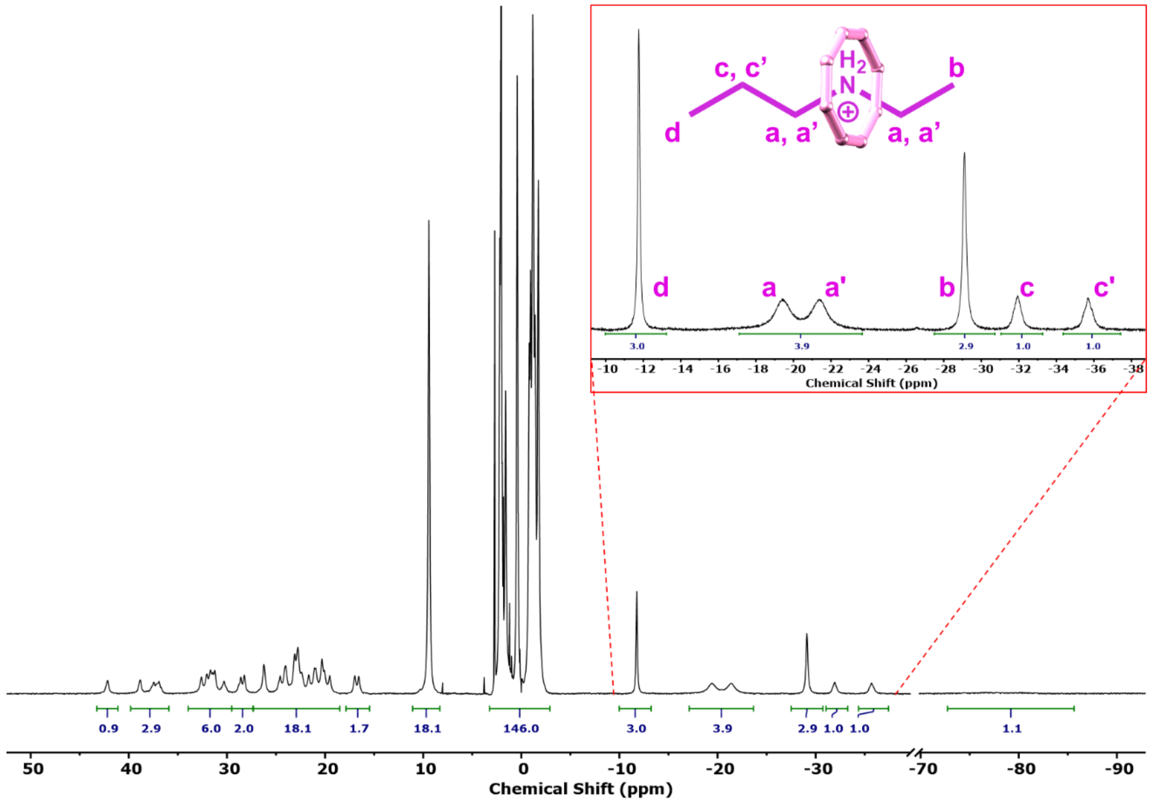

Figure S13. ^{1}H NMR (500 MHz, 298 K) baseline corrected spectra of **EtNH₂Pr·1** in acetone-d_6 including an assigned insert of the pseudorotaxane thread. Integrals assigned to carboxylate methyl groups are larger than expected due to overlap with the acetone protio-impurity. Broad N**H** signals often give integrals lower than expected.

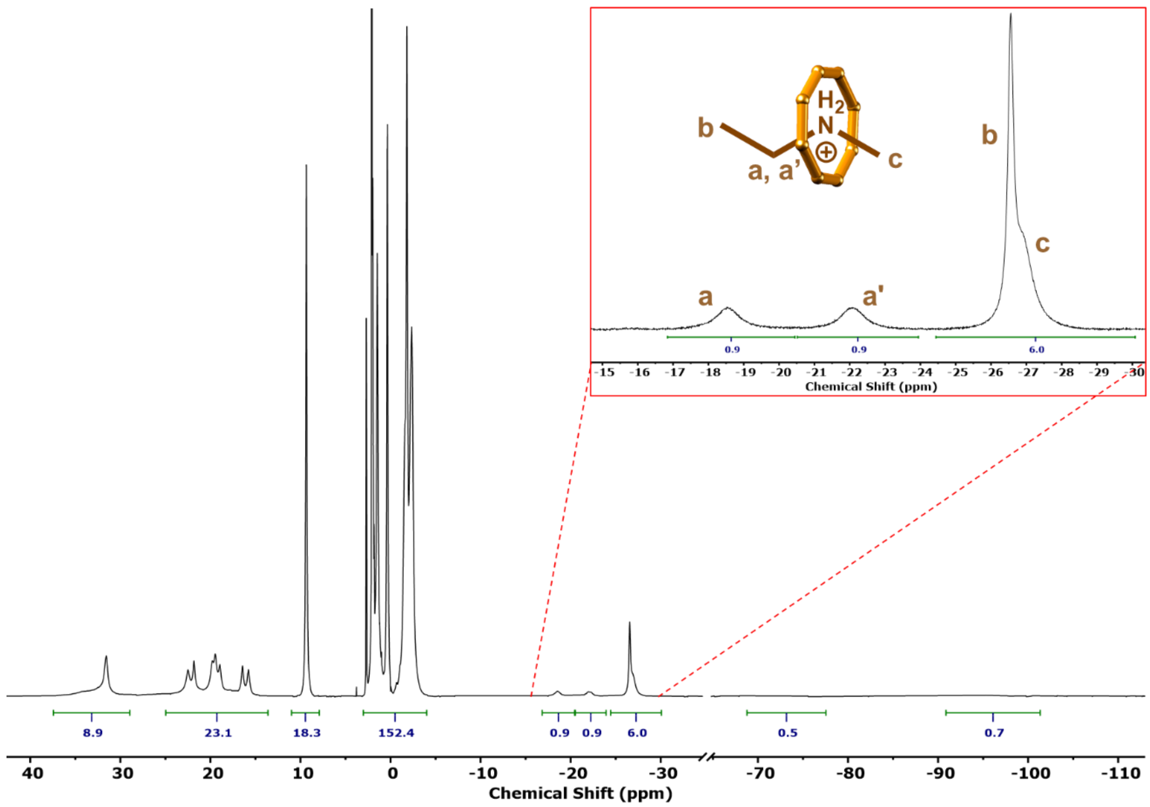

Figure S14. ^{1}H NMR (500 MHz, 298 K) baseline corrected spectra of **EtNH$_2$Me·1** in acetone-d_6 including an assigned insert of the pseudorotaxane thread. Integrals assigned to carboxylate methyl groups are larger than expected due to overlap with the acetone protio-impurity. Broad N**H** signals often give integrals lower than expected.

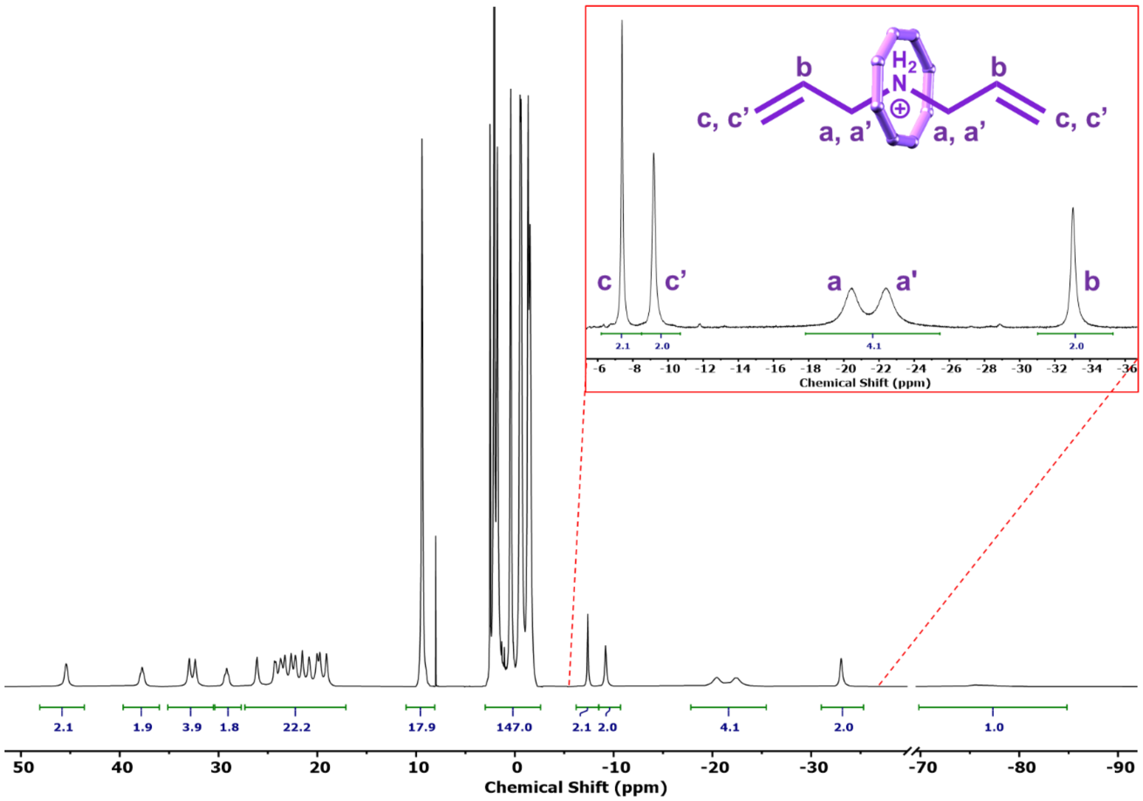

Figure S15. ^{1}H NMR (500 MHz, 298 K) baseline corrected spectra of **Allyl$_2$NH$_2$·1** in acetone-d_6 including an assigned insert of the pseudorotaxane thread. Integrals assigned to carboxylate methyl groups are larger than expected due to overlap with the acetone protio-impurity. Broad N**H** signals often give integrals lower than expected.

4. Thread Exchange Experiments

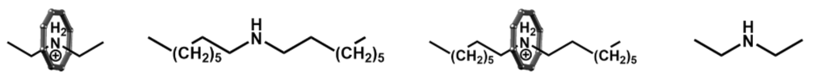

X	AB	C	AC	B
**Reaction 1 $[X]_0$**	11 mM	11 mM	0 mM	0 mM
**Reaction 2 $[X]_0$**	0 mM	0 mM	11 mM	11 mM

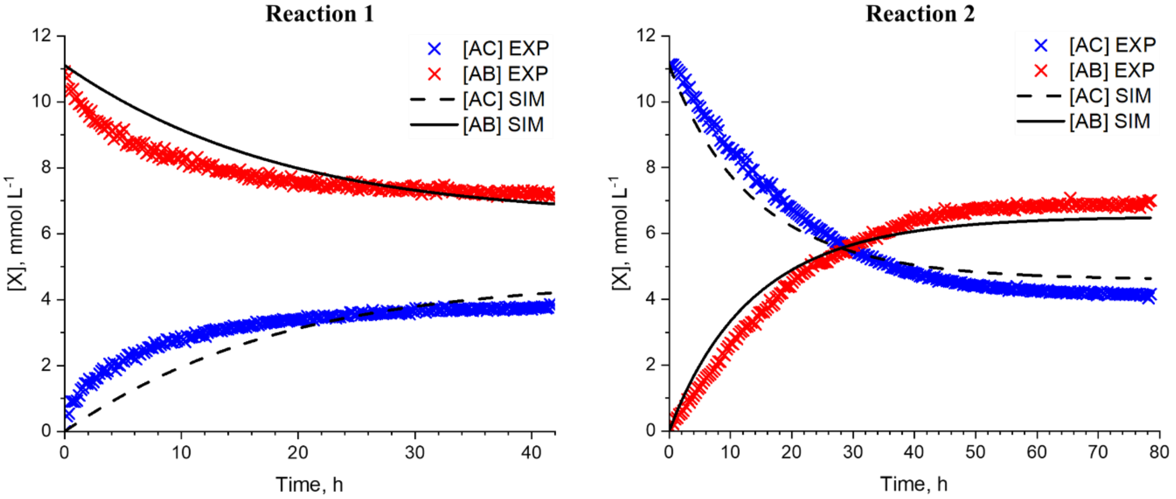

$$\mathrm{AB + C} \underset{k_{-1}}{\overset{k_1}{\rightleftharpoons}} \mathrm{ABC} \underset{k_{-2}}{\overset{k_2}{\rightleftharpoons}} \mathrm{AC + B}$$

COPASI Solution:
k_1= 5.5×10^{-4} ± 4.2×10^{-4} L mol^{-1} s^{-1}
k_{-1}= 1.3 ± 2.7×10^{3} s^{-1}
k_2= 2.7×10^{4}± 1.29×10^{6} s^{-1}
k_{-2}= 23 ± 5.5×10^{4} L mol^{-1} s^{-1}

Figure S16. Initial concentrations ($[X]_0$) used to set-up Reactions 1 and 2 (top). Kinetic plots for thread exchange reactions between psuedorotaxanes **Et_2NH_2·1** and **Oc_2NH_2·1** in acetone-d_6 at 313.15 K where: red crosses = $[AB]^{EXP}$, blue crosses = $[AC]^{EXP}$, black solid = $[AB]^{SIM}$, black dash = $[AC]^{SIM}$ (middle). Associative mechanism and estimated rate constant parameters from a best fit COPASI parameter estimation (bottom). Errors are displayed as 3 × standard deviation from the parameter estimation.

Table S1. Parameters used for the parameter estimation of rate constants k_1, k_{-1}, k_2 and k_{-2} (313.5 K) when fitting experimental ^{1}H NMR data to a dissociative mechanism. Parameter estimations have been repeated ten times varying the method, number of generations and population sizes used.

	Result 1	Result 2	Result 3	Result 4	Result 5
Method	EP	EP	DE	DE	EP
Number of Generations	4000	2000	2000	8000	8000
Population Size	100	100	100	10	10
Functionals Evaluated	400017	200017	419907	168006	80017
Objective Function Value	7.56221	7.62749	7.62594	7.62639	7.62635
k_{-2}, s^{-1}	7.41×10^{-6}	7.41×10^{-6}	7.42×10^{-6}	7.42×10^{-6}	7.42×10^{-6}
k_2, L mol^{-1} s^{-1}	1.38×10^{9}	18.2	9.19×10^{10}	1.09×10^{11}	2.66×10^{2}
k_{-1}, L mol^{-1} s^{-1}	2.04×10^{10}	2.71×10^{2}	1.36×10^{12}	1.61×10^{12}	3.93×10^{3}
k_1, s^{-1}	3.42×10^{-5}	3.42×10^{-5}	3.39×10^{-5}	3.39×10^{-5}	3.39×10^{-5}
K_{eq}	3.22	3.23	3.23	3.23	3.23
k_{-1}/k_2	14.9	14.9	14.8	14.8	14.8
k_1/k_{-2}	4.61	4.61	4.57	4.58	4.57
${}^{AB}\Delta G^{\ddagger}_{Diss}$, kJ mol^{-1}	104	104	104	104	104
${}^{AC}\Delta G^{\ddagger}_{Diss}$, kJ mol^{-1}	108	108	108	108	108
${}^{AB}\Delta G^{\ddagger}_{Asso}$, kJ mol^{-1}	15.0	62.2	4.09	3.65	55.3
${}^{AC}\Delta G^{\ddagger}_{Asso}$, kJ mol^{-1}	22.0	69.3	11.1	10.7	62.3
${}^{AB}\Delta G_{Asso}$, kJ mol^{-1}	-88.6	-41.4	-99.5	-100	-48.4
${}^{AC}\Delta G_{Asso}$, kJ mol^{-1}	-85.5	-38.3	-96.5	-96.9	-45.3

	Result 6	Result 7	Result 8	Result 9	Result 10
Method	DE	EP	EP	DE	DE
Number of Generations	1000	1000	5000	5000	10000
Population Size	500	500	500	500	10
Functionals Evaluated	1049467	500017	2500017	5249467	210006
Objective Function Value	7.62626	7.62647	7.62607	7.62593	7.62686
k_{-2}, s^{-1}	7.42×10^{-6}	7.42×10^{-6}	7.42×10^{-6}	7.42×10^{-6}	7.42×10^{-6}
k_2, L mol^{-1} s^{-1}	1.04×10^{11}	3.76×10^{8}	2.13×10^{7}	9.19×10^{10}	1.48×10^{11}
k_{-1}, L mol^{-1} s^{-1}	1.55×10^{12}	5.59×10^{9}	3.15×10^{8}	1.36×10^{12}	2.17×10^{12}
k_1, s^{-1}	3.40×10^{-5}	3.41×10^{-5}	3.40×10^{-5}	3.39×10^{-5}	3.37×10^{-5}
K_{eq}	3.23	3.23	3.23	3.23	3.23
k_{-1}/k_2	14.8	14.9	14.8	14.8	14.7
k_1/k_{-2}	4.58	4.59	4.58	4.57	4.55
${}^{AB}\Delta G^{\ddagger}_{Diss}$, kJ mol^{-1}	104	104	104	104	104
${}^{AC}\Delta G^{\ddagger}_{Diss}$, kJ mol^{-1}	108	108	108	108	108
${}^{AB}\Delta G^{\ddagger}_{Asso}$, kJ mol^{-1}	3.75	18.4	25.9	4.09	2.87
${}^{AC}\Delta G^{\ddagger}_{Asso}$, kJ mol^{-1}	10.8	25.4	32.9	11.1	9.86
${}^{AB}\Delta G_{Asso}$, kJ mol^{-1}	-99.9	-85.2	-77.7	-99.5	-101
${}^{AC}\Delta G_{Asso}$, kJ mol^{-1}	-96.8	-82.2	-74.7	-96.5	-97.7

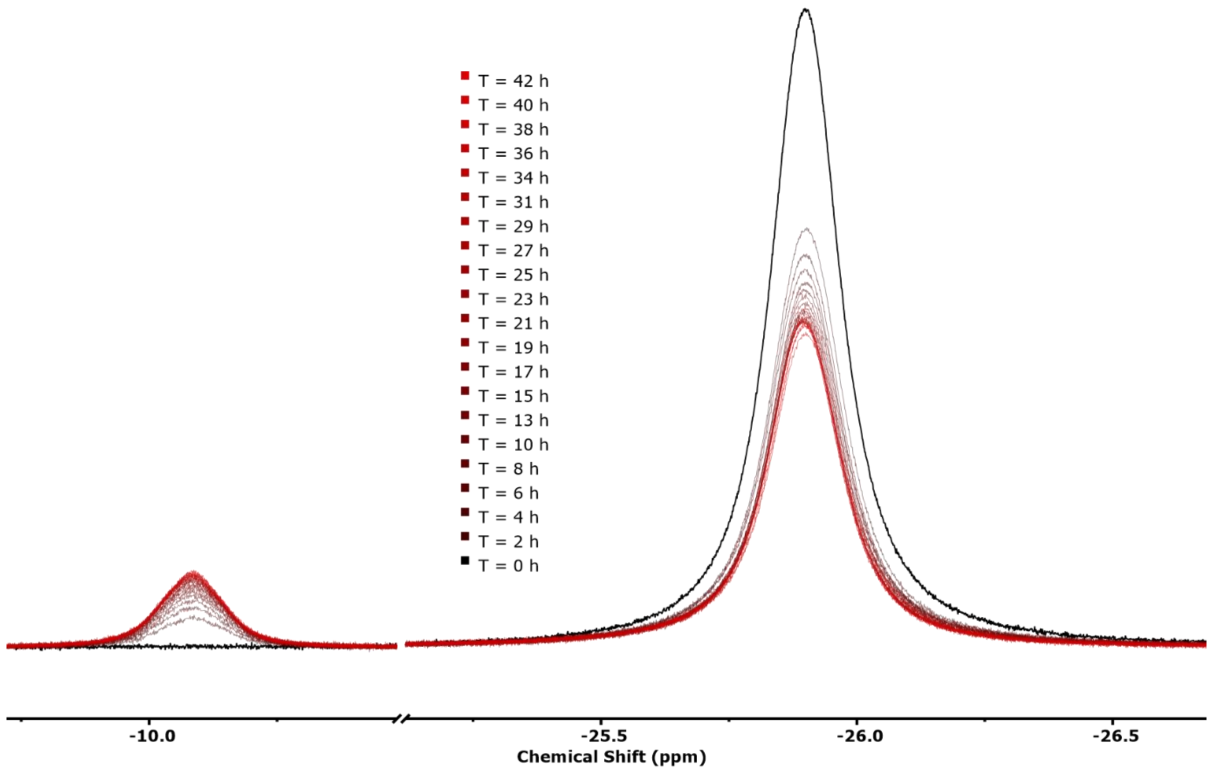

Figure S17. Overlaid ^{1}H NMR spectra of Reaction 1 over 42 h, enlarging signals at *ca.* -10.2 and -25.9 ppm.

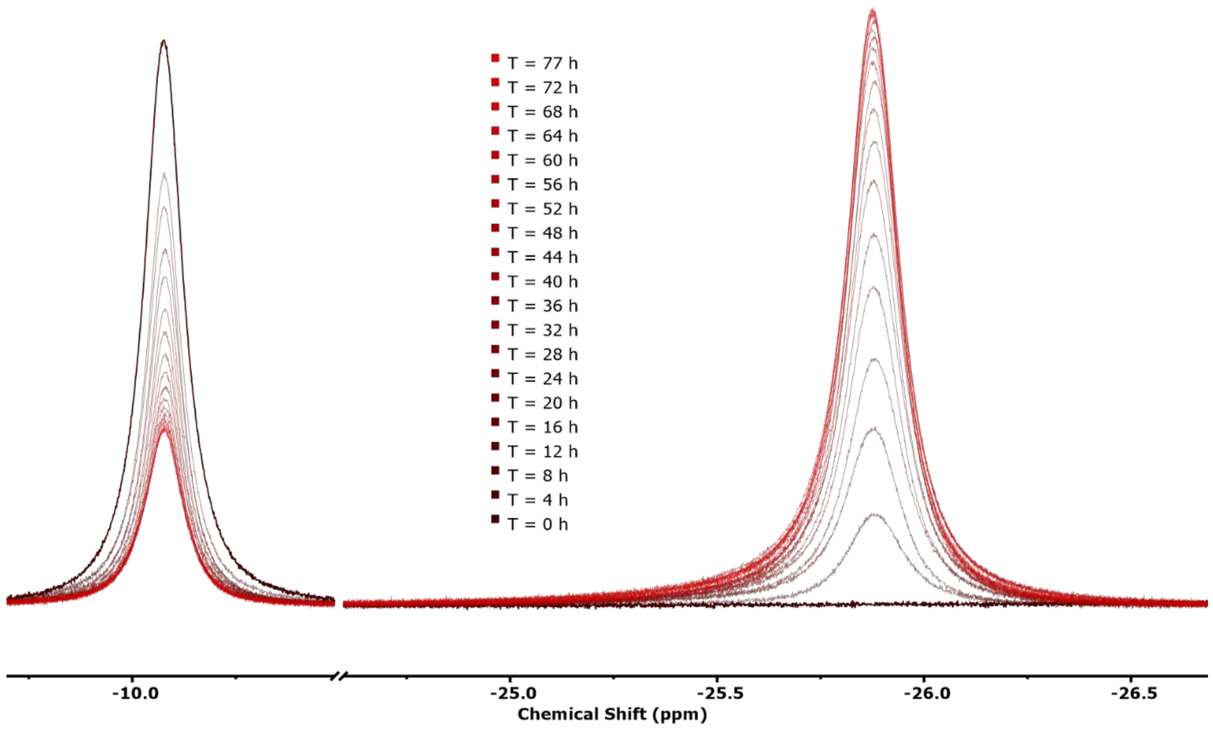

Figure S18. Overlaid ^{1}H NMR spectra of Reaction 2 over 77 h, enlarging signals at *ca.* -10.2 and -25.9 ppm.

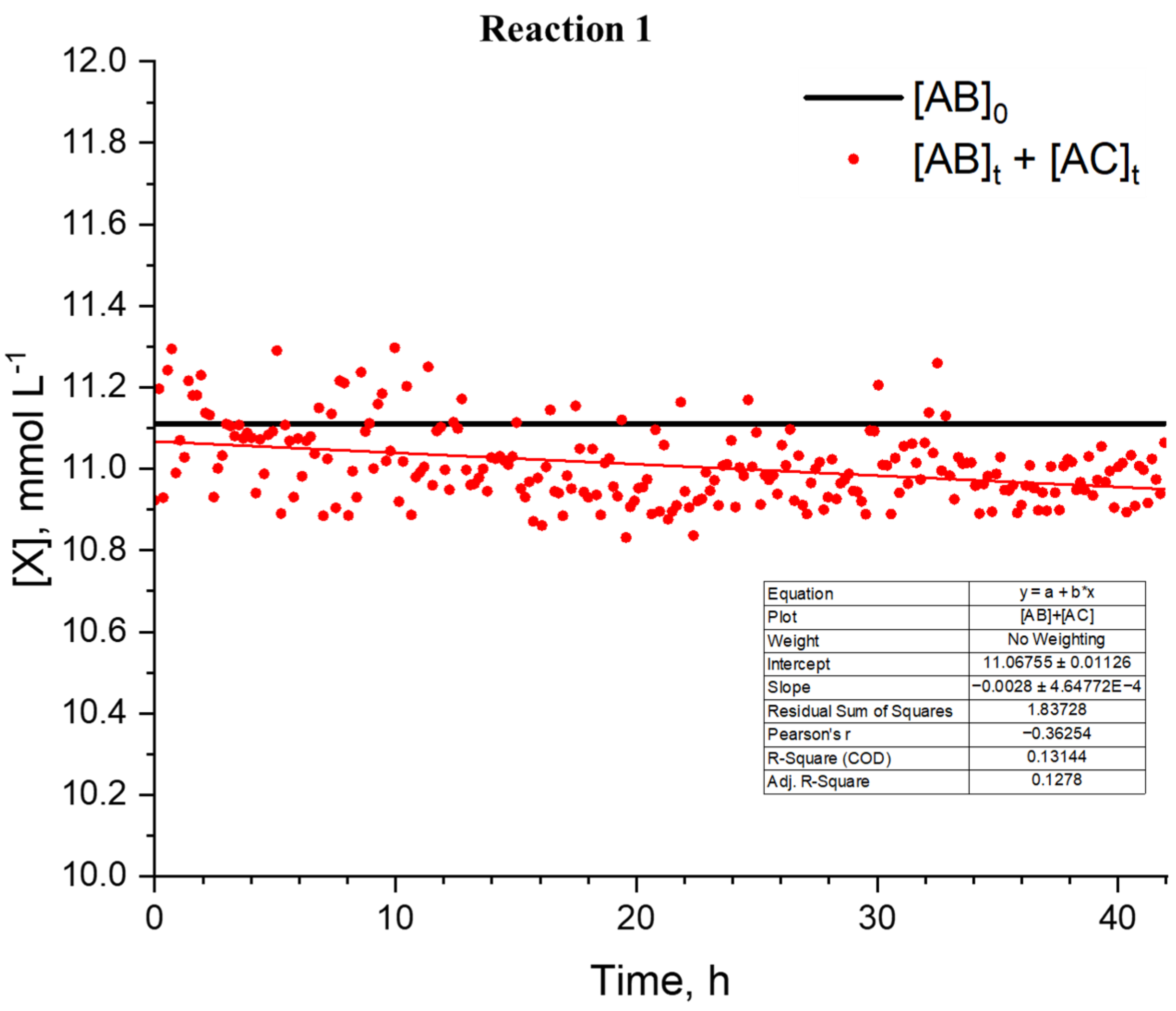

Figure S19. Plot of $[AB]_t + [AC]_t$ vs time for Reaction 1.

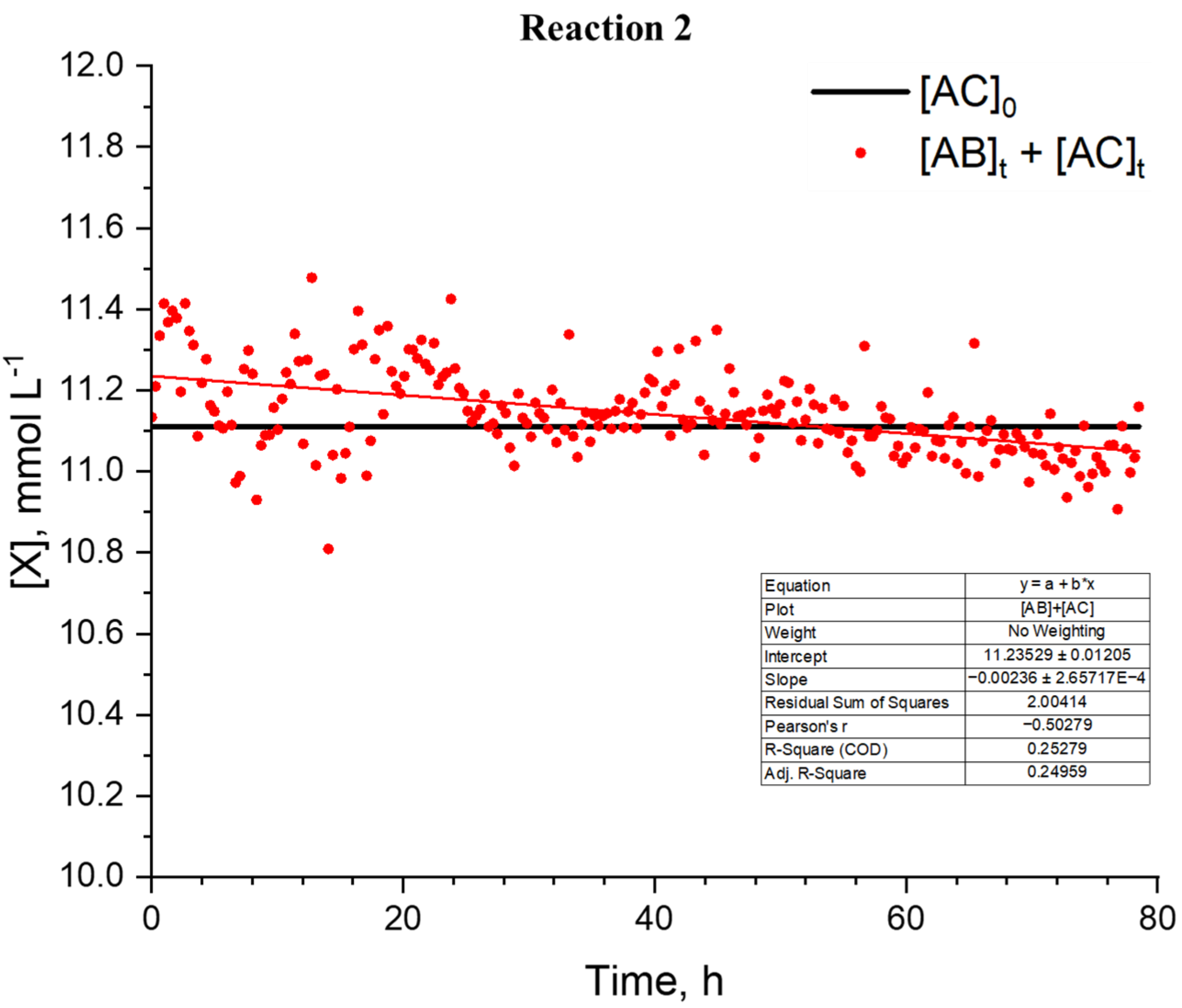

Figure S20. Plot of $[AB]_t + [AC]_t$ vs time for Reaction 2.

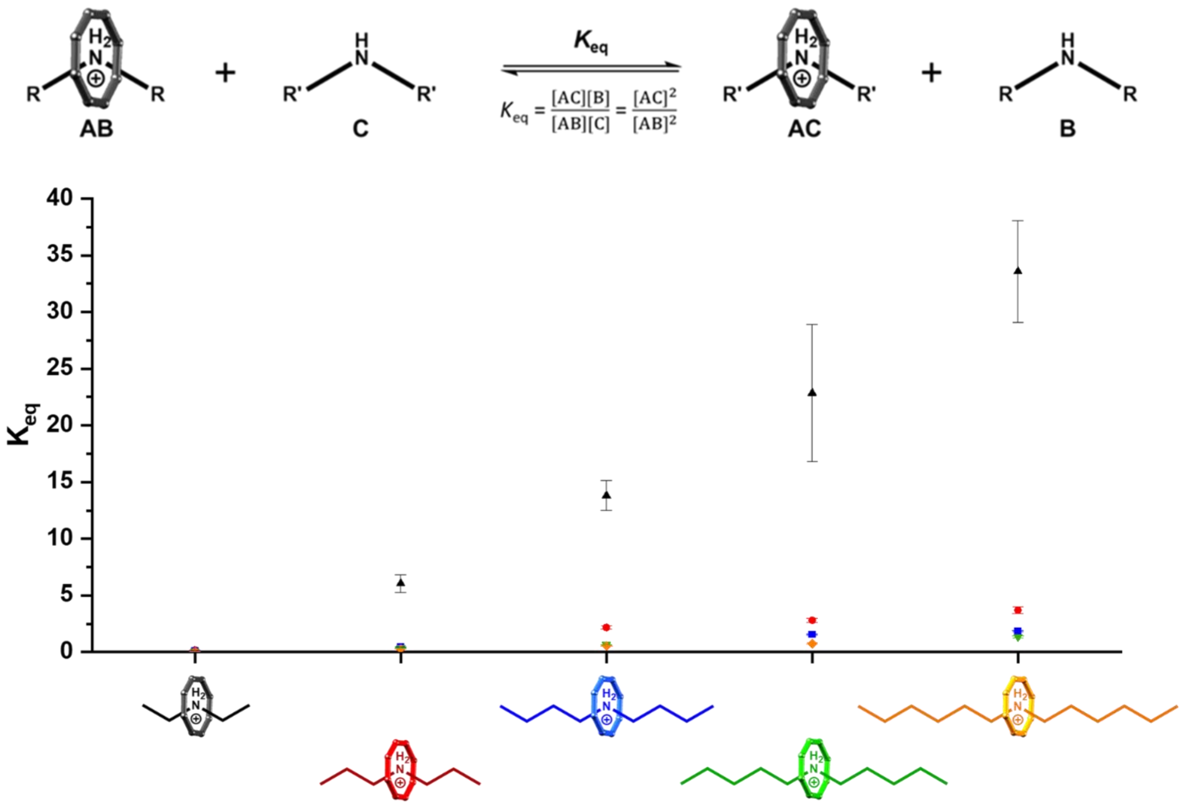

Figure S21. Thermodynamic plot, K_{eq} vs initial pseudorotaxane species (AB) in toluene-d_8 with corresponding error bars. Where equilibrium constant for the product pseudorotaxane (AC) is indicated by: black triangles (**Et_2NH_2·1**) red circles (**Pr_2NH_2·1**), blue squares (**Bu_2NH_2·1**), green triangles (**Pe_2NH_2·1**) and orange diamonds (**Hx_2NH_2·1**). NMR experiments conducted at 298 K with $[AB]_0$ and $[C]_0$ = 30 mM.

Table S2. Equilibrium constants (K_{eq}) for thread exchange reactions in toluene-d_8 with associated standard deviations (calculated for 298 K).

		K_{eq}				
	C =	Diethylamine	Dipropylamine	Dibutylamine	Dipentylamine	Dihexylamine
AB	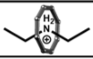		0.16 ± 0.0087	0.073 ± 0.0059	0.042 ± 0.011	0.030 ± 0.0042
	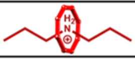	5.9 ± 1.0		0.47 ± 0.021	0.36 ± 0.028	0.27 ± 0.020
	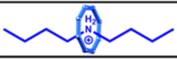	14 ± 1.5	2.2 ± 0.16		0.65 ± 0.022	0.54 ± 0.019
	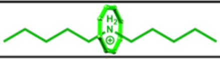	22 ± 6.0	2.8 ± 0.14	1.5 ± 0.047		0.75 ± 0.093
	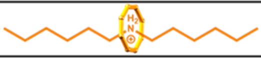	34 ± 4.4	3.7 ± 0.30	1.8 ± 0.034	1.3 ± 0.061	

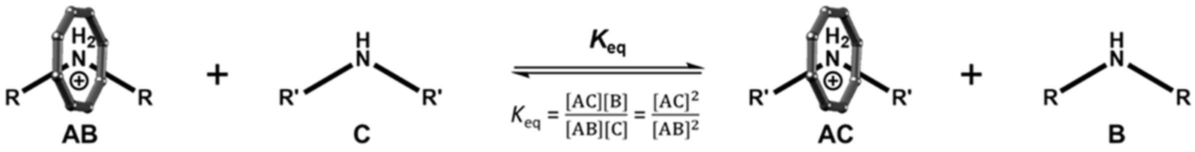

Table S3. Gibbs free energies (ΔG) for thread exchange reactions in toluene-d_8 with associated standard deviations (calculated for 298 K).

		ΔG, kJ mol^{-1}				
	C =	Diethylamine	Dipropylamine	Dibutylamine	Dipentylamine	Dihexylamine
AB	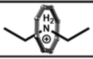		4.5 ± 0.13	6.5 ± 0.27	7.9 ± 0.62	8.7 ± 0.34
	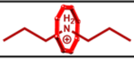	-4.4 ± 0.43		1.9 ± 0.11	2.5 ± 0.19	3.2 ± 0.18
	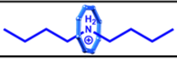	-6.5 ± 0.27	-2.0 ± 0.18		1.1 ± 0.085	1.5 ± 0.086
	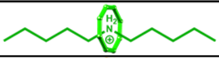	-7.6 ± 0.68	-2.6 ± 0.12	-1.1 ± 0.075		0.71 ± 0.10
	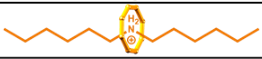	-8.7 ± 0.32	-3.2 ± 0.20	-1.5 ± 0.046	-0.73 ± 0.17	

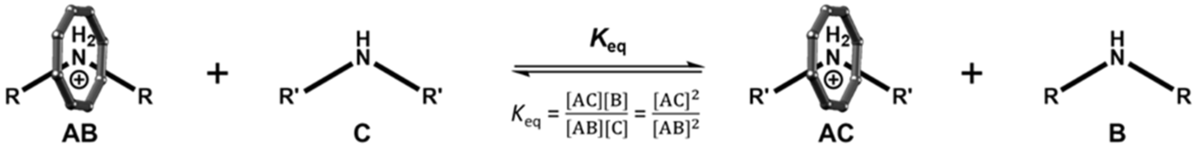

Table S4. Equilibrium constants (K_{eq}) for thread exchange reactions in acetone-d_6 with associated standard deviations (calculated for 298 K).

		K_{eq}									
	C =	Diethylamine	Ethylpropylamine	Dibutylamine	Dipentylamine	Dihexylamine	Methylpropylamine	Ethylmethylamine	Diallylamine	Dioctylamine	Dipropylamine
AB	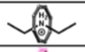										0.27 ± 0.017
	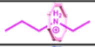										0.69 ± 0.044
	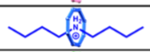										1.3 ± 0.030
	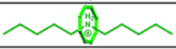										1.5 ± 0.071
	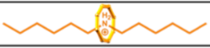										1.2 ± 0.11
	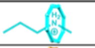										1.3 ± 0.038
	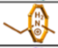										1.2 ± 0.097
	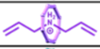										200 ± 26
	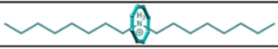										0.63 ± 0.065
	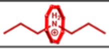	4.0 ± 0.19	1.5 ± 0.074	0.79 ± 0.027	0.70 ± 0.017	0.86 ± 0.047	0.77 ± 0.019	0.82 ± 0.042	0.0058 ± 0.00086	1.5 ± 0.25	

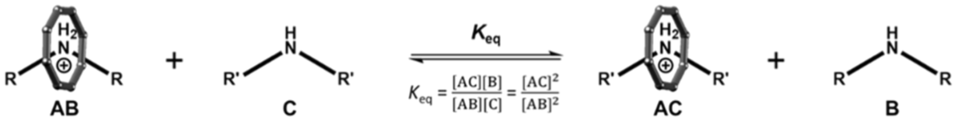

Table S5. Gibbs free energies (ΔG) for thread exchange reactions in acetone-d_6 with associated standard deviations (calculated for 298 K).

		ΔG, kJ mol^{-1}									
	C =	Diethylamine	Ethylpropylamine	Dibutylamine	Dipentylamine	Dihexylamine	Methylpropylamine	Ethylmethylamine	Diallylamine	Dioctylamine	Dipropylamine
AB	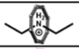										3.3 ± 0.16
	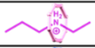										0.93 ± 0.16
	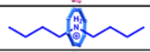										-0.57 ± 0.059
	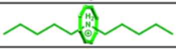										-1.0 ± 0.12
	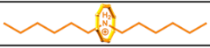										-0.43 ± 0.22
	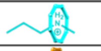										-0.59 ± 0.074
	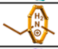										-0.53 ± 0.19
	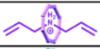										-13 ± 0.32
	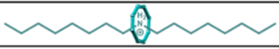										1.1 ± 0.26
	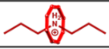	-3.4 ± 0.12	-0.97 ± 0.12	0.60 ± 0.086	0.87 ± 0.061	0.38 ± 0.14	0.64 ± 0.062	0.50 ± 0.13	13 ± 0.37	-1.1 ± 0.42	

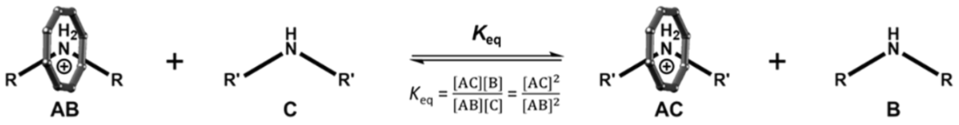

Alternative error analysis

Standard deviations in Tables S2 – S5 have been calculated from two reactions each giving rise to three ^{1}H NMR spectra which are manually baseline corrected three times each giving rise to a total of 18 data points.

As there are only a total of four reactions for each equilibrium, a more generous error estimation has been used for Figures S22 – S23 & Tables S6 – S9. Here, the range (difference between maximum and minimum concentrations) calculated from 36 data points (4 reactions × 3 time points × 3 manual baseline corrections) are used to estimate the error.

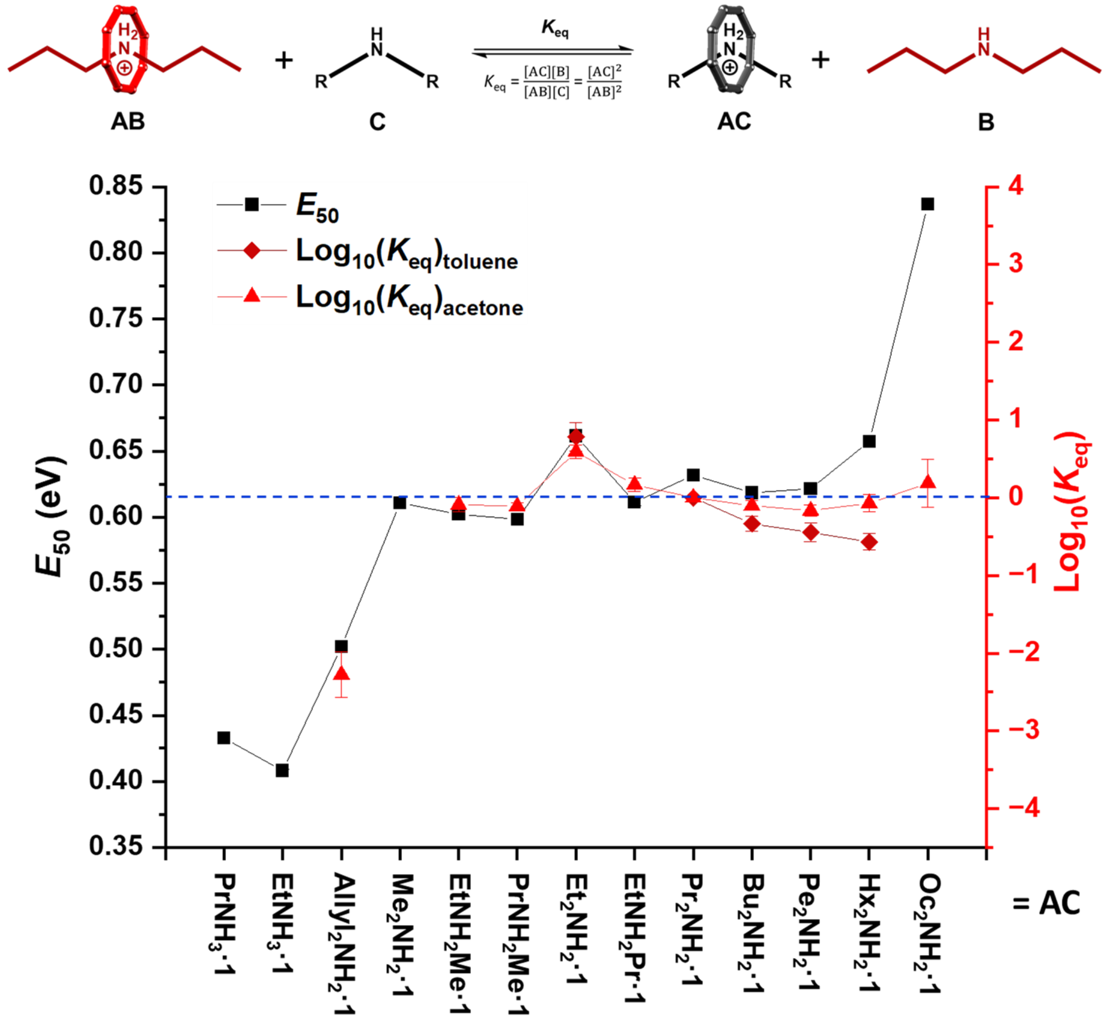

Figure S22. Dual axis plot showing: E_{50} values for the dissociation of the ammonium cations from {Cr_7Co} rings (black squares) measured using CID-MS; and log K_{eq} vs final pseudorotaxane species (AC) measured using NMR for exchange reactions in acetone-d_6 (red triangles) and toluene-d_8 (red diamonds), where AB = **$Pr_2NH_2{\cdot}1$**. Error bars are shown and are smaller than the symbol size. NMR experiments conducted at 298 K with $[AB]_0$ and $[C]_0$ = 30 mM.

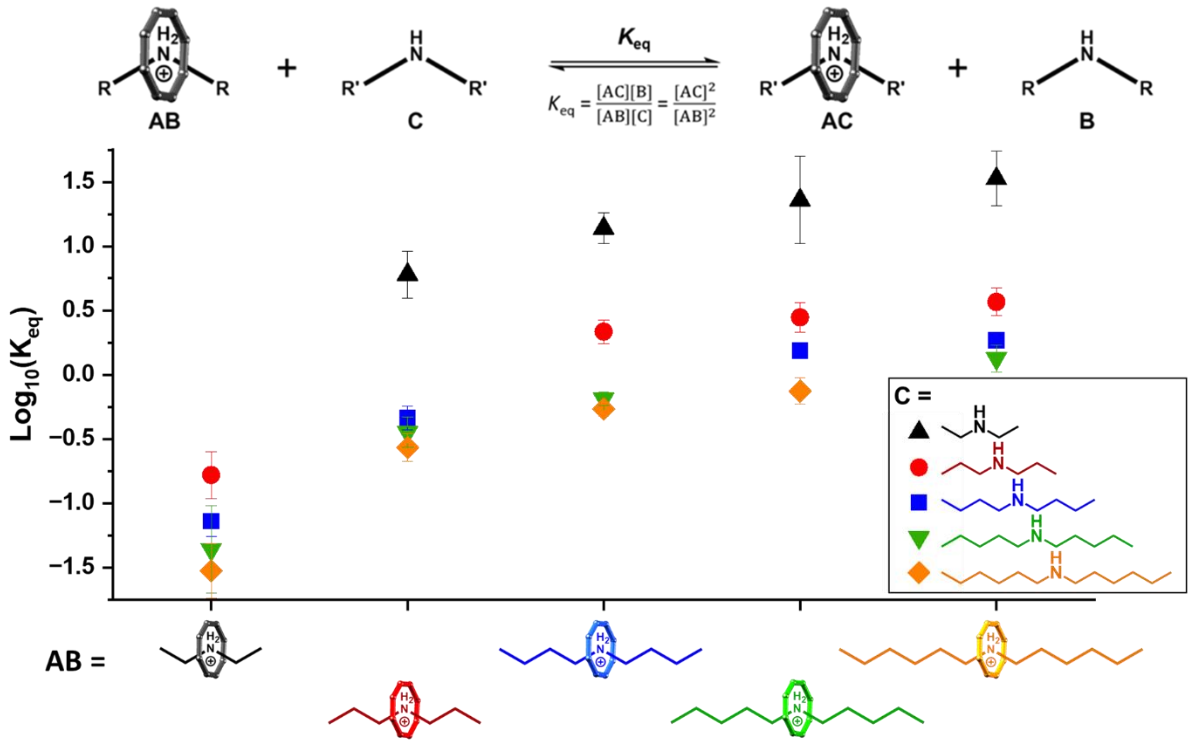

Figure S23. Thermodynamic plot: log K_{eq} *vs.* initial pseudorotaxane species (AB) in toluene-d_8 with corresponding error bars. Log K_{eq} for the equilibrium with the different product pseudorotaxanes (AC) is indicated by: black triangles (**Et_2NH_2·1**), red circles (**Pr_2NH_2·1**), blue squares (**Bu_2NH_2·1**), green triangles (**Pe_2NH_2·1**) and orange diamonds (**Hx_2NH_2·1**). Error bars are shown and are smaller than the symbol size. NMR experiments conducted at 298 K with $[AB]_0$ and $[C]_0$ = 30 mM.

Table S6. Equilibrium constants (K_{eq}) for thread exchange reactions in toluene-d_8 with associated standard deviations (calculated for 298 K).

		K_{eq}				
	**C =**	Diethylamine	Dipropylamine	Dibutylamine	Dipentylamine	Dihexylamine
**AB**	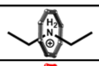		0.16 ± 0.068	0.073 ± 0.020	0.042 ± 0.033	0.030 ± 0.015
	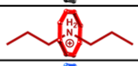	5.9 ± 2.4		0.47 ± 0.10	0.36 ± 0.097	0.27 ± 0.067
	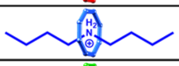	14 ± 3.9	2.2 ± 0.48		0.65 ± 0.076	0.54 ± 0.067
	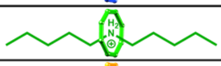	22 ± 17	2.8 ± 0.76	1.5 ± 0.18		0.75 ± 0.18
	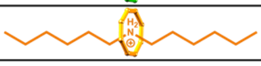	34 ± 17	3.7 ± 0.92	1.8 ± 0.23	1.3 ± 0.32	

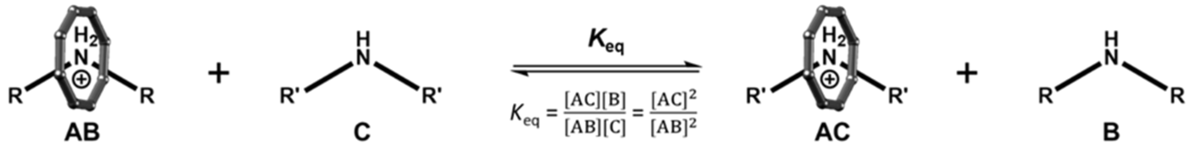

Table S7. Gibbs free energies (ΔG) for thread exchange reactions in toluene-d_8 with associated standard deviations (calculated for 298 K).

		ΔG, kJ mol^{-1}				
	C =	Diethylamine	Dipropylamine	Dibutylamine	Dipentylamine	Dihexylamine
AB	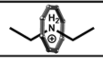		4.5 ± 1.1	6.5 ± 0.69	7.9 ± 2.0	8.7 ± 1.2
	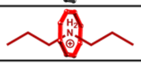	-4.4 ± 1.0		1.9 ± 0.53	2.5 ± 0.67	3.2 ± 0.62
	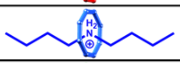	-6.5 ± 0.69	-2.0 ± 0.53		1.1 ± 0.29	1.5 ± 0.31
	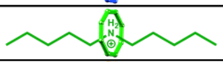	-7.6 ± 1.9	-2.6 ± 0.67	-1.1 ± 0.29		0.71 ± 0.59
	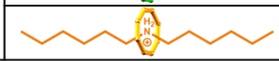	-8.7 ± 1.2	-3.2 ± 0.62	-1.5 ± 0.31	-0.73 ± 0.60	

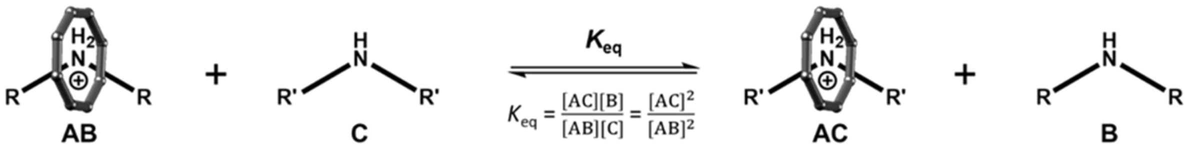

Table S8. Equilibrium constants (K_{eq}) for thread exchange reactions in acetone-d_6 with associated standard deviations (calculated for 298 K).

		K_{eq}									
	C =	Diethylamine	Ethylpropylamine	Dibutylamine	Dipentylamine	Dihexylamine	Methylpropylamine	Ethylmethylamine	Diallylamine	Dioctylamine	Dipropylamine
AB	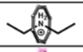										0.27 ± 0.052
	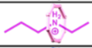										0.69 ± 0.13
	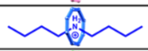										1.3 ± 0.18
	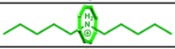										1.5 ± 0.12
	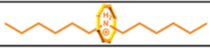										1.2 ± 0.30
	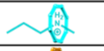										1.3 ± 0.12
	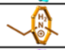										1.2 ± 0.26
	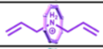										200 ± 140
	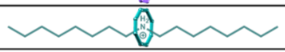										0.63 ± 0.45
	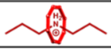	4.0 ± 0.80	1.5 ± 0.80	0.79 ± 0.11	0.70 ± 0.12	0.86 ± 0.22	0.77 ± 0.076	0.82 ± 0.17	0.0058 ± 0.0037	1.5 ± 1.1	

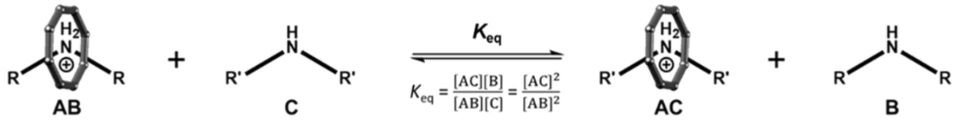

Table S9. Gibbs free energies (ΔG) for thread exchange reactions in acetone-d_6 with associated standard deviations (calculated for 298 K).

		ΔG, kJ mol^{-1}									
	C =	Diethylamine	Ethylpropylamine	Dibutylamine	Dipentylamine	Dihexylamine	Methylpropylamine	Ethylmethylamine	Diallylamine	Dioctylamine	Dipropylamine
AB	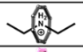										3.3 ± 0.49
	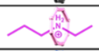										0.93 ± 0.48
	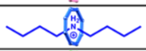										-0.57 ± 0.36
	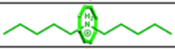										-1.0 ± 0.41
	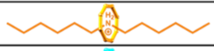										-0.43 ± 0.64
	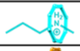										-0.59 ± 0.24
	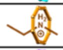										-0.54 ± 0.52
	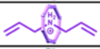										-13 ± 1.7
	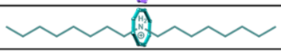										1.1 ± 1.8
	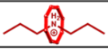	-3.4 ± 0.50	-0.97 ± 1.3	0.60 ± 0.36	0.87 ± 0.41	0.38 ± 0.64	0.64 ± 0.24	0.50 ± 0.52	13 ± 1.6	-1.0 ± 1.8	

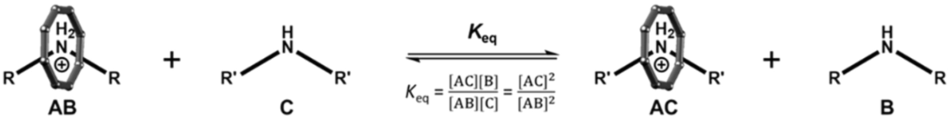

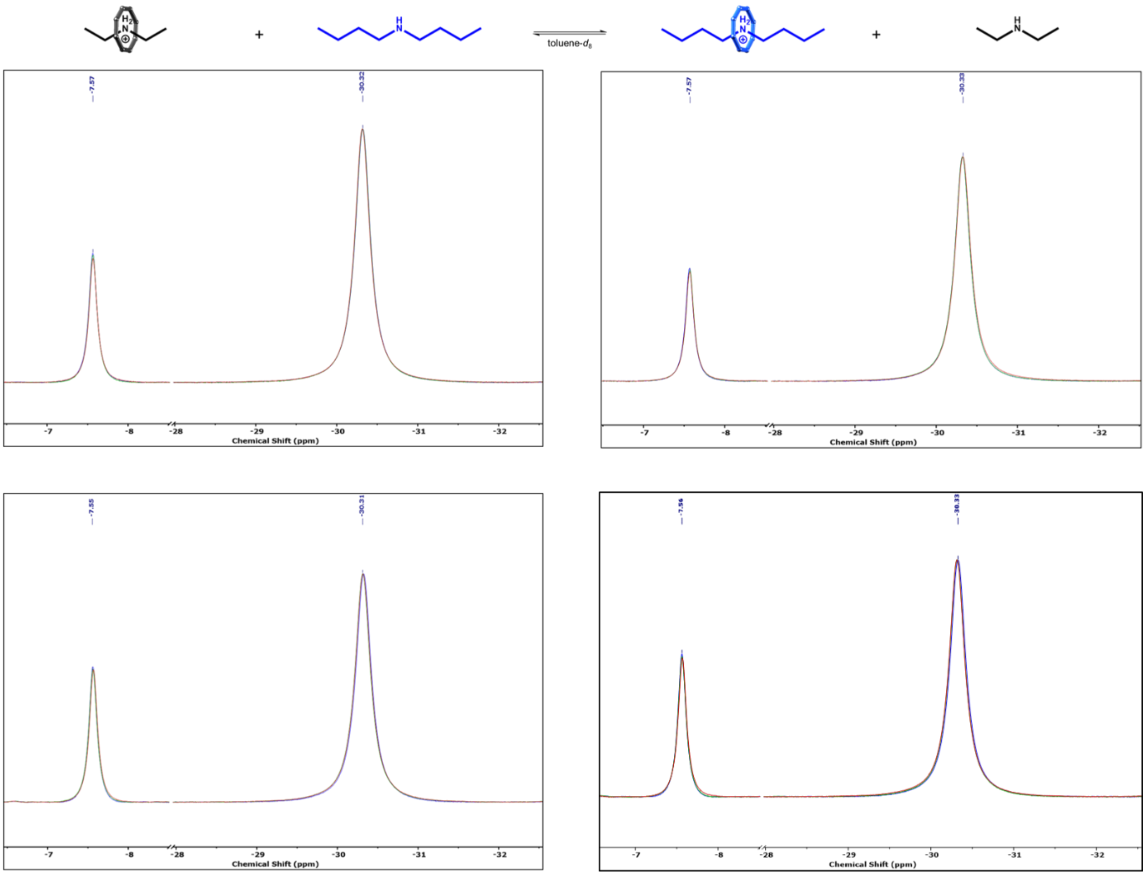

Figure S24. Overlaid normalised ^{1}H NMR spectra (500 MHz, 298 K) showing the signals used for quantification of the concentration of **Et_2NH_2·1** and **Bu_2NH_2·1** at equilibrium in toluene-d_8 at three time intervals (red, green and blue) separated by ≈ 100 h. The forwards (top left) and reverse (bottom left) equilibration reactions have been displayed alongside a repeat (top right and bottom right respectively).

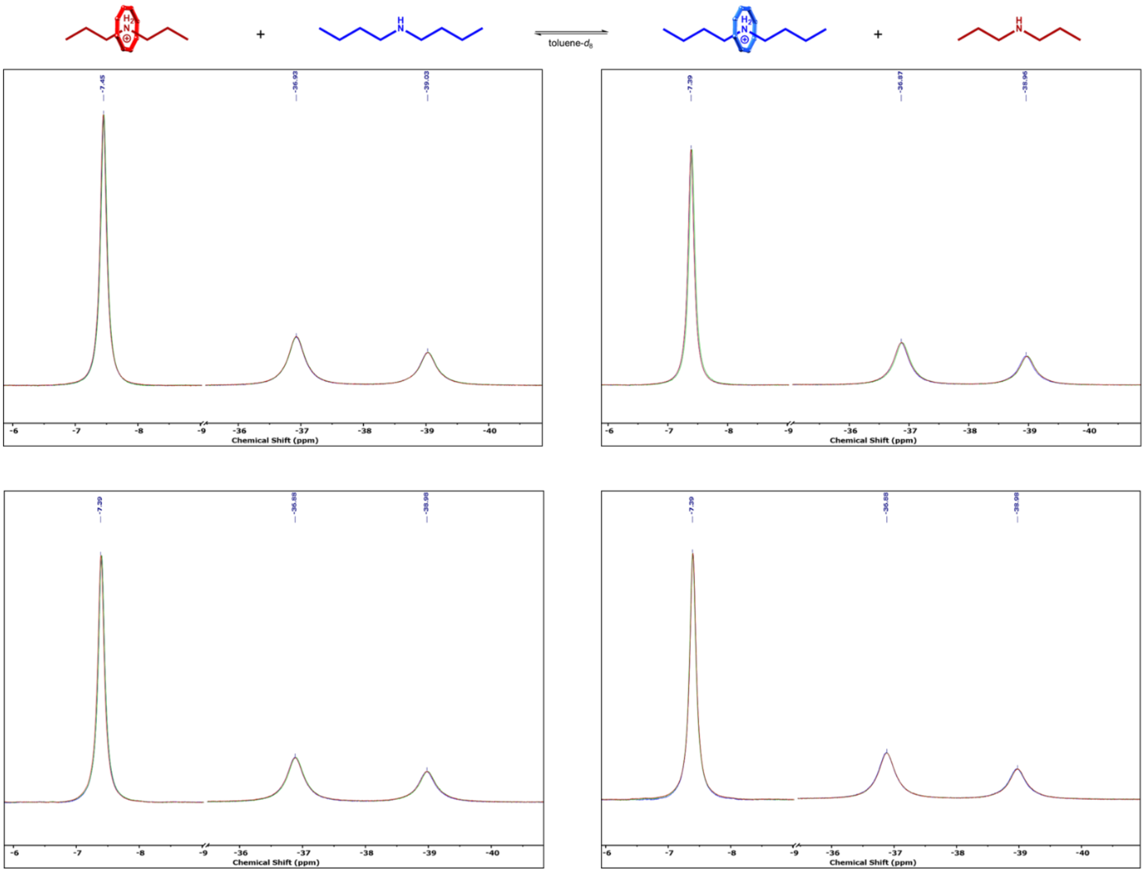

Figure S25. Overlaid normalised ^{1}H NMR spectra (500 MHz, 298 K) showing the signals used for quantification of the concentration of **Pr_2NH_2·1** and **Bu_2NH_2·1** at equilibrium in toluene-d_8 at three time intervals (red, green and blue) separated by ≈ 100 h. The forwards (top left) and reverse (bottom left) equilibration reactions have been displayed alongside a repeat (top right and bottom right respectively).

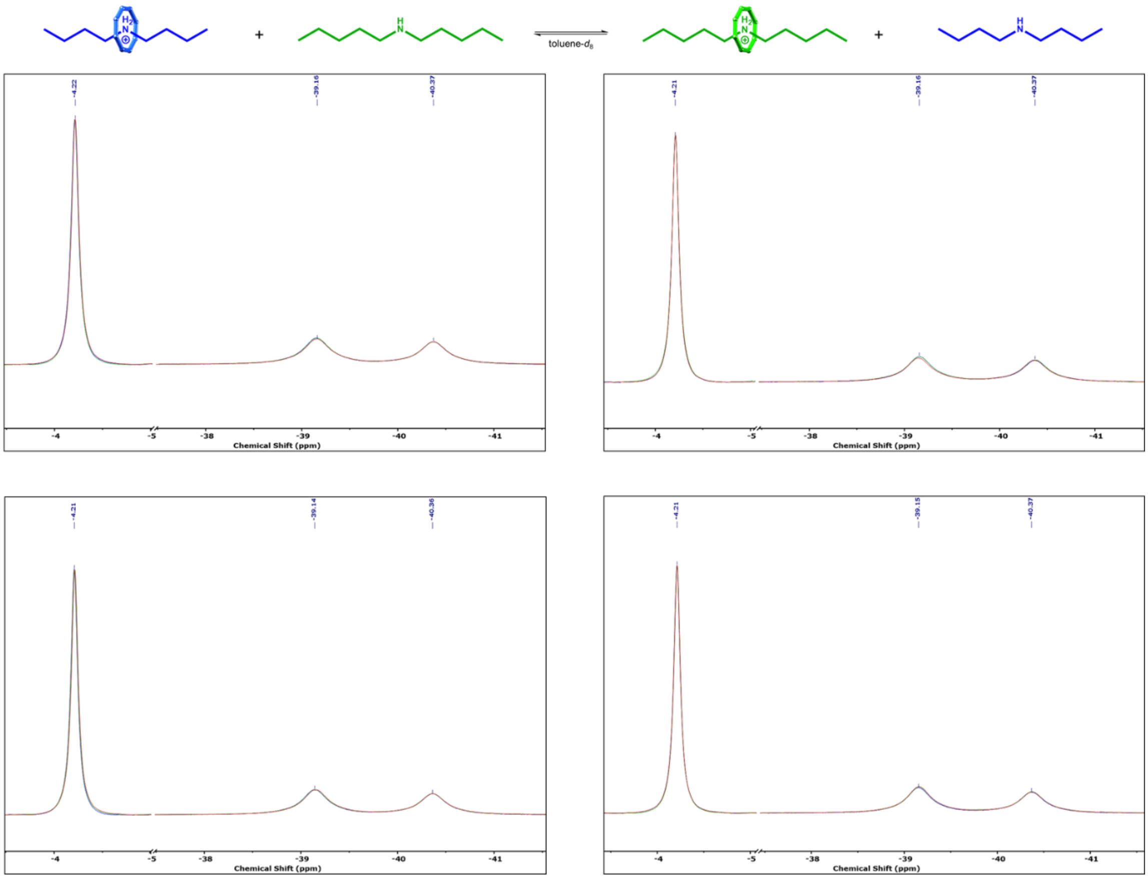

Figure S26. Overlaid normalised ^{1}H NMR spectra (500 MHz, 298 K) showing the signals used for quantification of the concentration of **Bu_2NH_2·1** and **Pe_2NH_2·1** at equilibrium in toluene-d_8 at three time intervals (red, green and blue) separated by ≈ 100 h. The forwards (top left) and reverse (bottom left) equilibration reactions have been displayed alongside a repeat (top right and bottom right respectively).

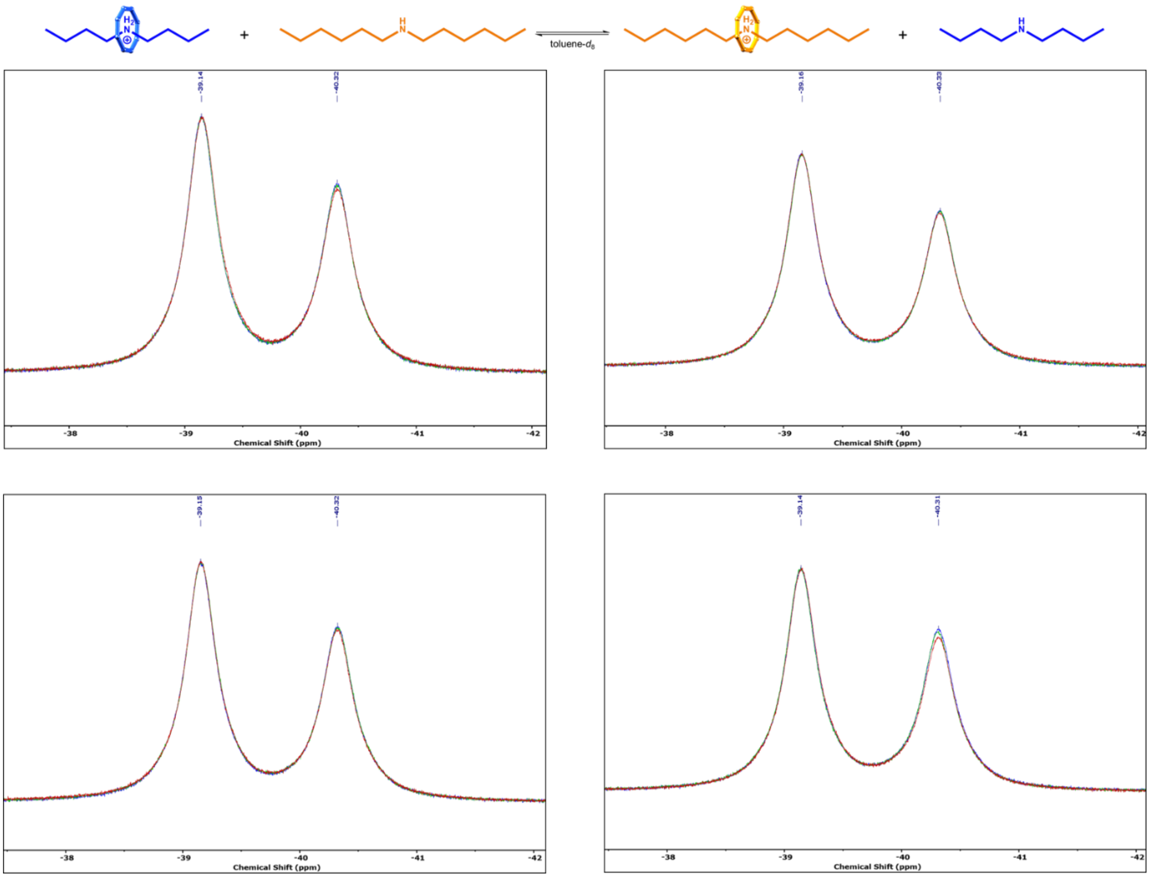

Figure S27. Overlaid normalised ^{1}H NMR spectra (500 MHz, 298 K) showing the signals used for quantification of the concentration of **Bu_2NH_2·1** and **Hx_2NH_2·1** at equilibrium in toluene-d_8 at three time intervals (red, green and blue) separated by ≈ 100 h. The forwards (top left) and reverse (bottom left) equilibration reactions have been displayed alongside a repeat (top right and bottom right respectively).

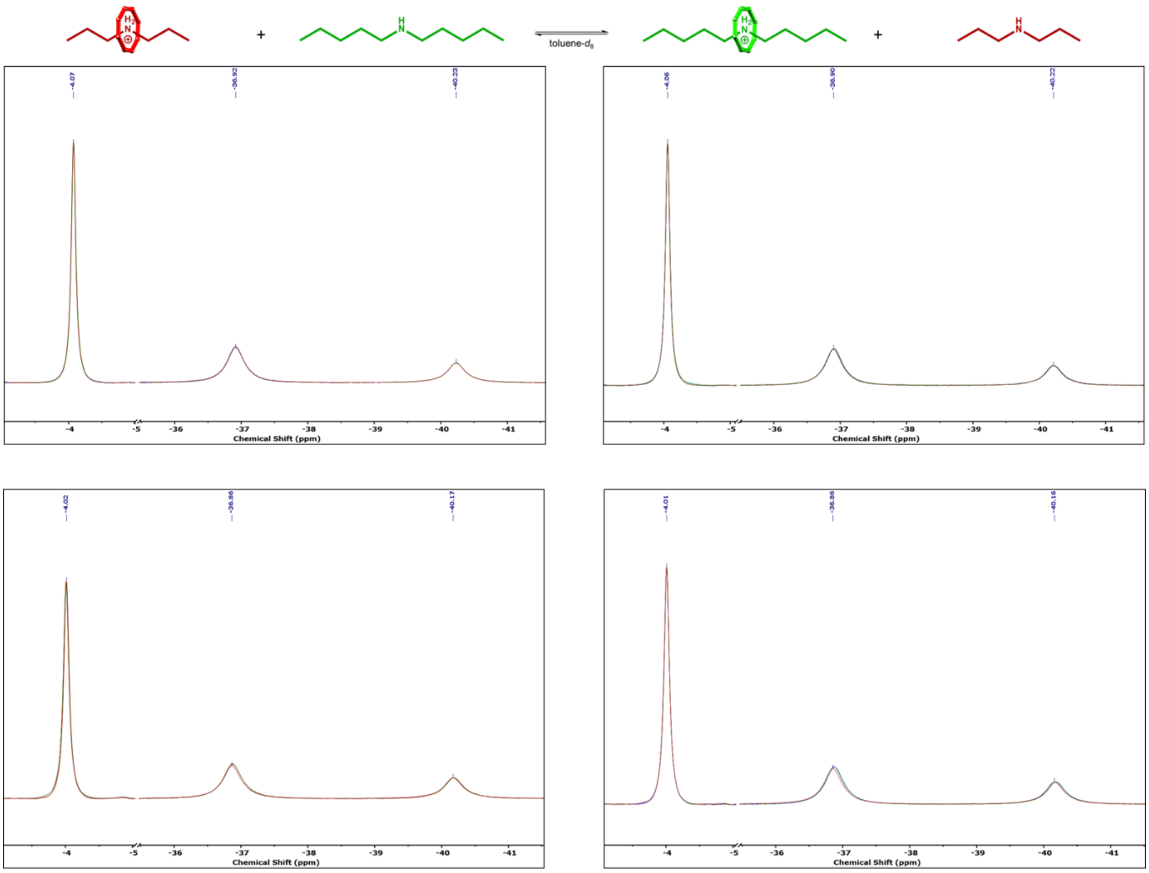

Figure S28. Overlaid normalised ^{1}H NMR spectra (500 MHz, 298 K) showing the signals used for quantification of the concentration of **Pr_2NH_2·1** and **Pe_2NH_2·1** at equilibrium in toluene-d_8 at three time intervals (red, green and blue) separated by ≈ 100 h. The forwards (top left) and reverse (bottom left) equilibration reactions have been displayed alongside a repeat (top right and bottom right respectively).

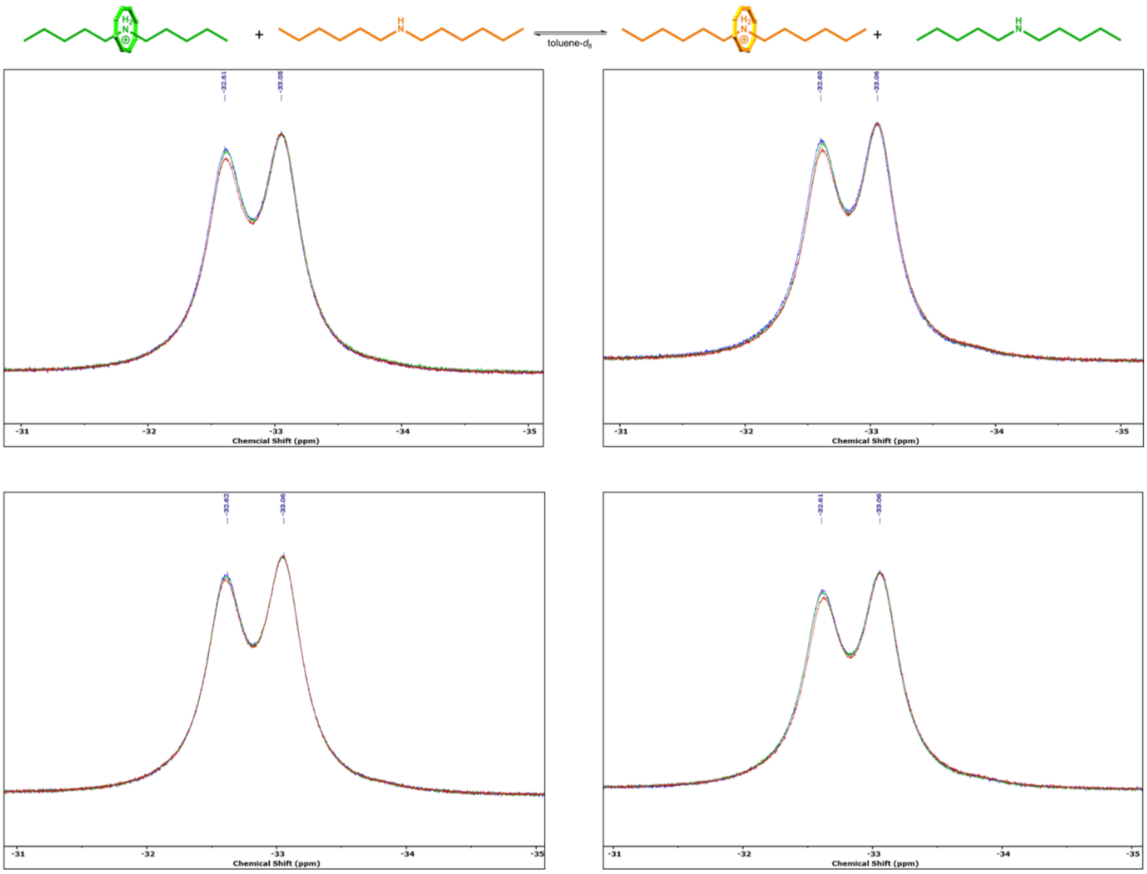

Figure S29. Overlaid normalised ^{1}H NMR spectra (500 MHz, 298 K) showing the signals used for quantification of the concentration of **Pe_2NH_2·1** and **Hx_2NH_2·1** at equilibrium in toluene-d_8 at three time intervals (red, green and blue) separated by ≈ 100 h. The forwards (top left) and reverse (bottom left) equilibration reactions have been displayed alongside a repeat (top right and bottom right respectively).

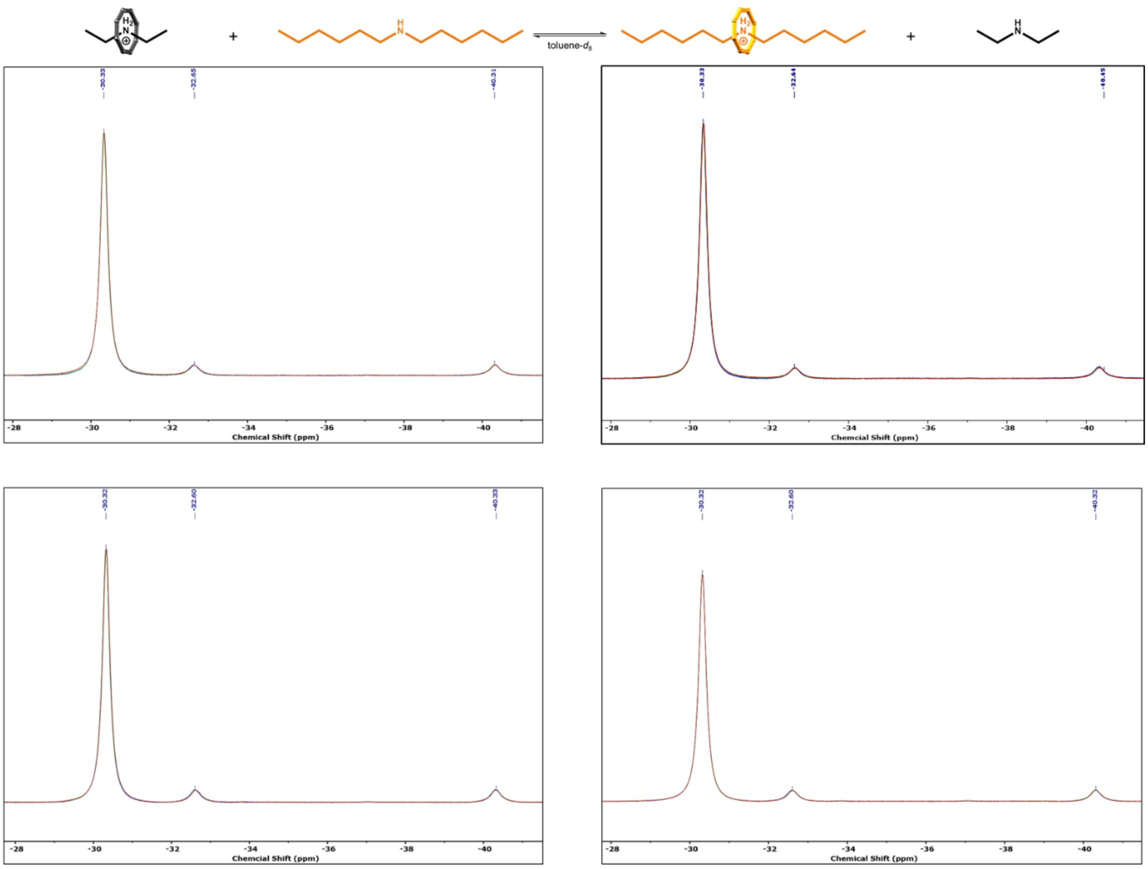

Figure S30. Overlaid normalised ^{1}H NMR spectra (500 MHz, 298 K) showing the signals used for quantification of the concentration of $\mathbf{Et_2NH_2{\cdot}1}$ and $\mathbf{Hx_2NH_2{\cdot}1}$ at equilibrium in toluene-d_8 at three time intervals (red, green and blue) separated by ≈ 100 h. The forwards (top left) and reverse (bottom left) equilibration reactions have been displayed alongside a repeat (top right and bottom right respectively).

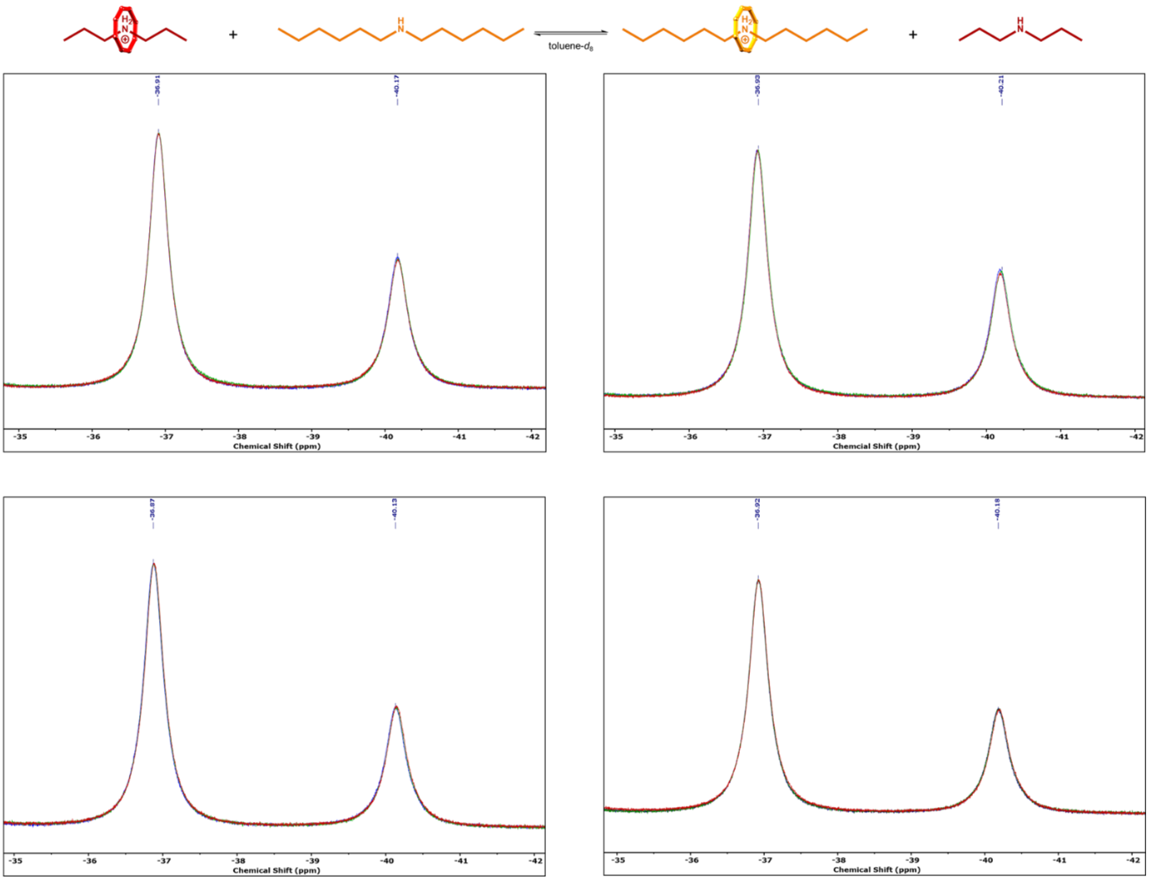

Figure S31. Overlaid normalised ^{1}H NMR spectra (500 MHz, 298 K) showing the signals used for quantification of the concentration of **Pr_2NH_2·1** and **Hx_2NH_2·1** at equilibrium in toluene-d_8 at three time intervals (red, green and blue) separated by ≈ 100 h. The forwards (top left) and reverse (bottom left) equilibration reactions have been displayed alongside a repeat (top right and bottom right respectively).

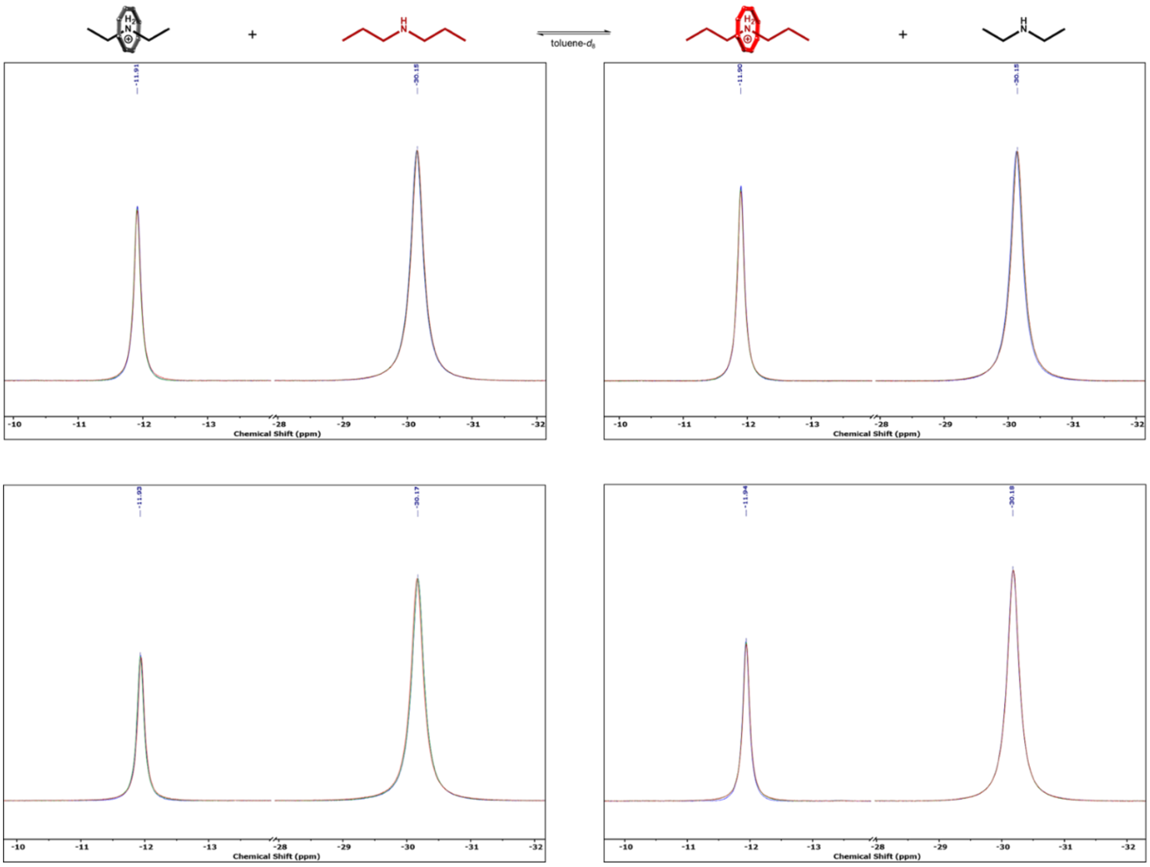

Figure S32. Overlaid normalised ^{1}H NMR spectra (500 MHz, 298 K) showing the signals used for quantification of the concentration of **Et_2NH_2·1** and **Pr_2NH_2·1** at equilibrium in toluene-d_8 at three time intervals (red, green and blue) separated by ≈ 100 h. The forwards (top left) and reverse (bottom left) equilibration reactions have been displayed alongside a repeat (top right and bottom right respectively).

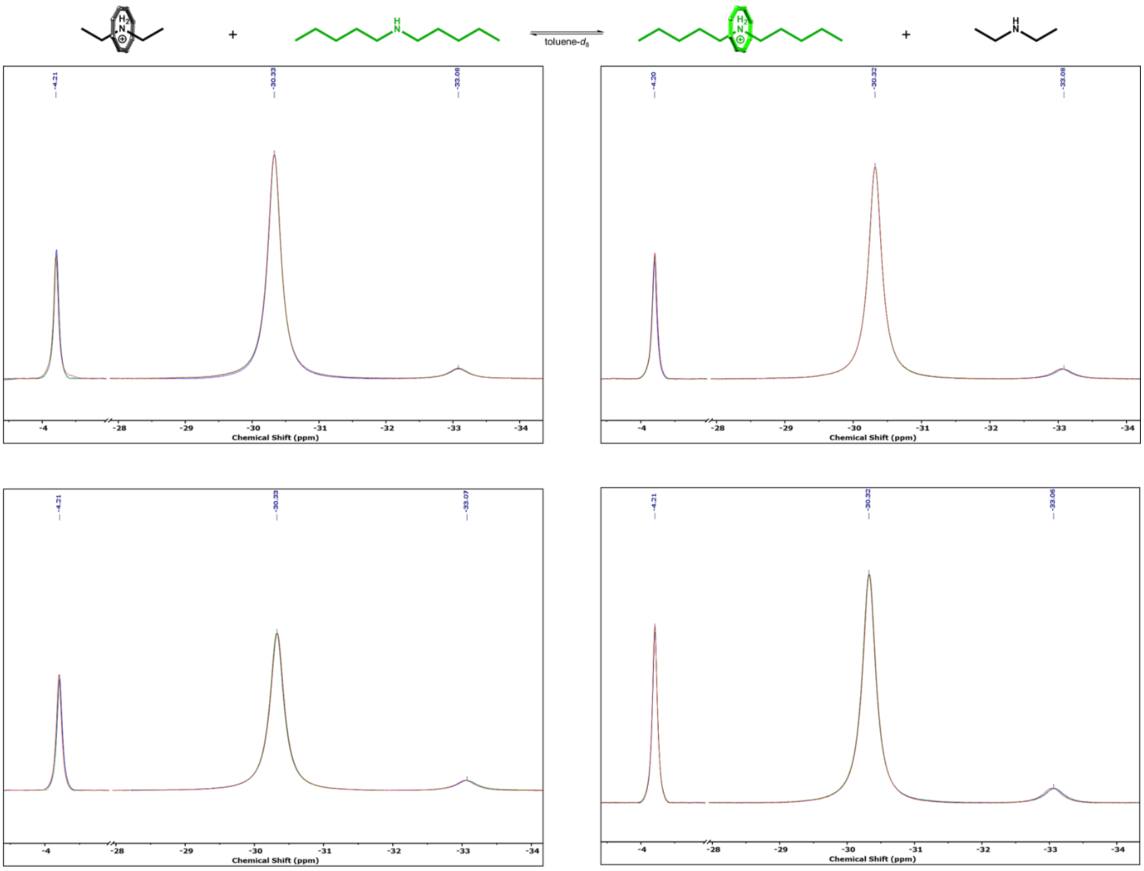

Figure S33. Overlaid normalised ^{1}H NMR spectra (500 MHz, 298 K) showing the signals used for quantification of the concentration of **Et_2NH_2·1** and **Pe_2NH_2·1** at equilibrium in toluene-d_8 at three time intervals (red, green and blue) separated by ≈ 100 h. The forwards (top left) and reverse (bottom left) equilibration reactions have been displayed alongside a repeat (top right and bottom right respectively).

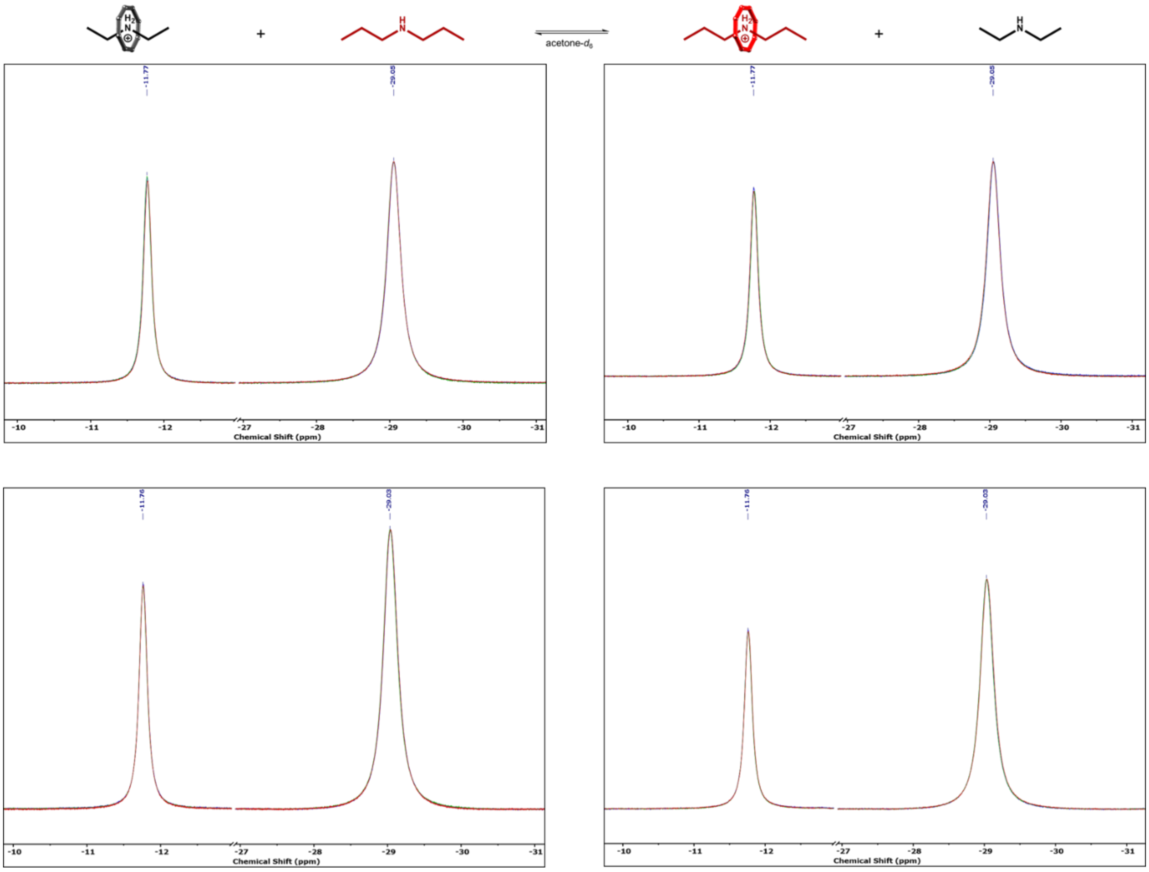

Figure S34. Overlaid normalised ^{1}H NMR spectra (500 MHz, 298 K) showing the signals used for quantification of the concentration of **Et_2NH_2·1** and **Pr_2NH_2·1** at equilibrium in acetone-d_6 at three time intervals (red, green and blue) separated by ≈ 12 h. The forwards (top left) and reverse (bottom left) equilibration reactions have been displayed alongside a repeat (top right and bottom right respectively).

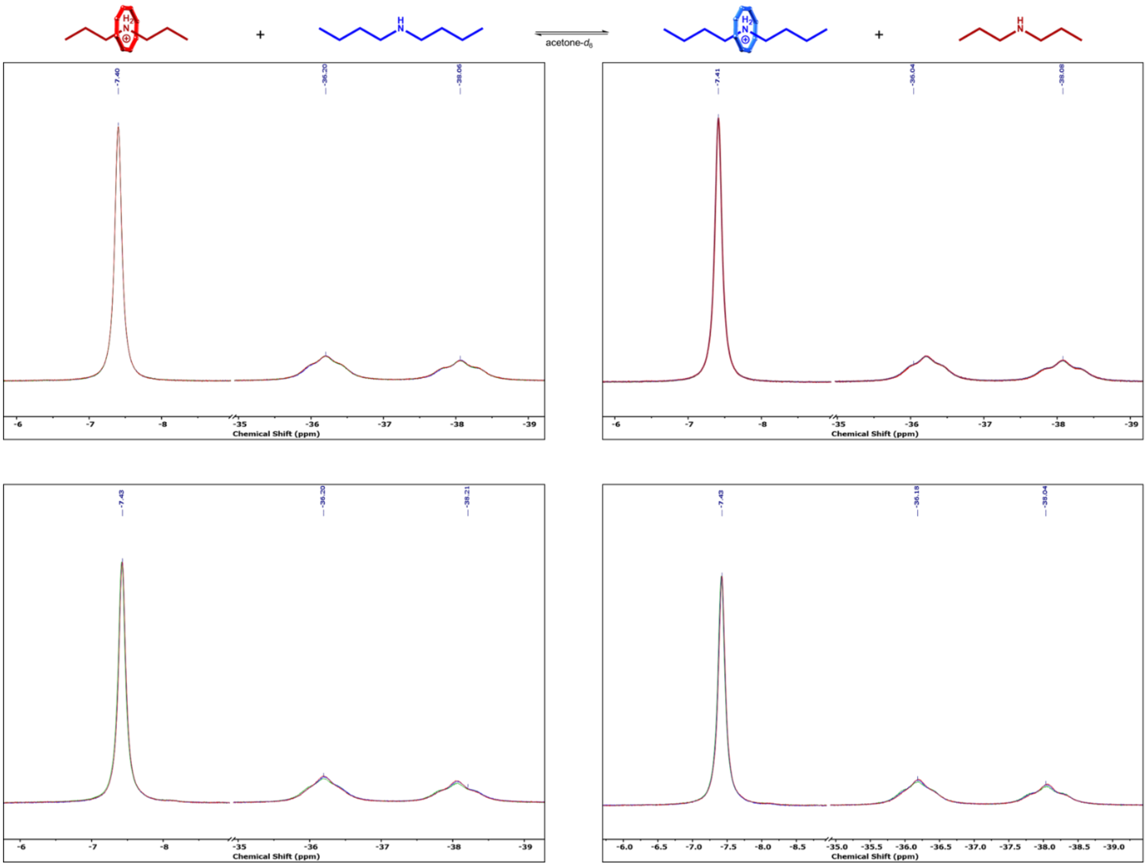

Figure S35. Overlaid normalised ^{1}H NMR spectra (500 MHz, 298 K) showing the signals used for quantification of the concentration of **Pr_2NH_2·1** and **Bu_2NH_2·1** at equilibrium in acetone-d_6 at three time intervals (red, green and blue) separated by ≈ 12 h. The forwards (top left) and reverse (bottom left) equilibration reactions have been displayed alongside a repeat (top right and bottom right respectively).

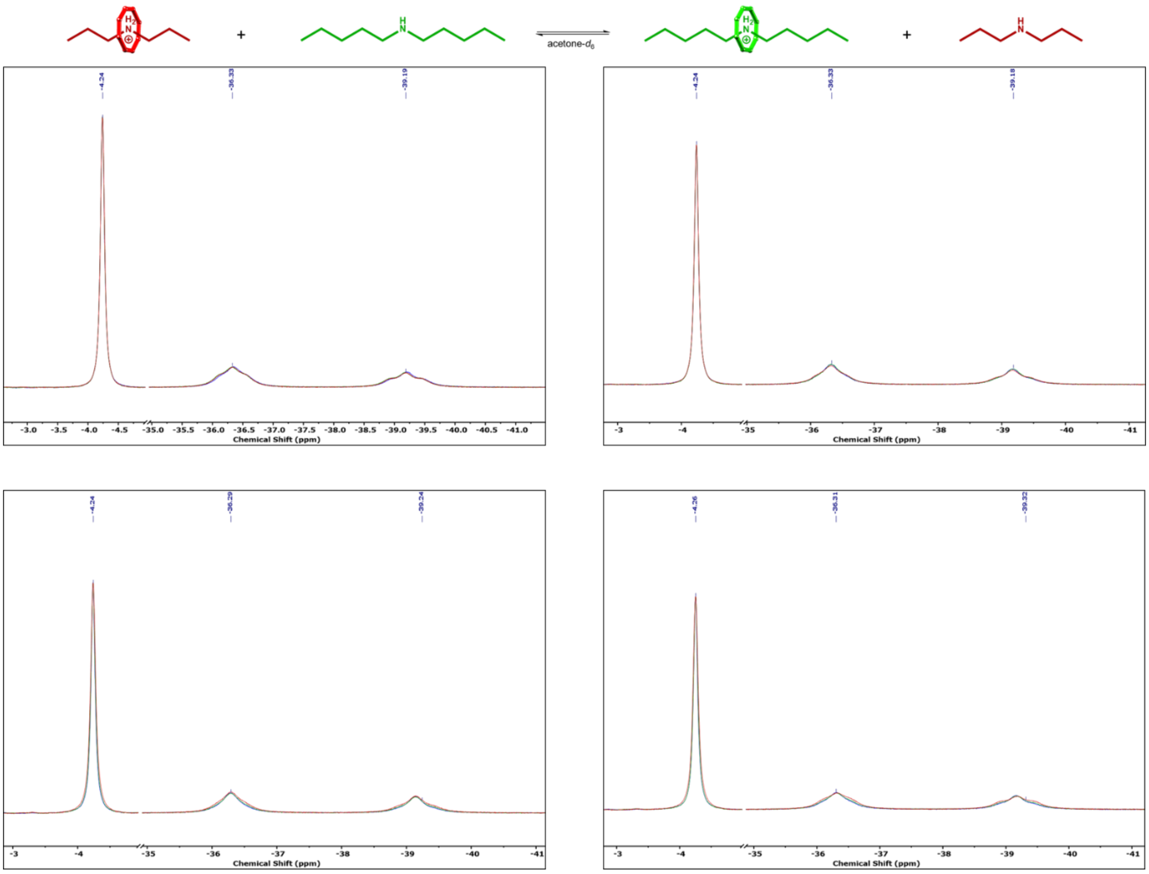

Figure S36. Overlaid normalised ^{1}H NMR spectra (500 MHz, 298 K) showing the signals used for quantification of the concentration of **Pr_2NH_2·1** and **Pe_2NH_2·1** at equilibrium in acetone-d_6 at three time intervals (red, green and blue) separated by ≈ 12 h. The forwards (top left) and reverse (bottom left) equilibration reactions have been displayed alongside a repeat (top right and bottom right respectively).

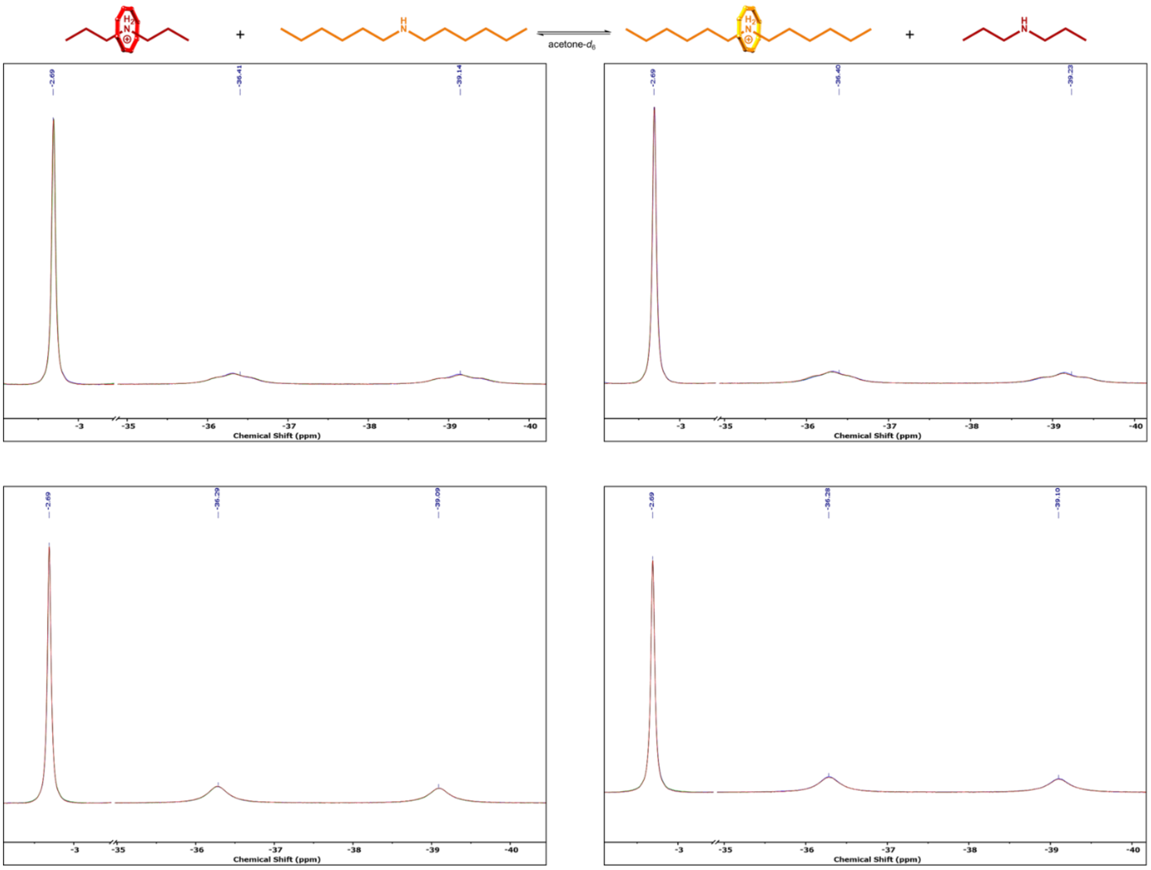

Figure S37. Overlaid normalised ^{1}H NMR spectra (500 MHz, 298 K) showing the signals used for quantification of the concentration of **Pr_2NH_2·1** and **Hx_2NH_2·1** at equilibrium in acetone-d_6 at three time intervals (red, green and blue) separated by ≈ 12 h. The forwards (top left) and reverse (bottom left) equilibration reactions have been displayed alongside a repeat (top right and bottom right respectively).

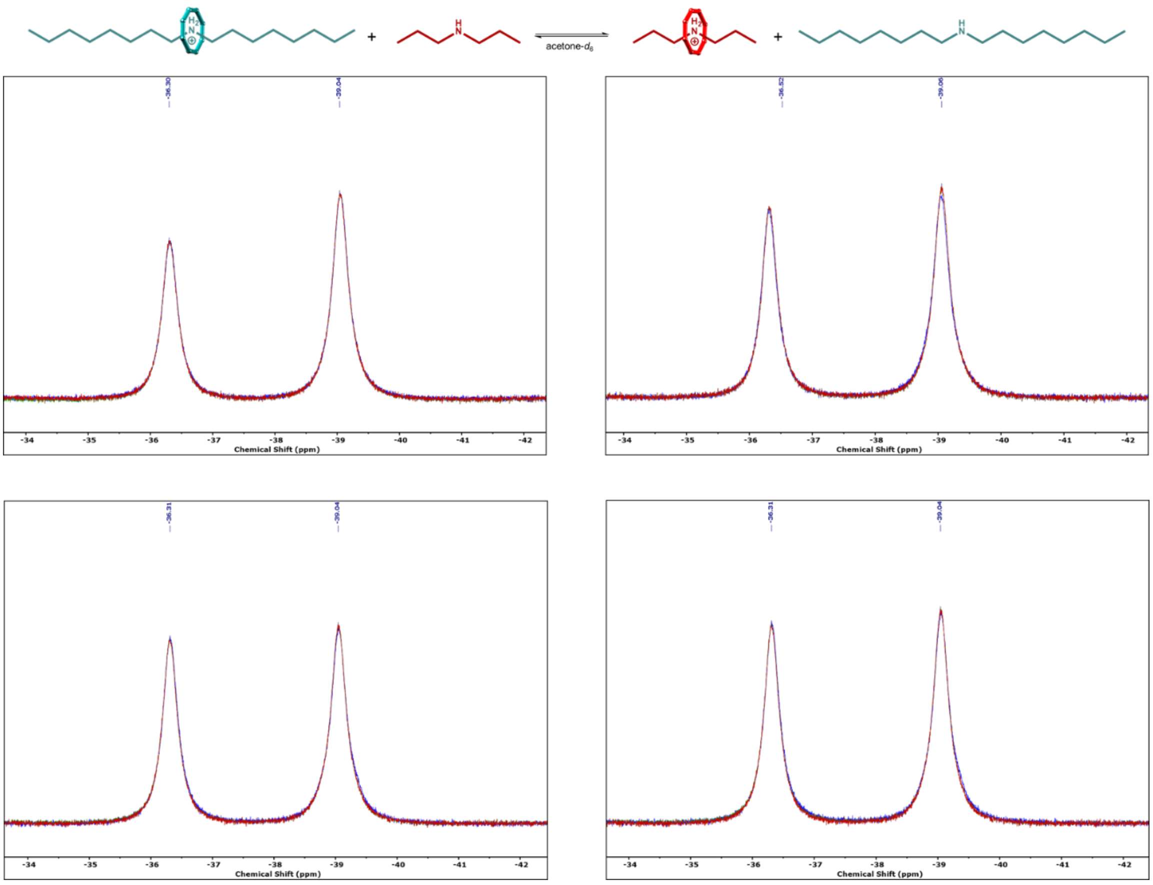

Figure S38. Overlaid normalised ^{1}H NMR spectra (500 MHz, 298 K) showing the signals used for quantification of the concentration of **Pr_2NH_2·1** and **Oc_2NH_2·1** at equilibrium in acetone-d_6 at three time intervals (red, green and blue) separated by ≈ 12 h. The forwards (top left) and reverse (bottom left) equilibration reactions have been displayed alongside a repeat (top right and bottom right respectively).

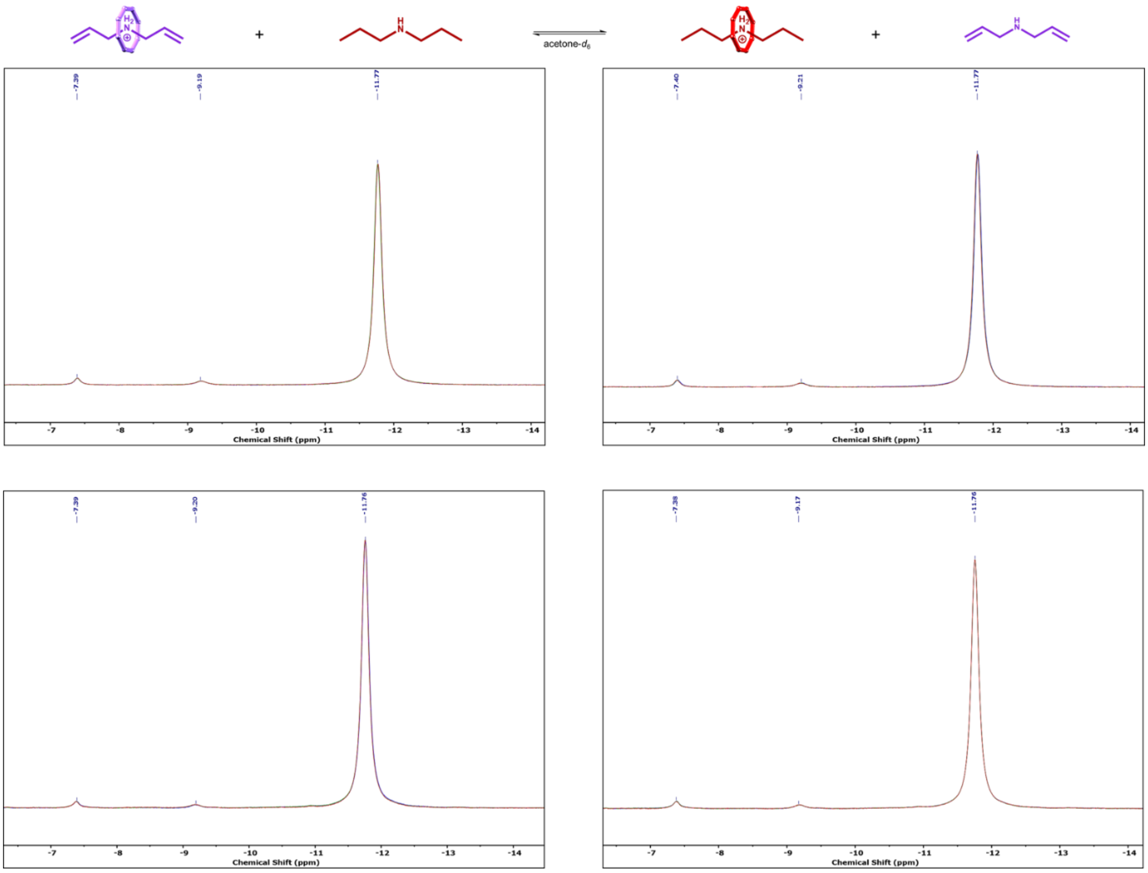

Figure S39. Overlaid normalised ^{1}H NMR spectra (500 MHz, 298 K) showing the signals used for quantification of the concentration of **Allyl$_2$NH$_2$·1** and **Pr$_2$NH$_2$·1** at equilibrium in acetone-d_6 at three time intervals (red, green and blue) separated by ≈ 12 h. The forwards (top left) and reverse (bottom left) equilibration reactions have been displayed alongside a repeat (top right and bottom right respectively).

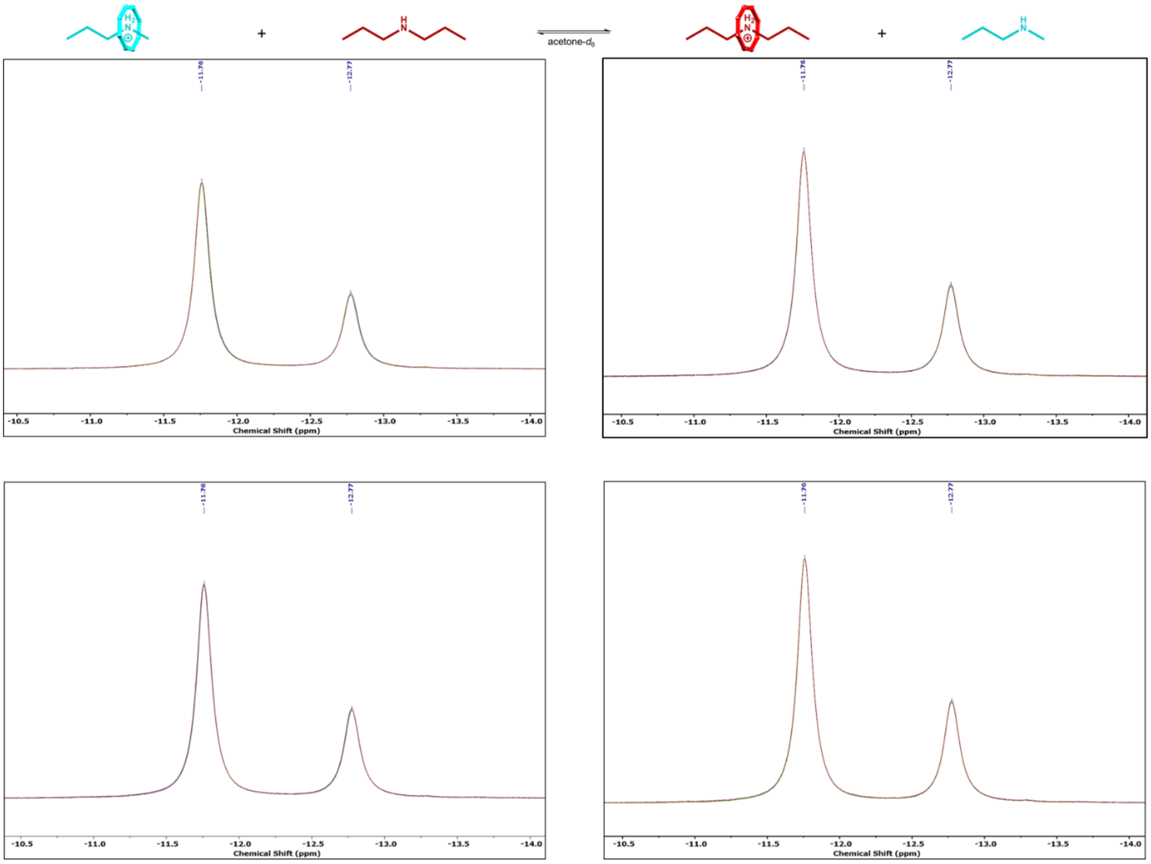

Figure S40. Overlaid normalised ^{1}H NMR spectra (500 MHz, 298 K) showing the signals used for quantification of the concentration of **MeNH$_2$Pr·1** and **Pr$_2$NH$_2$·1** at equilibrium in acetone-d_6 at three time intervals (red, green and blue) separated by ≈ 12 h. The forwards (top left) and reverse (bottom left) equilibration reactions have been displayed alongside a repeat (top right and bottom right respectively).

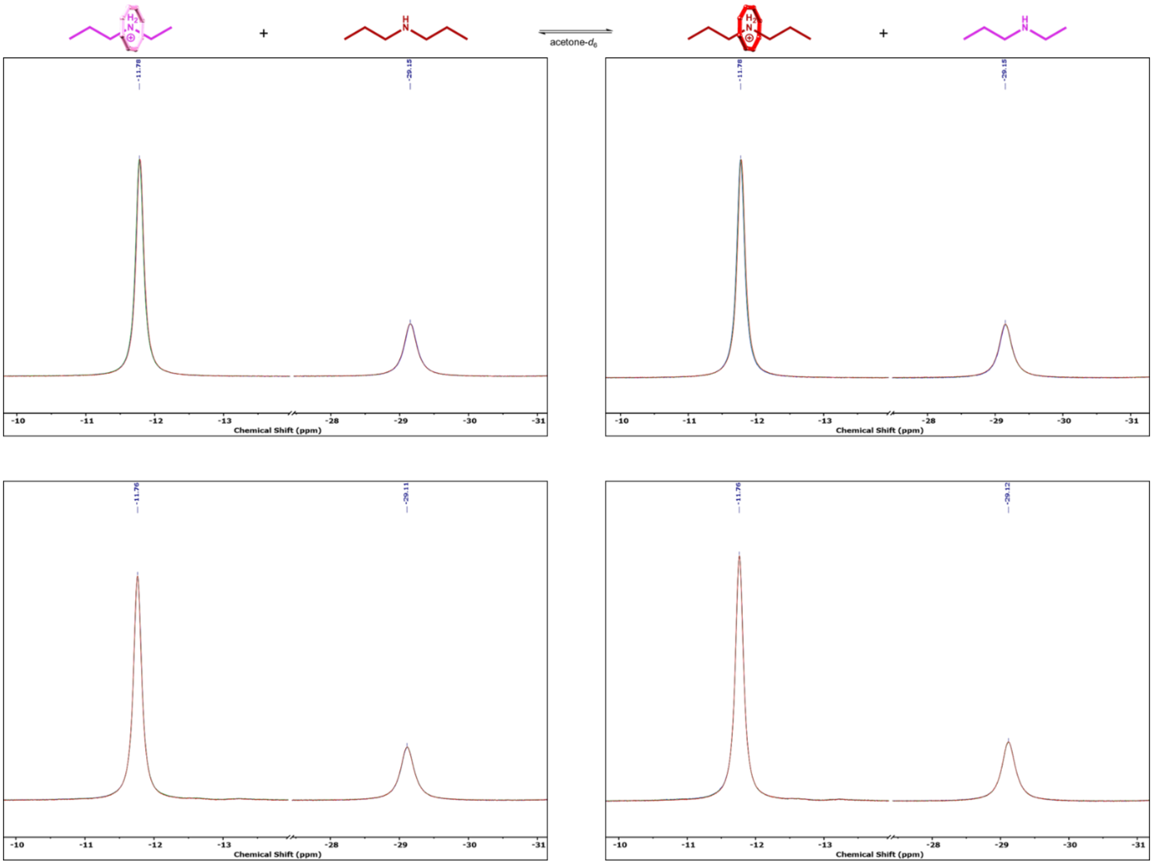

Figure S41. Overlaid normalised ^{1}H NMR spectra (500 MHz, 298 K) showing the signals used for quantification of the concentration of **$EtNH_2Pr·1$** and **$Pr_2NH_2·1$** at equilibrium in acetone-d_6 at three time intervals (red, green and blue) separated by ≈ 12 h. The forwards (top left) and reverse (bottom left) equilibration reactions have been displayed alongside a repeat (top right and bottom right respectively).

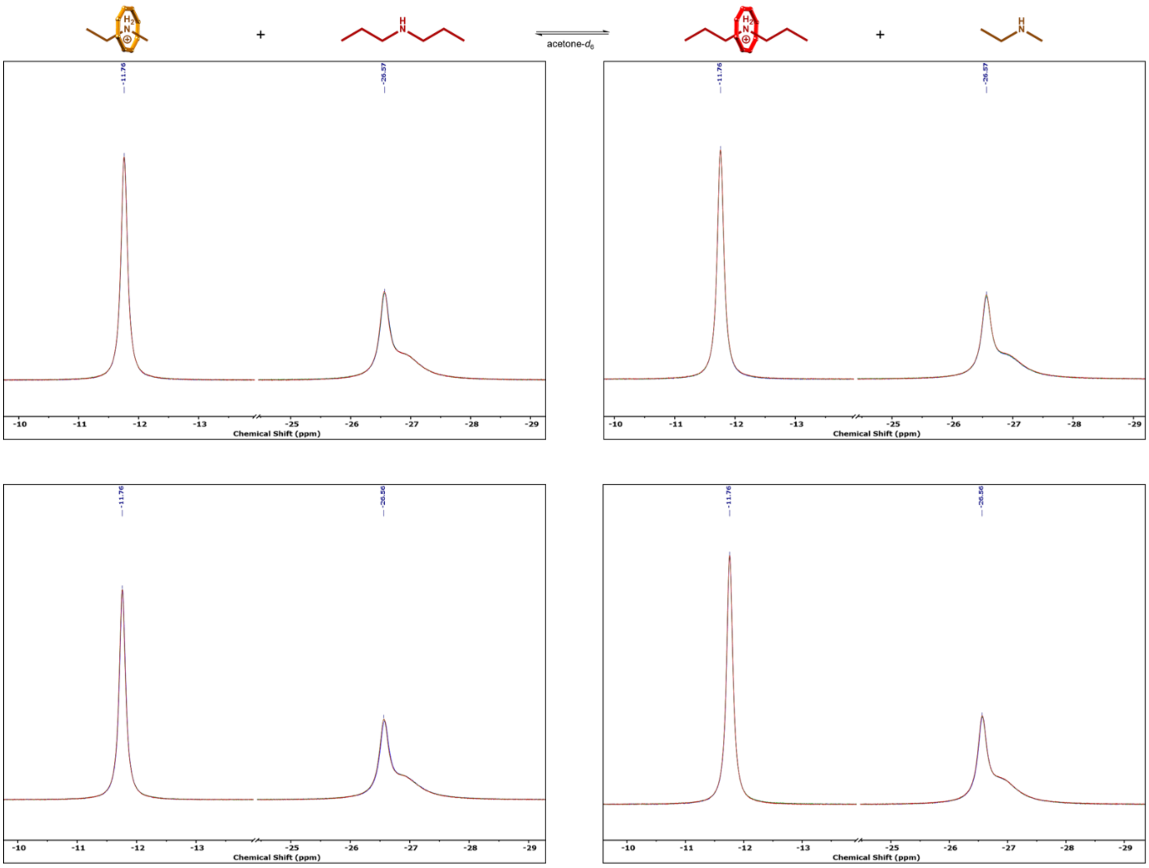

Figure S42. Overlaid normalised ^{1}H NMR spectra (500 MHz, 298 K) showing the signals used for quantification of the concentration of **$EtNH_2Me$·1** and **Pr_2NH_2·1** at equilibrium in acetone-d_6 at three time intervals (red, green and blue) separated by ≈ 12 h. The forwards (top left) and reverse (bottom left) equilibration reactions have been displayed alongside a repeat (top right and bottom right respectively).

5. Crystallography

X-ray diffraction data for **Allyl$_2$NH$_2$·1** were collected on an Agilent Supernova 4-circle diffractometer with a microfocus Mo Kα source and an Eos CCD detector at a temperature of 100 K. X-ray diffraction data for compound **Et$_2$NH$_2$·1**, **Pr$_2$NH$_2$·1**, **Oc$_2$NH$_2$·1**, and **PrNH$_2$·1** were collected using a dual wavelength Rigaku FR-X rotating anode diffractometer using CuKα (λ = 1.54184 Å) radiation, equipped with an AFC-11 4-circle quarter-χ goniometer, VariMAX^{TM} microfocus optics, a Hypix-6000HE detector and an Oxford Cryosystems Cryostream 700 plus nitrogen flow gas system, at a temperature of 100 K. Data were collected and reduced using CrysAlisPro v42.[41] Absorption correction was performed using empirical methods (SCALE3 ABSPACK) based upon symmetry-equivalent reflections combined with measurements at different azimuthal angles.

Structure solution and refinement was carried out using Shelx-2019, implemented through Olex2 v1.5.[42] Least-squared refinements against all F^2 values using ShelXL 2019/3. In all cases, the chromium and nickel in the Cr_7Co rings were assumed to be equally distributed, with each metal site constrained to have a 7:1 ratio of chromium to cobalt, with the associated same coordinate and same atomic displacement parameter constraints.

Table S10. Crystallographic data for **Et$_2$NH$_2$·1**, **Pr$_2$NH$_2$·1**, **Oc$_2$NH$_2$·1**, **Allyl$_2$NH$_2$·1** and **PrNH$_3$·1.**

**Compound**	**Et$_2$NH$_2$·1**	**Pr$_2$NH$_2$·1**	**Oc$_2$NH$_2$·1**	**Allyl$_2$NH$_2$·1**	**PrNH$_3$·1**
Identification code	lrepw532	rrepw480	rrepw488	s6195r	rrepw491
Empirical formula	$C_{102.75}H_{198}CoCr_7F_8NO_{34.75}$	$C_{105}H_{201}CoCr_7F_8N_4O_{38}$	$C_{112}H_{212}CoCr_7F_8NO_{32}$	$C_{105}H_{188.98}CoCr_7F_8NO_{33}$	$C_{100.89}H_{193.54}CoCr_7F_8NO_{33.88}$
Formula weight	2578.54	2702.62	2659.74	2568.47	2537.81
Temperature/K	100.01(10)	100.00(11)	100.01(11)	99.99(10)	99.98(10)
Crystal system	triclinic	monoclinic	triclinic	monoclinic	monoclinic
Space group	P-1	$P2_1/c$	P-1	$P2_1/c$	$P2_1/c$
a/Å	16.2227(5)	19.4455(2)	21.3686(11)	20.0708(3)	20.1492(3)
b/Å	26.8853(10)	30.4994(3)	24.8266(13)	17.8051(3)	17.6563(3)
c/Å	32.4374(7)	24.7226(3)	44.9334(17)	37.9730(7)	37.9275(6)
α/°	71.819(3)	90	103.342(4)	90	90
β/°	89.659(2)	106.9920(10)	97.403(4)	100.120(2)	100.7520(10)
γ/°	86.926(3)	90	101.407(4)	90	90
Volume/Å^3	13421.1(7)	14022.3(3)	22352.4(19)	13359.0(4)	13256.2(4)
Z	4	4	6	4	4
ρ_{calc}g/cm^3	1.276	1.280	1.186	1.277	1.272
μ/mm^{-1}	6.097	5.885	5.492	0.746	6.160
F(000)	5466.0	5720.0	8484.0	5428.0	5376.0
Crystal size/mm^3	0.299 × 0.2 × 0.072	0.57 × 0.43 × 0.21	0.217 × 0.13 × 0.059	0.842 × 0.644 × 0.293	0.686 × 0.326 × 0.119
Radiation	Cu Kα (λ = 1.54184)	CuKα (λ = 1.54184)	Cu Kα (λ = 1.54184)	Mo Kα (λ = 0.71073)	Cu Kα (λ = 1.54184)
2Θ range for data collection/°	3.464 to 133.2	4.728 to 152.338	3.764 to 152.306	3.08 to 61.588	4.464 to 152.234
Index ranges	-15 ≤ h ≤ 18, -31 ≤ k ≤ 32, -38 ≤ l ≤ 38	-24 ≤ h ≤ 24, -38 ≤ k ≤ 37, -31 ≤ l ≤ 31	-26 ≤ h ≤ 24, -22 ≤ k ≤ 31, -53 ≤ l ≤ 56	-24 ≤ h ≤ 21, -24 ≤ k ≤ 18, -40 ≤ l ≤ 47	-25 ≤ h ≤ 22, -22 ≤ k ≤ 20, -45 ≤ l ≤ 47
Reflections collected	171258	165490	209523	107794	75983
Independent reflections	46559 [R_{int} = 0.0946, R_{sigma} = 0.0849]	28981 [R_{int} = 0.0564, R_{sigma} = 0.0404]	87925 [R_{int} = 0.1367, R_{sigma} = 0.1490]	32492 [R_{int} = 0.0275, R_{sigma} = 0.0338]	26726 [R_{int} = 0.0491, R_{sigma} = 0.0561]
Data/restraints/parameters	46559/6703/2951	28981/925/1770	87925/57507/6050	32492/5553/1840	26726/5948/1559
Goodness-of-fit on F^2	1.066	1.042	0.938	1.019	1.034
Final R indexes [I>=2σ (I)]	R_1 = 0.1059, wR_2 = 0.2866	R_1 = 0.0698, wR_2 = 0.1958	R_1 = 0.1098, wR_2 = 0.2987	R_1 = 0.0525, wR_2 = 0.1287	R_1 = 0.0784, wR_2 = 0.2107
Final R indexes [all data]	R_1 = 0.1630, wR_2 = 0.3309	R_1 = 0.0818, wR_2 = 0.2071	R_1 = 0.2775, wR_2 = 0.4252	R_1 = 0.0653, wR_2 = 0.1350	R_1 = 0.0968, wR_2 = 0.2252
Largest diff. peak/hole / e Å^{-3}	2.38/-1.79	1.54/-0.93	0.93/-0.56	1.19/-1.19	1.21/-1.29

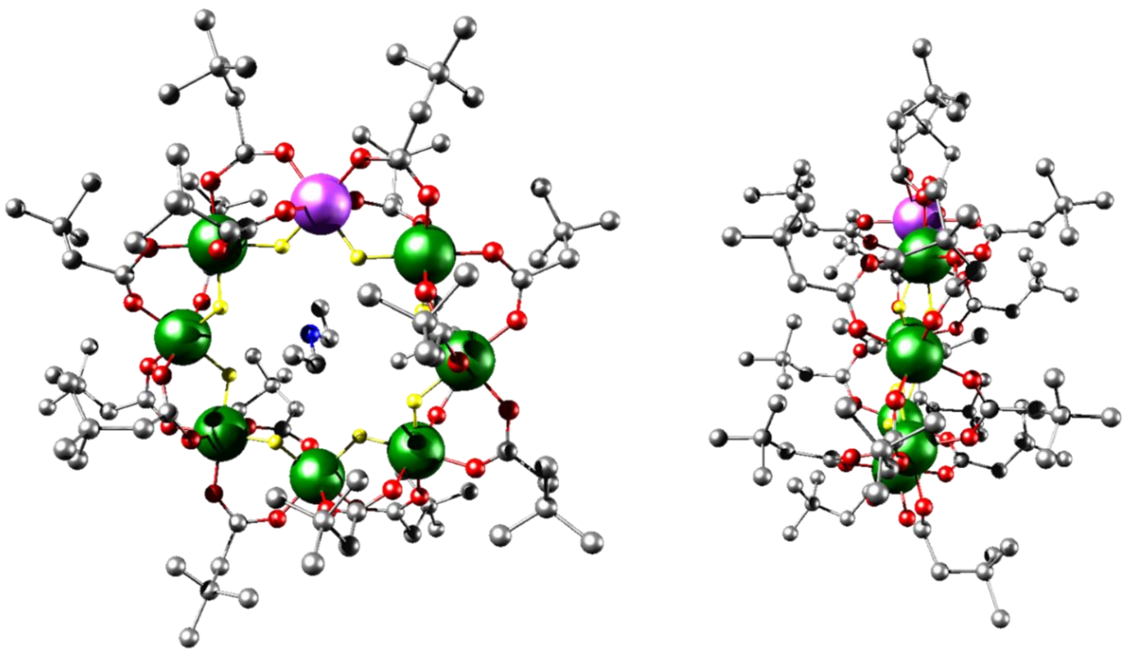

Figure S43. Single Crystal XRD structure of **$Et_2NH_2\cdot 1$**. Colour scheme: Cr dark green, Co lilac, O red, N blue, C grey, F yellow. H atoms have been omitted for clarity. The Co sites in the {Cr_7Co} rings are disordered over multiple positions.

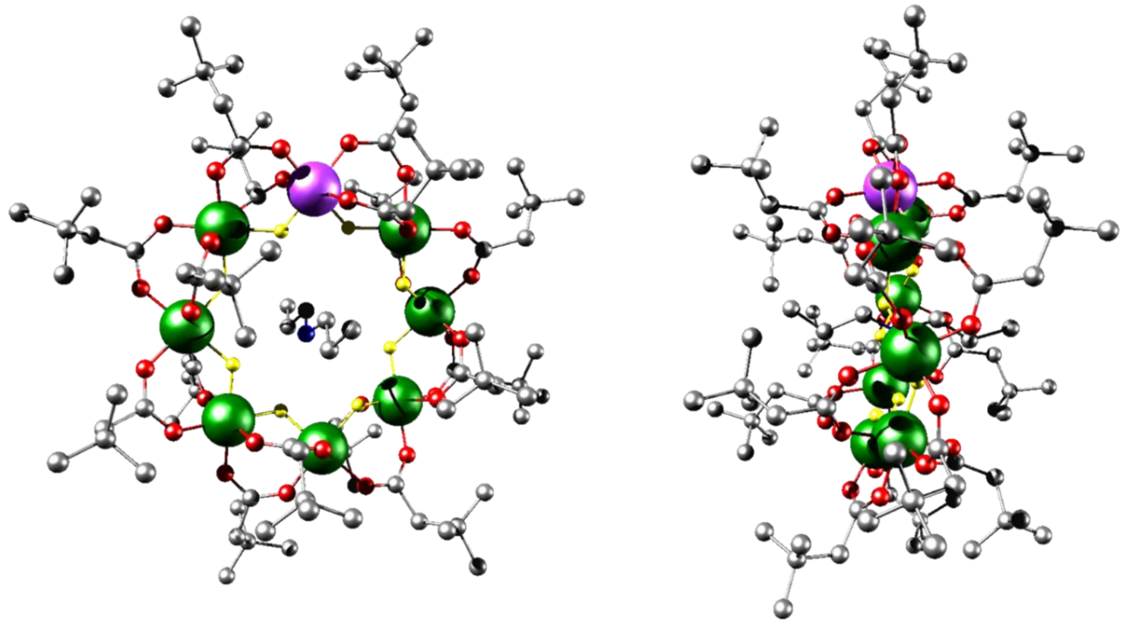

Figure S44. Single Crystal XRD structure of **$Pr_2NH_2\cdot 1$**. Colours as in Figure S34. H atoms have been omitted for clarity. The Co sites in the {Cr_7Co} rings are disordered over multiple positions.

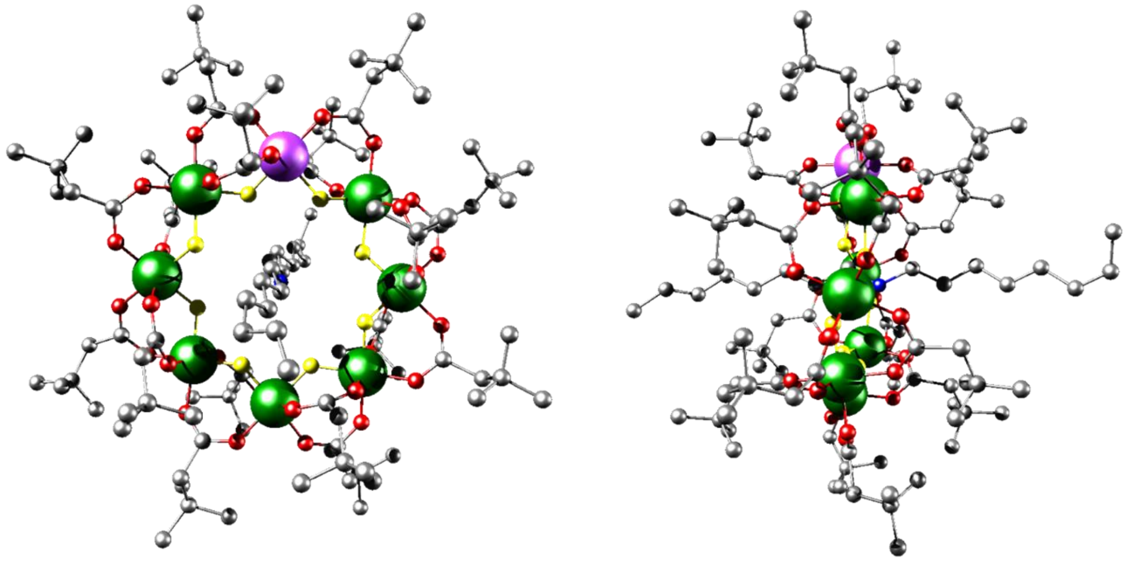

Figure S45. Single Crystal XRD structure of **Oc_2NH_2·1**. Colours as in Figure S34. H atoms have been omitted for clarity. The Co sites in the {Cr_7Co} rings are disordered over multiple positions.

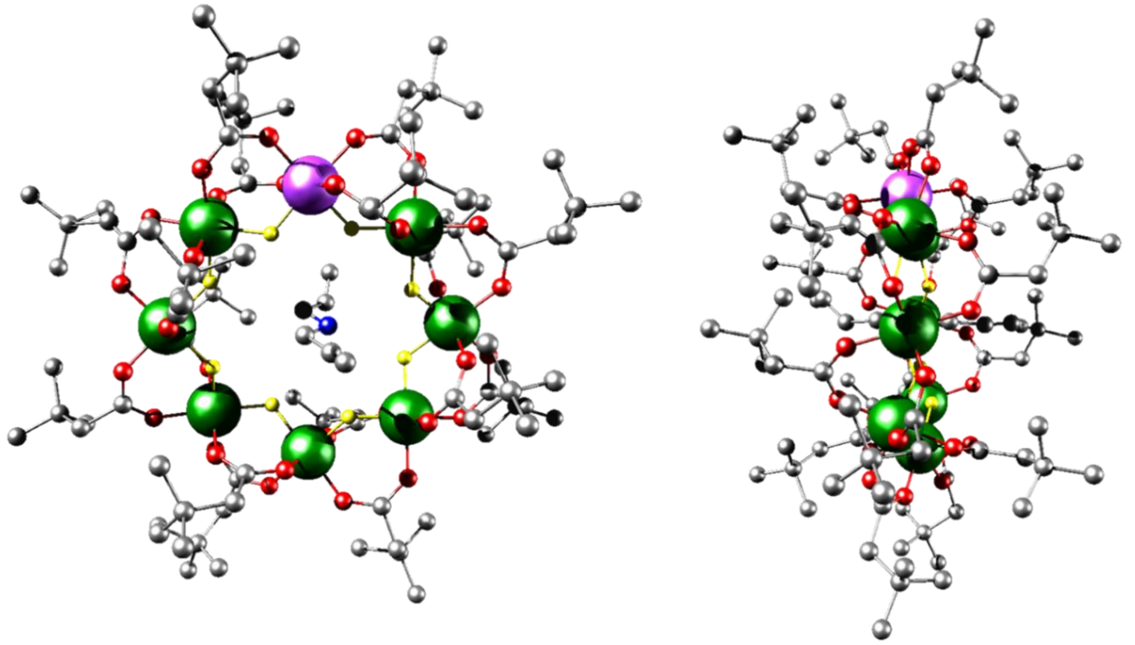

Figure S46. Single Crystal XRD structure of **$Allyl_2NH_2$·1**. Colours as in Figure S34. H atoms have been omitted for clarity. The Co sites in the {Cr_7Co} rings are disordered over multiple positions.

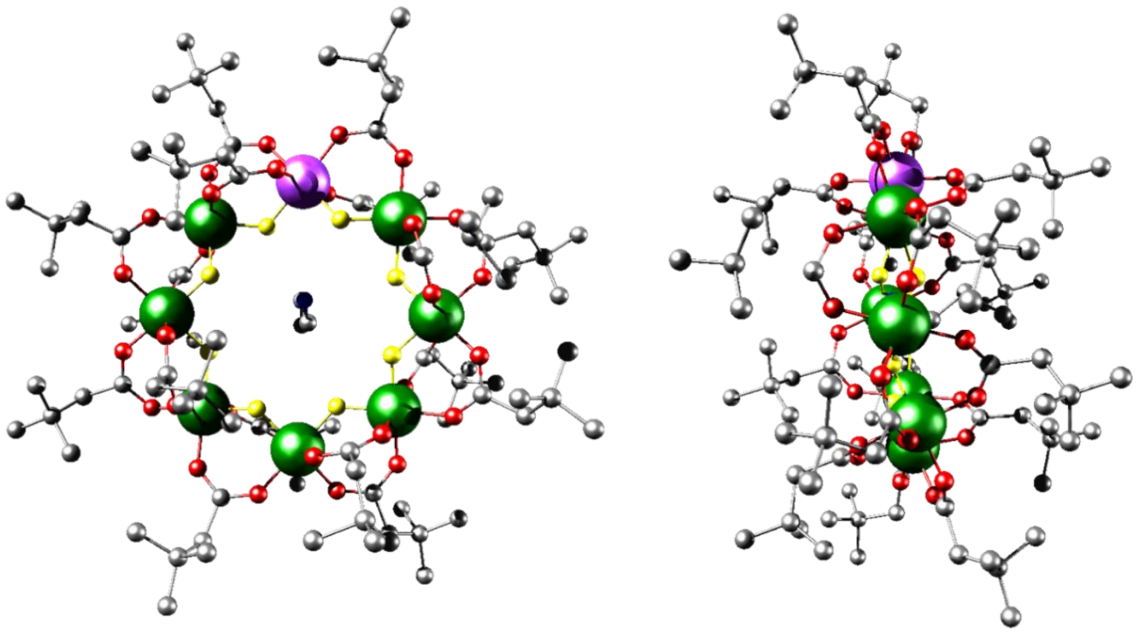

Figure S47. Single Crystal XRD structure of **$PrNH_3 \cdot 1$**. Colours as in Figure S34. H atoms have been omitted for clarity. The Co sites in the {Cr_7Co} rings are disordered over multiple positions.

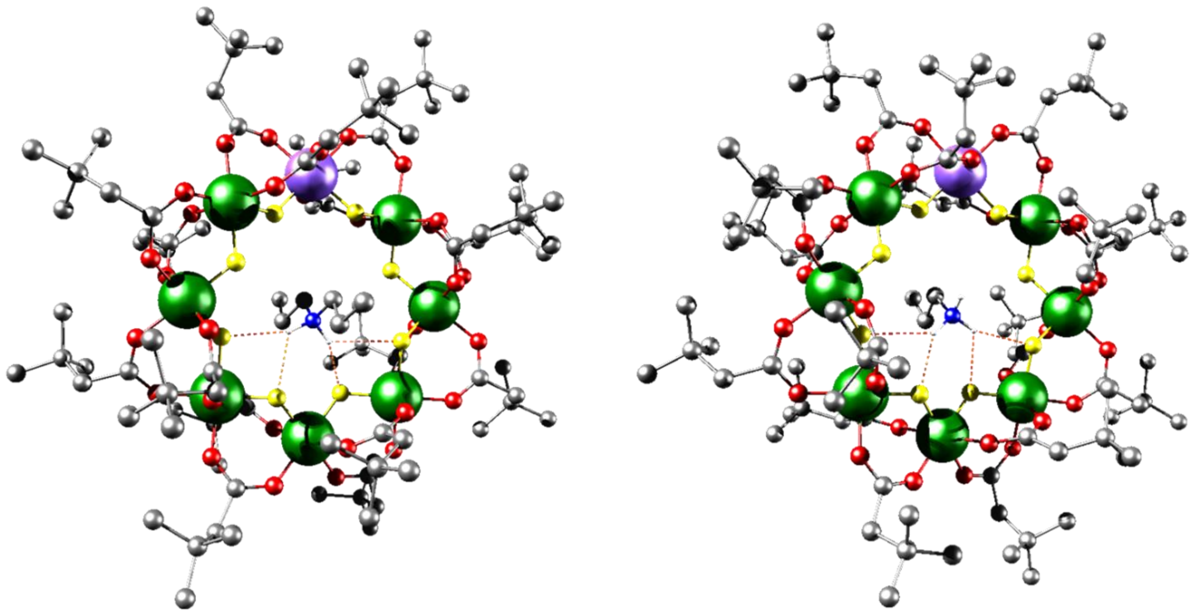

Figure S48. Single Crystal XRD structure of **$Pr_2NH_3 \cdot 1$** (LHS) and **$PrNH_3 \cdot 1$** (RHS). Colours as in Figure S34. H atoms (bound to C atoms) have been omitted for clarity. The Co sites in the {Cr_7Co} rings are disordered over multiple positions. Short (< 2.7 Å) N-H···F contacts have been highlighted in orange.

6. Examples of CID-MS

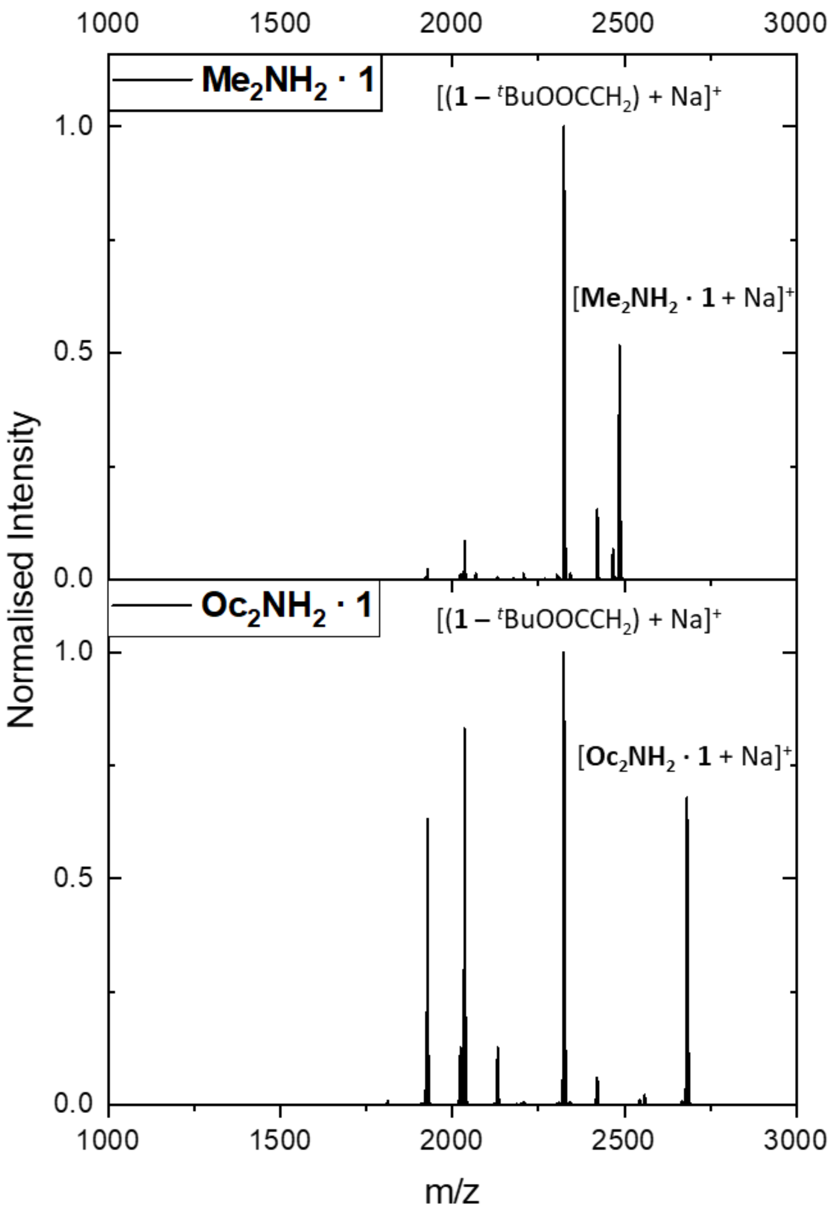

Figure S49: Tandem Mass Spectra of [$\mathbf{Me_2NH_2 \cdot 1}$ + Na]$^+$ (top, collision energy = 60 eV) and [$\mathbf{Oc_2NH_2 \cdot 1}$ + Na]$^+$ (bottom, collision energy = 90 eV). Both (and all other studied) complexes show the same main fragmentation pathway, involving the loss of the ammonium cation and a carboxylate ligand.

7. References

[28] D. W. Hill, C. L. Baveghems, D. R. Albaugh, T. M. Kormos, S. Lai, H. K. Ng, D. F. Grant, *Rapid Commun. Mass Spectrom.* **2012**, *26*, 2303–2310.

[29] T. M. Kertesz, L. H. Hall, D. W. Hill, D. F. Grant, *J. Am. Soc. Mass Spectrom.* **2009**, *20*, 1759–1767.

[30] P. Chakraborty, A. Baksi, E. Khatun, A. Nag, A. Ghosh, T. Pradeep, *J. Phys. Chem. C* **2017**, *121*, 10971–10981.

[31] N. Geue, T. S. Bennett, L. A. I. Ramakers, G. A. Timco, E. J. L. McInnes, N. A. Burton, P. B. Armentrout, R. E. P. Winpenny, P. E. Barran, *Inorg. Chem.* **2023**, *62*, 2672–2679.

[32] N. Geue, G. A. Timco, G. F. S. Whitehead, E. J. L. McInnes, N. A. Burton, R. E. P. Winpenny, P. E. Barran, *Nat. Synth.* **2023**, *2*, 926–936.

[33] S. Hoops, R. Gauges, C. Lee, J. Pahle, N. Simus, M. Singhal, L. Xu, P. Mendes, U. Kummer, *Bioinformatics* **2006**, *22*, 3067–3074.

[39] D. B. Fogel, L. J. Fogel, J. W. Atmar, in *[1991] Conf. Rec. Twenty-Fifth Asilomar Conf. Signals, Syst. Comput.*, IEEE Comput. Soc. Press, **n.d.**, pp. 540–545.

[40] R. Storn, K. Price, *J. Glob. Optim.* **1997**, *11*, 341–359.

[41] CrysAlisPRO, Oxford Diffraction /Agilent Technologies UK Ltd, Yarnton, England.

[42] G. M. Sheldrick, SHELXL-2019, University of Göttingen and Bruker AXS GmbH, Karlsruhe (Germany) 2012–2014.
